# Barriers to disseminating brief CBT for voices from a lived experience and clinician perspective

**DOI:** 10.1371/journal.pone.0178715

**Published:** 2017-06-02

**Authors:** Cassie M. Hazell, Clara Strauss, Kate Cavanagh, Mark Hayward

**Affiliations:** 1 School of Psychology, University of Sussex, Falmer, Brighton, United Kingdom; 2 Research and Development Department, Sussex Partnership NHS Foundation Trust, Sussex Education Centre, Hove, United Kingdom; Pennsylvania State University College of Medicine, UNITED STATES

## Abstract

Access to psychological therapies continues to be poor for people experiencing psychosis. To address this problem, researchers are developing brief interventions that address the specific symptoms associated with psychosis, i.e., hearing voices. As part of the development work for a brief Cognitive Behaviour Therapy (CBT) intervention for voices we collected qualitative data from people who hear voices (study 1) and clinicians (study 2) on the potential barriers and facilitators to implementation and engagement. Thematic analysis of the responses from both groups revealed a number of anticipated barriers to implementation and engagement. Both groups believed the presenting problem (voices and psychosis symptoms) may impede engagement. Furthermore clinicians identified a lack of resources to be a barrier to implementation. The only facilitator to engagement was reported by people who hear voices who believed a compassionate, experienced and trustworthy therapist would promote engagement. The results are discussed in relation to how these barriers could be addressed in the context of a brief intervention using CBT techniques.

## Introduction

Psychosis is a term used to describe a range of unusual experiences that cause distress or impede functioning i.e. delusions, hallucinations, or disorganised thoughts and behaviours [1]. Cognitive behaviour therapy is the only recommended individual psychotherapy for psychosis (CBTp) [2–5]. However, very few patients are able to access this therapy [6], with limited resources often identified as a significant barrier to implementation [7–9]. One approach to improve access could be to offer CBTp using less resources (i.e. fewer sessions). Recent meta-analyses have suggested that brief CBTp produces moderate treatment effects when compared to treatment as usual [10,11].

Consistent with the emerging literature suggesting that symptom-specific therapies (i.e. focusing solely on voices or delusions) may be more effective than broadly-focused CBTp [12,13], we are developing a brief form of CBTp targeting distressing voices (auditory verbal hallucinations) using a guided self-help format. Guided self-help CBT for voices (CBTv) will be based on the CBT self-help book ‘Overcoming Distressing Voices’ [14]. As part of the therapy development process we wanted to learn about the potential facilitators and barriers to therapy implementation from the stakeholders who deliver (mental health clinicians) and receive (people who hear voices) therapy within the NHS.

This paper presents the findings from two consultations: study one explored the perspective of people with lived experience of hearing voices, and study two explored the perspective of mental health clinicians. Both studies aimed to address the following research questions: (1) What are the potential barriers/facilitators to engagement in guided self-help CBTv? (2) What are the potential barriers/facilitators to the implementation of guided self-help CBTv? Identifying potential facilitators and barriers to implementation will enable us to formulate an implementation plan that maximises the possibility of successfully implementing the results of this programme of research

## Study 1: The lived experience perspective

### Methods

#### Design

This study used a focus group methodology and recruited people who hear voices to one of three focus groups, each including between 6–10 participants [15]. All of the focus groups were facilitated using the same discussion guide (available from corresponding author on request) and were audio recorded, then transcribed and analysed using thematic analysis [16].

#### Participants

Inclusion criteria required that participants were aged 18 or over, and had at least one year’s experience of hearing voices, irrespective of psychiatric diagnosis. Diagnosis was not used as an exclusion criteria in response to a number of studies that have reported little to no difference in the experience of hearing voices as a function of diagnosis [17–19]. Exclusion criteria specified that participants must be able to read and write in English.

A total of 21 participants consented to take part in the focus group (see Table 1 for demographic information). They were divided into three focus groups based on their locality.

**Table 1:** Demographic information of participants in study1.

Age *M(SD)*		42 (11.12)
Gender %	Male	47.6
	Female	42.9
	Other	9.5
Diagnosis %	Schizophrenia	52.4
	Depression	14.3
	Schizoaffective Disorder	9.5
	Borderline Personality Disorder	9.5
	Dissociative Identity Disorder	4.8
	Did not know	9.5
Number of years hearing voices *M(SD)*		17.43 (14.52)
Age voices started M(SD)		24.57(14.54)

Participants’ psychiatric diagnosis was self-reported.

#### Discussion guide

The discussion guide structure was based on the recommendations of Greenbaum [20] and its content was based on the 4 Ps model [21]. The 4 Ps model identifies four factors that can facilitate or hinder intervention engagement: specifically, (1) programme—the intervention e.g. structure, length, style; (2) problem—the presenting mental health difficulty e.g. complexity, severity, comorbidity; (3) person—individual differences e.g., demographics, patient expectations; and (4) provider—the intervention delivery e.g. amount and quality of therapist contact. Participants were also invited to share any other thoughts about the intervention at both the start and end of the discussion. Each focus group was scheduled for 90 minutes.

#### Procedure

Participants were recruited through four sources: (1) via clinician referrals from local NHS mental health services; (2) a research database of staff, patients and carers who have consented to be contacted about research studies; (3) the third sector, including mental health charities and hearing voices support groups; (4) self-referral in response to advertising materials. Potential participants were given the study information, and at least 24 hours to decide if they would like to take part. Those who decided to take part, met with the first author to discuss the study, provide consent, and receive a copy of the self-help book the intervention would be based on: Overcoming Distressing Voices [14]. Participants were asked to read any chapter of the book prior to the focus group. There was a minimum time lapse of two weeks between receiving the book and attending the focus group for all participants.

Each of the focus groups was facilitated by the first author, with support from a research assistant. All of the participants were paid £15 for their consultation services.

#### Ethics

Ethical approval was granted by the Research Ethics Committee South East Coast—Surrey (REC reference: 14/LO/1880). Local governance approval was given by the Kent, Surrey and Sussex Clinical Research Network. This research was sponsored by the University of Sussex. Participants were required to give informed consent in writing prior to participating in this study.

#### Analysis

All of the recordings were transcribed by the first author into QSR International’s NVivo 10 software. At the point of transcription, all identifiable information was removed, and replaced with pseudonyms where necessary. The transcripts were analysed by the first author using thematic analysis, in line with the Braun and Clarke [16] protocol. This six-stage approach to analysis involved immersion within the data, coding of the smallest units of meaning within each transcript, and the clustering of codes into themes and sub-themes within and across transcripts. The subsequent refinement and naming of the themes involved the constant interplay between data, codes and themes, ensuring the final results were grounded in participants’ views. All of the participants consented to their direct quotes being used within this report.

The codes and themes were shared with the rest of the research team to assess the credibility of the analysis. The transcript was also shared with an independent group of doctoral researchers studying qualitative methods. They double coded an excerpt of the transcript (approximately a third), and verified the credibility of the themes derived. There were no points of disagreement concerning the content and meaning of the themes derived, although some of the theme labels did differ. Where differences occurred these were discussed until a consensus was reached.

### Results

Seven main themes emerged from the analysis, containing 20 sub-themes (Table 2). Two themes (‘the therapist’ and ‘the presenting problem’) will be described in full as they pertain specifically to the research question; these are supported by illustrative quotes. The remaining themes detail the participant’s opinions of the intervention protocol, and materials (e.g. self-help book). For the full qualitative analysis (with illustrative quotes) please see the supplementary material.

**Table 2:** Results of thematic analysis for studies 1 and 2.

	Main Themes		Sub-Themes	Example Quote
*Study 1 (lived experience)*				
1		The Self-Help Book	Positive feedback	‘Yeah referring back to this [the book], um I think that I can relate to all of what’s in here um which is quite amazing reading it.’
			Negative feedback	‘It takes a very basic level of um sort of voice hearing and it can be a lot more complex than that.’
			Self-reflection	‘I think the way it worked for me I started having some sort of intuitions, the voices saying this and that and it expanded, and it confirmed my suspicions.’
2		Therapy Protocol	Self	‘Self-esteem is important because if your self-esteem is really low then you’re less likely to be able to challenge your voices because um I think you give them more power. ‘
			Voices	‘I think the voices themselves are not as bad as the thing they can do with you in terms of how you respond. I mean it could be self-neglect or some other things.’
			Relationships	‘It’s all down to the way you reply to them [voices], even verbally, so if you improve your relationship with your voices you will then improve your relationship with the outside world.’
			Coping Strategies	‘I have learned coping mechanisms. I will say ‘oh yes I can’ which has given me a bit of strength.’
3		The Therapist	Personal qualities	‘Having the compassion to want to help, not just because they’re interesting to work, with but because you know they do have that passion to want to help people.’
			Therapist skills	‘I think what I was going to say is someone who knows their stuff but doesn't have the arrogance they think they know it all.’
			Confidentiality in therapy	‘I think therapy is therapy and it stays completely confidential in therapy, and that’s a relationship with my therapist.’
4		Pragmatics of the Therapy	Therapy Structure	‘I would prefer to do it [therapy] one to one because then you can talk more. People won’t pressure you to talk about things that you don't want to talk about.’
			Timing	‘You need to have a certain level of wellness in order to engage with the book.’
5		The presenting problem	Voices as saboteurs	‘If I’m focussing on something that is specifically about hearing voices and how to help that situation, my voices will not like that.’
			Cognitive processes	‘I find it really hard to read um at the best of times let alone when my concentration is down because I’m more unwell um.’
6		Networks	Clinical relationships	‘I have contacted A&E, I have contacted my mental health worker, and they have done absolutely jack sh*t about it.’
			Nonclinical relationships	‘Like my family are in denial still, so I will tell them something that's been going on and they think it’s nothing, its fine.’
			Stigma	‘I was thinking because I was on a packed bus with it [the book] and I thought if anyone asks me I will tell them I am a psychologist.’
			Group dynamics	‘I think it’s quite amazing. I think you’re quite special to have experienced voices for such a long period of time and still be here.’
7		Therapy Flaws	Theory	‘It says that ‘hearing voices in itself is not a problem’ but I can’t agree with that because hearing voices itself is a problem.’
			Missing elements	‘This just concentrates on voices, but usually there is a lot more symptoms that come along when… I know that when I have been ill there is a lot more going on.’
*Study 2 (clinician)*				
1		Positive attitude toward therapy	GSH in the context of IAPT	‘It could help increase access to therapy which is at the moment very poor.’
			Staff willingness to be involved	‘My desire to be involved in this project is very high, the aim of the project is sound and patient focused.’
2		Negative attitude toward therapy	Not a stand-alone treatment	‘It could be a co-treatment.’
			GSH not an equal treatment option	‘I would be concerned that guided self-help is used in place of face to face therapy.’
3		Support for therapy with a caveat	Importance of clinician training	‘The provision of Self-directed CBT in voices needs the support and backup of trained staff to ensure patient safety.’
			Need for evidence	‘I would be a bit wary about offering CBT self-help for distressing voices as part of routine clinical practice, as the evidence isn't really there.’
4		The presenting problem	Symptoms	‘May be issues around engagement as many of the people on our unit who hear voices often don't have insight into their illness or are acutely unwell.’
			Cognitive Abilities	‘Due to some client’s lack of motivation, I feel that giving them a self-help guide is not necessarily the way forward.’
5		Practical Barriers	Lack of resources	‘All staff are asked to do unrealistic amounts of work, and this [guided self-help CBTv] may simply need too much time.’
			Conflict with service priorities	‘I think there will be resistance from practitioners who rely solely on the medical model.’

GSH = Guided self-help; IAPT = increasing access to psychological therapy.

#### The therapist

There was consensus across all of the focus groups as to the ideal therapist to deliver this intervention. The subthemes outline the characteristics that were important to participants if they were to engage in guided self-help CBTv.

1. *Personal Qualities*:

The first criteria for a therapist discussed across all the focus groups was related to their personal qualities and traits:

Jillian: ‘It's about being able to trust the person [therapist] that you’re with and um to get a good rapport going and to allow somebody to allow you to help you change you know.’

Jillian acknowledged the importance of building a relationship with the therapist and the importance of their role in the process of change that may occur during therapy.

Jimmy: ‘Having the compassion to want to help, not just because they’re interesting to work with but because you know they do have that passion to want to help people.’

Bobby: ‘Someone who is kind and compassionate. Someone who can use empathy, you know, because it’s when you talk to all these doctors and psychiatrists you feel like they can’t relate to you, because they are sat in the chair and they’re professionals and they’re reading from their textbooks, you know… But just someone who is kind and compassionate, someone whose can understand you.’

These comments demonstrate the importance of the non-specific elements of psychological therapy, and importance of developing a genuine and humanistic therapeutic relationship. The word ‘compassion’ was used by both Jimmy and Bobby. These responses give some insight into the importance of rapport when working with people who hear voices.

2. *Therapist Skills*:

Another important criteria that was identified by two out of the three focus groups was the need for the therapist to be skilled:

Nikki: ‘Fairly qualified stuff as well isn’t it. It’s not just a therapist is it, it’s fairly qualified stuff.’

Tim: ‘I have found that the people with specific interests and training in working with voices um are able to extract information from me in really helpful ways, and steer conversations to bring it back to the voices and not going off on a tangent and getting lost somewhere else.’

The comments suggest that Nikki and Tim believe they could not be helped by just any therapist or mental health clinician. Firstly it is important the therapist is sufficiently qualified to deliver therapy more generally (i.e. CBT trained), and secondly they should have specialist knowledge about voices and how to deliver therapy for this client group.

Jimmy: ‘I think what I was going to say is someone who knows their stuff but doesn't have the arrogance they think they know it all. So kind of what Tim was saying about um, so having that specialism, and yeah have that experience of working with people who hear voices.’

These responses imply that the participants feel voice hearing is a complex mental health difficulty that requires a therapist to be skilled and have experience of working with people who hear voices. This theme has the potential to be at odds with the first theme (Personal Qualities), as illustrated by Jimmy: it is important for the therapist to be a skilled therapist and have specialist knowledge of voices, but it is of equal importance that this knowledge does not become ‘arrogance’.

3. *Confidentiality in Therapy*:

This sub-theme was not specific to guided self-help CBTv, but a reflection of the concerns that many participants had about what constituted confidentiality in psychological therapies more generally:

Nikki: ‘I think therapy is therapy and it stays completely confidential in therapy, and that’s a relationship with my therapist. The relationship with my psychiatrist who gives me medication is different and I think I would probably trust my psychiatrist. I would probably tell him some things but not necessarily tell him everything I do in therapy. So therapy is therapy and that's a different thing.’

The client-therapist relationship was described as being distinct and separate from the relationships that participants would have with other mental health practitioners (e.g. psychiatrist), even though, for these participants, they would be working for the same health service. Consequently, they believe the rules around confidentiality and the sharing of information should be distinct.

Abe: ‘For me your caveats around confidentiality will always be the most important because I’ve just had a problem with [name of an organisation]. I used to go in there, you know and um I told the girl I had stopped taking a particular medication and she said well I will need to inform your doctor of that, and I said I’m not sure you do because you said to me at the start of these sessions that everything I say to you is confidential and she said but I had to balance that against my duty of care… I was telling her I was stopping taking one of the trivial medications, and I said to her if you do contact my doctor I will disengage with your organisation and she did, so I did.’

Jeff: ‘It depends what happens really. It depends what the outcome of the um course was like. Um I might decide that there is some stuff I didn't want [my care coordinator] to know but I don't know what’s going to come out. I don't know how successful it’s going to be.’

It is clear that breaking confidentiality is likely to threaten engagement with the therapy. Confidentiality was often discussed in relation to past negative experiences where the ‘rules’ had been unclear around confidentiality. This is a problematic issue for the therapist who is bound by the duty of care and confidentiality policies of their employer (e.g. NHS). Complete confidentiality, as requested by many of the participants, is largely not possible within public healthcare settings. Where these rules cannot be negotiated, the participants wanted total, upfront transparency around the rules. This theme has some synergy with the first theme (Personal Qualities) as being clear about confidentiality will help to foster a trusting therapeutic relationship.

#### The presenting problem

Participants were asked if they thought there was anything that might hinder their engagement in guided self-help CBTv. Two barriers related to the presenting problem were identified across all three of the focus groups:

1. *Voices as saboteurs*:

Although the aim of guided self-help CBTv would be to reduce the distress associated with the experience of hearing voices, many participants felt that the voices themselves could get in the way:

Tim: ‘If I’m focussing on something that is specifically about hearing voices and how to help that situation, my voices will not like that. And they will try and distract me from that or find a way around it um so that it doesn't make sense, um they don't like being talked about.’

Mia: ‘What I also find distressing is if I’m talking to someone, I’m seeing my support worker, and I’m talking about my voices; the voices don't like me talking to people about them so they will say things like tell [them] to ‘eff off’ and I hate that… that's not me you know.’

Both Tim and Mia expressed concerns that their voices would react negatively to being talked about or questioned within therapy. Even though the therapy aims to reduce the distress associated with hearing voices, participants were worried that voices could actually get worse, or at the very least sabotage attempts to engage in the therapy. Ways to address this potential backlash from the voices needs to be considered so as to facilitate engagement.

2. *Cognitive Processes*:

In addition to voices, many of the participants reported experiencing cognitive difficulties, and believed this could make it difficult to engage in guided self-help CBTv.

Nikki: ‘I have read it [the self-help book] once and I am reading it again because there’s bits in it that I have forgotten, because I have short term memory loss. So yeah I am reading it again and I’m finding bits in it I obviously missed the first time around.’

This barrier to engagement identified by Nikki is arguably of greater importance when considering this guided self-help CBTv intervention that makes use of a self-help book. Guided self-help CBTv requires more independent work compared to traditional CBTp. Nikki did not report having any difficulty in reading the book (i.e. comprehending the words); the problem for her was retaining the information. Within an intervention that requires reading as a homework task there needs to be some consideration of how this task can be modified or adapted for those who, like Nikki, experience memory difficulties.

Tim: ‘Another thing I thing I find, especially if I’m unwell is concentration. I find it really hard to read um at the best of times let alone when my concentration is down because I’m more unwell um. I re-read the same thing so that could be a barrier to accessing the therapy.’

Sue: ‘I agree with that. That’s why I couldn't finish this book because I read it over and over, and haven’t got the concentration to read it all very quickly.’

All of the participants speak of reading the same passage multiple times to try and take in the necessary information. Where the required reading for the guided self-help CBTv intervention is substantial, this would be time consuming and potentially frustrating for the client—which is likely to impede engagement. It is important to note that these participants describe their cognitive difficulties as fluctuating states. Consequently, consideration should be given to the changeable nature of cognitive abilities, and the techniques that can be used to compensate for any periods of cognitive difficulty.

### Study 1 discussion

The findings from study 1 demonstrate that people who hear voices were worried that guided self-help CBTv could make their voices worse. Additionally cognitive difficulties were thought to be a potential barrier to engagement. One potential facilitator identified was a therapist whom took the time to create a positive therapeutic relationship based on respect and trust. Study 2 will identify whether mental health clinicians anticipate any of the same barrier or facilitators that were identified within study 1.

## Study 2: The clinician perspective

### Method

#### Design

This study used a questionnaire design. We collected both quantitative and qualitative data, however only the qualitative data are presented here. The quantitative data has been used to assess the factorial validity of the NPT model [22], and is reported in a separate paper. All participants completed the same questionnaire, and the qualitative data was collected using free-text response boxes.

#### Participants

Inclusion criteria required that participants were mental health clinicians working in an NHS trust in the South of England, whom had experience of either delivering psychological therapy and/or working with people who hear voices. This meant that all responses were grounded in some level of expertise. A total of 201 participants completed the questionnaire, and 124 (62% of the sample) provided qualitative data using the free-text response boxes (Table 3).

**Table 3:** Participant characteristics in study 2.

Age (years)*M(SD)*		43.07 (10.99)
Gender %	Male	25.8
	Female	73.4
	Prefer Not to Say	0.8
Team %	Primary Care	4.8
	Secondary Care	88.7
	Early Intervention in Psychosis	6.5
Profession %	Psychological Therapist	33.1
	Psychological Wellbeing Practitioner (PWP)	1.6
	Mental Health Professional	56.4
	Support Worker	8.9
Duration in Profession (years) *M(SD)*		13.70 (10.56)
Experience working with people who hear voices %	A lot to moderate	84.7
	Little to none	15.3

PWP = psychological wellbeing practitioner.

#### Questionnaire

The questionnaire was based on the Normalisation Process Theory (NPT) [22]. NPT is comprised of four factors that are intended to explain the components needed to successfully implement a new idea into clinical practice. Participants were asked a free-text response question related to each factor: (1) coherence—attitudes towards the concept (What do you think about the idea of offering CBT for distressing voices using guided self-help?), (2) cognitive participation—willingness to be involved (How willing would you be to be involved in the development of guided self-help CBT for distressing voices?), (3) collective action—feasibility of implementation within the current system (How feasible do you think it would be to implement guided self-help CBT for distressing voices in the Trust?), and (4) reflexivity—evaluation of the idea (How should guided self-help CBT for distressing voices be evaluated?). All of the free-text boxes were optional, and participants could write as much or as little as they wanted to.

#### Procedure

Participants were recruited via internal emails, publicity, and in-person at NHS Trust events. Participants could complete the questionnaire either online (using Bristol Online Survey) or on paper. All responses were collected anonymously. Participants were informed that consent would be assumed when they returned the completed questionnaire. Participants could complete the questionnaire at a time and place that was convenient to them.

#### Ethics

Ethical approval was granted the Sciences and Technology C-REC at the University of Sussex (Reference: ER/CH283/4). Local governance approval was given by the Sussex Partnership NHS Foundation Trust Research and Development Department Kent. Participants were informed that consent was assumed if they returned their completed questionnaire to the research team.

#### Analysis

All identifiable information was removed prior to analysis, and replaced with pseudonyms where necessary. The data was analysed using the same process and method described for study 1. The credibility of the findings were assessed using the process advocated by Chadwick and colleagues [23]. Firstly codes and themes were shared and discussed within the wider research team. Secondly a random sample of 20% of the excerpts were double-coded by an independent researcher. The level of inter-rater agreement was 85.19%. The transcript was also shared with an independent group of doctoral researchers studying qualitative methods. They double coded an excerpt of the transcript (approximately half), and verified the credibility of the themes derived.

### Results

Five main themes emerged from the analysis, containing 10 sub-themes (Table 2). Two themes (‘the presenting problem’ and ‘practical barriers’) will be described in full as they pertain specifically to the research question; these are supported by illustrative quotes. The remaining themes detail the participant’s opinions of guided self-help CBTv and justification for these opinions. For the full qualitative analysis (with illustrative quotes) please see the supplementary material.

#### The presenting problem

Many of the clinicians expressed some concerns about the suitability of guided self-help CBTv for this client group. Participants identified barriers that related to the presenting problem:

1. *Symptoms*:

The first patient barrier identified was the view that the mental health symptoms experienced by people who hear voices could hinder engagement in the therapy:

‘I would consider this [guided self-help CBTv] to be helpful although unsure whether this would be aimed at clients with less distress as a result of their psychotic symptoms.’

This first response suggests that the clinician believes that assessing suitability for guided self-help CBTv should be done so based on symptom severity—with those experiencing more severe symptoms being unsuitable for therapy. The implication of this is that the clinician seems to believe that not everyone whom hears voices should be offered this therapy. This may lead to clinicians’, rightly or wrongly, acting as gatekeepers for their clients.

‘May be issues around engagement as many of the people on our unit who hear voices often don't have insight into their illness or are acutely unwell.’

‘It is potentially asking a great deal of the patient to go through this course given that there is a greater proportion of people who experience distressing voices who also lead chaotic lifestyles.’

For these clinicians, it appears that hearing voices in itself is not necessarily a barrier to engagement. Instead it is what the clinicians associate with the experience of hearing voices that may be the barrier i.e. lack of ‘insight’ and ‘chaotic lifestyles’. These clinicians however do not explain the basis for these associations or how these factors may act as barrier. For example, assuming the client does have a chaotic lifestyle, how will this prohibit engagement in guided self-help CBTv?

2. *Cognitive Abilities*:

Clinicians also discussed the role of cognitive processes and motivation, and consequently whether this client group would be able to engage with an intervention that requires an element of self-help, due to the cognitive skills it requires:

‘Due to some client’s lack of motivation, I feel that giving them a self-help guide is not necessarily the way forward.’

‘My experience of this group are that they will require a considerable degree of support to undertake this work, due to high levels of anxiety, and often low levels of self-organisation skills.’

‘The effect of long term medication, impaired cognitive ability learning disability, not only for focus and concentration, but also for processing information and transferring skills into daily life’

It is implicit here that clinicians associate the experience of hearing voices with poor motivation and cognitive impairments, and consequently identify this as a barrier to therapy engagement. Some clinicians did not attribute these cognitive difficulties to voices themselves, but a by-product of ‘medication’ or lifestyle factors. Much like the ‘Cognitive Processes’ sub-theme from study 1, the influence of cognitive abilities on engagement is potentially a more prominent point to consider because of the increased level of independent work required within a guided self-help intervention compared to ‘traditional’ CBT.

#### Practical barriers

As well as the patient-related barriers, clinicians identified a number of practical barriers that they foresaw would impede the implementation of guided self-help CBTv. The practical barriers identified are discussed below:

1. *Lack of Resources*:

The most common barrier discussed by clinicians was a feeling that they do not currently have enough resources to be able to implement guided self-help CBTv. It was apparent that most felt they would either need protected time or more staff to be able to support implementation:

‘Despite years of highlighting the resourcing issues on in-patient wards we still do not have enough resources to give time to offer adequate 1:1 time with patients let alone CBT based interventions.’

‘Only feasible if enough staff are involved.’

‘All staff are asked to do unrealistic amounts of work, and this [guided self-help CBTv] may simply need too much time.’

This subtheme demonstrates the high workload that current mental health practitioners have and how this workload can prevent the dissemination of psychological interventions—which are arguably more time consuming than some alternatives (i.e. pharmacological treatments). The comments from clinicians suggest that this issue extends beyond the implementation of guided self-help CBTv, and instead reflects a tension within mental health services more broadly. It may be that reducing the number of therapy sessions (guided self-help CBTv uses half the sessions recommended for CBTp) is not sufficient to improve implementation.

2. *Conflict with Service Priorities*:

Beyond the issue of resources, some clinicians felt that the implementation of guided self-help CBTv was unlikely to be supported at a service-level e.g. by service leads and managers. It appeared that some felt an intervention like this conflicted with the priorities of services on two main fronts: firstly a conflict with the dominant treatment model, and secondly the need to meet targets:

‘I am dependant on managers who may be pressured to achieve targets and may not see interventions such as these as essential. It's pivotal to have leads and managers on board and offer protected time to learn and use these interventions.’

‘I think there will be resistance from practitioners who rely solely on the medical model.’

The comments within this subtheme may be partly explained by the responses given within the ‘patient barrier’ theme. Clinicians expressed concerns about the suitability of people who hear voices engaging in guided self-help CBTv because of their mental health and cognitive difficulties. The more senior members of the healthcare system may be influenced by these concerns and consequently show reluctance to support the implementation of this intervention.

‘It seems to me that as psychosis does not produce results or turnover suitable to corporate organisations it [treatment provision] will remain the poor relation within services.’

This theme suggests that it is not just the dominance of the medical model within mental health services that could prevent implementation, but also a need for services to meet targets. The implication of this statement is that those experiencing psychosis are a more complex client group that require more resources (and financial investment) to reach a service-defined point of recovery—this is not conducive with a target-driven mental health service. The pressure to meet targets to prevent any further funding cuts is likely to be exacerbated by the current state of mental health funding as described by the ‘Lack of Resources’ sub-theme.

### Study 2 discussion

The results from study 2 did not identify any facilitators to engagement. The barriers identified firstly addressed engagement (‘presenting problem’) and secondly implementation (‘practical barriers’). The ‘presenting problem’ theme found in study 2 mirrors the theme of the same name reported in study 1 –suggesting there is some consensus between people who hear voices and clinicians as to what the barriers to engagement with guided self-help CBTv could be.

## Overall discussion

Studies 1 and 2 sought to explore the views of people who hear voices and mental health clinicians respectively on their perceived facilitators and barriers to implementing and engaging with guided self-help CBTv. In study 1, people who hear voices reported that having a therapist who was compassionate, skilled and clear about confidentiality would facilitate therapy engagement. Whereas voices themselves and cognitive difficulties may act as a barrier. In study 2, mental health clinicians believed that the lifestyle, severity of symptoms and cognitive capabilities they associated with hearing voices would inhibit engagement. Furthermore a lack of resources, and support from service managers were identified as barriers to implementation.

Both clinicians and lived experience participants believed the presenting problem, including any associated cognitive impairments, could interfere with engagement. For the lived experience participants, the concern was primarily related to their voices, as voices may object to help-seeking and attempt to sabotage therapy engagement [24]. Whereas clinicians reported a broader concern about the mental state and lifestyle of the patient, and whether the intervention was only suitable for those deemed ‘stable’. The clinicians’ caution could reflect a greater scepticism as to the appropriateness of interventions that encourage patients to talk about their voices [25], or a pessimistic outlook as to the prognosis for people experiencing psychosis symptoms [9]. However the extraction of a similar theme from the lived experience participants could mean that symptoms are a ‘real’ barrier to engagement. However, our findings cannot clarify whether the attitudes from both groups are at all related (i.e. are clinicians projecting their opinions onto their patients, or are patients communicating their concerns to their clinician?) Regardless of the cause, this barrier needs consideration prior to and within therapy.

The somewhat negative attitudes of clinicians towards the efficacy of therapy for people distressed by hearing voices is further demonstrated by the continued dominance of the medical model, to the detriment of psychological services [26]. The clinicians reported this barrier to be especially prevalent when considering psychosis spectrum conditions. This point again reflects an underlying belief that clinically-defined recovery is not possible for most patients with psychosis [9]. While this attitude remains, commissioners are unlikely to invest in CBTp. However, in light of the findings from our lived experience participants, we must consider the possibility that engagement in therapy may be difficult for those experiencing psychosis symptoms. Before steps can be taken to address these negative attitudes, we must first identify whether they are justified within our broader research programme on guided self-help CBTv.

The qualities of the therapist were important to the lived experience participants. Some research suggests that some people who hear distressing voices frequently engage in negative patterns of relating to other people—typically being submissive and dependent [27]. It is perhaps unsurprising then that the participants were unanimous in their desire to have a therapeutic relationship that juxtaposes this negative relating. The ideal therapist described by the participants’ is akin with Roger’s [28] concepts of core conditions: (1) empathy, (2) genuineness (also described as congruence), and (3) unconditional positive regard. These conditions form the basis of the person-centred therapeutic approach, and their presence within therapy is associated with positive treatment outcomes [29]. However the correlational design of these studies is unable to determine whether the core conditions have any causal relationship with treatment outcomes [30]. Even if the core conditions do not directly cause beneficial outcomes, our findings suggest therapist qualities may have an indirect influence on treatment outcomes, by increasing intervention engagement [31].

Consistent with the CBTp literature more broadly [8], clinicians reported insufficient resources to be a barrier to implementation, despite the brevity of the intervention described. This highlights the current pressures upon mental health services [32], and, perhaps, that reducing the number of therapy sessions is not enough to improve implementation. Whether this concern is realised in practice remains to be seen. However if this is the case, then alternate approaches to the issue of access need to be considered. One approach could be to train frontline clinicians to deliver therapies, rather than accredited therapists. Initial findings for CBTp delivered by clinicians are promising [33]. However, adopting this approach would negate the response from our lived experience participants whom believed guided self-help CBTv should be delivered by therapists whom are both competent therapists *and* have specialist knowledge of working with people who hear voices. Also, at present, there are few controlled trials of CBTp delivered by frontline clinicians [10]. Until a body of high quality evidence is available, the delivery of CBTp by frontline practitioners in routine practice is not recommended.

### Limitations

The focus groups reported in study 1 were centred on the self-help book that we planned to base a guided self-help CBTv intervention around. The ‘Overcoming Distressing Voices’ [14] book is authored by two of the authors of this paper (see the conflicts of interest). Consequently, it is possible that our findings could be vulnerable to a positive researcher bias (i.e. a bias to present the self-help book in a positive light). To address this, and in the spirit of transparency, the research team agreed to publish the results of this research irrespective of the results. Also the full analysis of the qualitative data (see the supplementary material) includes themes that highlight some of the perceived flaws of the self-help book and the intervention idea. We hope that these results can demonstrate researcher bias is not a significant limitation of this study. Moreover, we would like to acknowledge there are other self-help books available that use CBT principles to support people experiencing the symptoms of psychosis e.g. [34,35].

Studies 1 and 2 utilised different methods to consult with participants, each with their own limitations. For example, study 2 used free response items on a questionnaire to consult clinicians, and probes were not available to explore participants’ responses. A more traditional qualitative design could have been used (e.g. interviews) to collect a richer level of data. However, on reflection, using a questionnaire design meant that we could consult a larger, more diverse, and consequently a more representative group of clinicians. In contrast, study 1 may have limited generalisability because of the largely self-selected recruitment method used; especially when considering the representativeness of our participants’ literacy levels, and type of voices they experience. Firstly, there is some evidence to suggest people who hear voices have generally lower levels of educational attainment [36]. However there is no significant difference between the reading ability of voice hearers and nonclinical controls [37]. Consequently, because a required pre-requisite for guided self-help CBTv is the ability to read and write it is likely that our sample represents the intervention’s target client base. Secondly, there are numerous subtypes of voices, such as (1) constantly commenting and commanding, (2) replay, and (3) own thought voices [38]. As we did not ask participants what types of voices they heard, we cannot say whether the themes derived would be applicable to all types of voices-hearers. However, we do know that most people who hear distressing voices (this would include all of our participants) typically hear more than one type of voice [39]; so it is likely that the majority of these voice subtypes were represented within our sample.

Moreover this study recruited both clinicians and people who hear voices from one mental health trust in the UK. The experience of, and attitudes towards, voices can differ significantly across cultures [40]. Also, a number of the themes described are related to the health service the participants were a part of (e.g. conflict with service priorities). Therefore the themes extracted may not be generalizable to other mental health care services or trusts, both nationally and internationally. Consequently, the solutions we propose to increasing access (i.e. reducing the duration of therapy) may not be appropriate for all mental health services. Despite this, it is probable that some of the themes extracted will apply to other geographical localities. For example, CBTp dissemination is poor both nationally and internationally due in part to problems with resource [41–43]. It is therefore likely that many mental health clinicians outside of this Trust would also report a lack of resources as a barrier to implementation.

The participants from both studies were asked to consult on the guided self-help CBTv intervention without having received or delivered the intervention. Therefore the responses presented here will be restricted by the extent to which they are grounded in relevant experience. However conducting the consultations described in studies 1 and 2 at this point in the development process of guided self-help CBTv means we can incorporate the feedback more easily into the intervention protocol, and consider how to address the barriers prior to implementation. Once people who hear voices and mental health clinicians experience this intervention their perceptions may change. We plan to use a mixed-methods approach (both quantitative and qualitative) when evaluating the effects of guided self-help CBTv to continue learning about the barriers and facilitators to engagement and implementation.

### Clinical applications

The most pertinent clinical application that can be taken from these results is the impact of the shared perception (both clinicians and lived experience) that the presenting problem could be a barrier to guided self-help CBTv. From the clinicians’ perspective, this belief may determine who is offered psychological therapy—whereby therapy is not offered to those with the most severe symptoms. And where therapy is offered, clinicians’ negative attitudes towards their patient’s potential to benefit from the intervention may directly hamper the treatment outcomes [44]. Clinicians should endeavour to remain hopeful for their patients to promote recovery and motivation [45,46]. The implementation of any psychological therapy should include a programme of work to raise awareness amongst referring clinicians to prevent the unwarranted gate-keeping of patients.

From the lived experience perspective, the concern about the sabotaging role of voices could be responded to both prior to and during therapy. Prior to therapy, the therapist could explicitly recognise the possible responses voices may have to therapy, how this relates to the voices’ negative intentions over the hearer (e.g. the voice does not want the hearer to get stronger), and how this can be managed. During therapy, sessions can be used to explicitly evaluate beliefs about the voice’s omnipotence (e.g. how therapy engagement can challenge the voices power) [47] and of self-efficacy (e.g. how therapy engagement can evidence taking control over from the voice) [48]—both common elements of CBT for voices [14,49].

Beyond the content of therapy, the potential for impaired cognitive abilities to hinder therapy engagement (as reported by both lived experience participants and clinicians) has specific implications for this guided self-help CBTv intervention that uses written materials. These materials need to make use of different formats (e.g. images and text), and cater to varying levels of cognitive ability (e.g. long and short passages). These materials are likely to be most helpful if supplemented with face-to-face contact that collaboratively engages with and works through these materials. The quality of this face-to-face contact was a concern for the lived experience participants who wanted a therapist who was compassionate, empathetic, and trustworthy. These qualities predict a better therapeutic relationship [50], which in turn moderates treatment outcomes [51]. Thought will need to be given as to how such therapist qualities can be foregrounded within a brief intervention that allows only a limited time to develop the therapeutic relationship.

### Conclusions

The findings from both study 1 and 2 demonstrate that both clinicians and people who hear voices anticipate a number of barriers and facilitators to the implementation of guided self-help CBTv. Identifying these barriers, from each perspective, will enable all parties to openly and collaboratively consider the possible solutions. Whether these barriers are realised in practice, requires further research.

## Supporting information

S1 File Staff survey. (DOCX)

S2 File Discussion guide. (DOCX)

S3 File Complete analysis. (DOCX)

S4 File Complete data. (DOCX)
