# Supplementary material for: Barriers to disseminating brief CBT for voices from a lived experience and clinician perspective
Source: PLoS One. 2017 Jun 2;12(6):e0178715. doi: 10.1371/journal.pone.0178715 (PMC5456317; doi:10.1371/journal.pone.0178715)
Supplement: S1 File — (DOCX) [file pone.0178715.s001.docx]

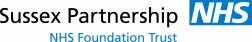


**Staff Survey:**

Attitudes Towards Guided Self-Help CBT for Distressing Voices.


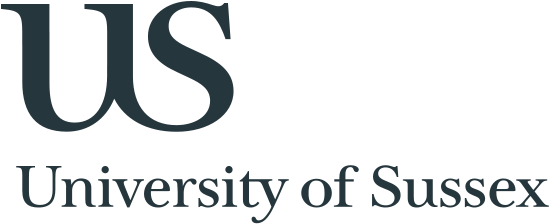

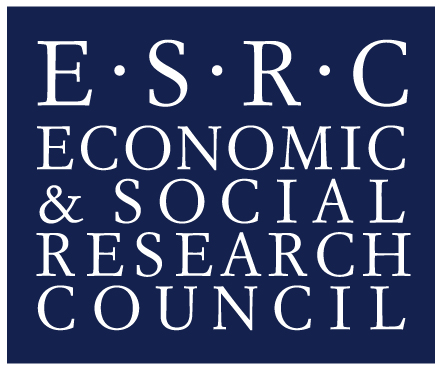


In partnership with:

**Participant Information Sheet:**

Thank you for your interest in this survey; it should take no longer than 10 to 15 minutes to complete.

**Background:**

For people with common mental health problems, like anxiety and depression, treatment options include low intensity therapies. These are interventions that are based on evidence-based therapies, like Cognitive Behavioural Therapy (CBT), but are delivered in a way that reduces the amount of time and resources involved.

We plan to trial a low intensity intervention for people who hear distressing voices (verbal auditory hallucinations). The specific intervention we are planning to trial is guided self-help CBT for those that hear distressing voices. Clients would work with a non-therapist that has had specific training, to work through a self-help CBT book specific to distressing voices.

**1. What is the purpose of the study?**

Staff are an important part of taking any new idea and helping to successfully implement it. If this self-help intervention is to be integrated into the NHS then it is important to understand what staff think about it. We would therefore be grateful if you could take the time to complete this survey.

**2. Who can take part?**

- All clinicians (e.g. mental health nurse, support worker, occupational therapist, clinical psychologist) working in secondary care adults mental health services (e.g. AOT, ATS etc.)
- All clinicians working in primary care mental health services; both step 2 and 3 professionals (e.g. Health in Mind and Well Being Services)
- All clinicians (e.g. mental health nurse, support worker, occupational therapist, clinical psychologist) working in Early Intervention Services for psychosis

**3. Do I have to take part?**

You do not have to take part. Your consent to be involved in the study is implied by handing in your completed survey to the research team.

**4. What do I have to do?**

If you decide to take part, then you should proceed with the questionnaire. They are mostly multiple choice, with a few free text boxes if you wish to elaborate on any of your answers.

You should read each of the statements carefully and choose the answer that best suits your opinion. The survey will take approximately 10 to 15 minutes to complete.

**5. What are the draw backs to taking part?**

The survey will require you to spend some of your own time to complete it. The survey is not very long and estimated to only take 10 to 15 minutes to complete, so hopefully will not be of too much an inconvenience. There are perceived to be no other drawbacks to taking part.

**6. What are the benefits of taking part?**

You will have a chance to be involved in shaping the self-help intervention. Your opinion counts, and will be a key part in the development of future research studies.

**7. Will my taking part be kept confidential?**

Yes. No identifiable information will be collected within the survey. All your answers will be anonymous. We will not ask for your contact information, and your will not be followed up by any of the research team regarding your responses. Any data collected will only be seen by the members of the research team.

**8. What will happen to the results of the study?**

The results will reported within a research article that we hope to publish within a mental health research journal. The article will also be included within a PhD thesis at the University of Sussex.

**9. Who is organising this research?**

This research is being co-organised by the University of Sussex and Sussex Partnership NHS Foundation Trust, and is part funded by the ESRC.

**10. Who has reviewed this study?**

This study has been approved by the ethics committees at the University of Sussex, through the Sciences and Technology Cross-School Research Ethics Committee (CREC; application number: ER/CH283/4). This ethics committee can be contacted via email, using the following address: and give the CREC name and email address: [crecscitec@sussex.ac.uk](mailto:crecscitec@sussex.ac.uk). The research has also been approved by the Sussex Partnership NHS Foundation Trust Research and Development department.

**11. Who do I contact if I want to know more about the study?**

Please direct any questions to Miss Cassie Hazell via email: [ch283@sussex.ac.uk](mailto:ch283@sussex.ac.uk)

**About You:**

**1. Age:**

……………………………………………………

**2. Gender:**

Male ☐

Female ☐

Prefer not to say ☐

**3. What team are you part of within mental health services?**

Primary care ☐

Assessment and treatment ☐

Recovery and wellbeing ☐

Inpatient ☐

Early intervention ☐

Assertive outreach ☐

Other *(please specify below):* ☐

…………………………………………………………………………………………

**4. What is your profession?**

Psychological wellbeing practitioner (Step 2 practitioner) ☐

Nursing ☐

Social work ☐

Occupational therapy ☐

Support worker ☐

Clinical/counselling psychologist ☐

CBT therapist ☐

Counsellor or other psychotherapist ☐

Other *(please specify below):* ☐

…………………………………………………………………………………………

**5. How long have you been in this profession?** *(Please give your answer in years)*

……………………………………………………

**6. Within your work in mental health services, how much experience do you have working with people who are distressed by hearing voices?**

A lot (worked with 10+ people distressed by hearing voices) ☐

Some (worked with 5-9 people distressed by hearing voices) ☐

A little (worked with 1-4 people distressed by hearing voices) ☐

None (no experience with this group) ☐

**7. How much cognitive behavioural therapy (CBT) training have you received?**

Qualified CBT therapist or equivalent ☐

Qualified psychological wellbeing practitioner or equivalent ☐

In training as a CBT therapist or equivalent ☐

In training as a psychological wellbeing practitioner or equivalent ☐

10 or more days of CBT training ☐

5 to 9 days of CBT training ☐

1 to 4 days of CBT training ☐

No formal CBT training but use ideas in my work ☐

No formal CBT training and do not use CBT ideas in my work ☐

Below are a range of statements aimed to understand your opinion on the concept and implementation of guided self-help CBT for distressing voices.

Please read each statement carefully and give your honest answer. Please try to answer all the questions below.

**Section One:**

8. Select the answer that best fits your opinion for each statement.

| 1 | 2 | 3 | 4 | 5 | 6 | 7 |
| --- | --- | --- | --- | --- | --- | --- |
| Strongly Agree | Agree | Somewhat Agree | Neither Agree nor Disagree | Somewhat Disagree | Disagree | Strongly Disagree |

| A | Randomized controlled trials e.g. comparing the treatment to a control group, is a good way to evaluate the effectiveness of guided self-help CBT for distressing voices | 1 | 2 | 3 | 4 | 5 | 6 | 7 |
| --- | --- | --- | --- | --- | --- | --- | --- | --- |
| B | Guided self help CBT for distressing voices is an appropriate treatment option | 1 | 2 | 3 | 4 | 5 | 6 | 7 |
| C | Qualitative measures e.g. interviews after the intervention, are a good way to evaluate the effectiveness of guided self help CBT for distressing voices | 1 | 2 | 3 | 4 | 5 | 6 | 7 |
| D | Guided self help CBT for distressing voices would be effective for those with long standing symptoms | 1 | 2 | 3 | 4 | 5 | 6 | 7 |
| E | I would be willing to be involved in the development of guided self help CBT for those with distressing voices | 1 | 2 | 3 | 4 | 5 | 6 | 7 |
| F | I would be willing to refer a client who hears distressing voices to receive guided self help CBT as part of a research project | 1 | 2 | 3 | 4 | 5 | 6 | 7 |
| G | I would be happy to refer a client who hears distressing voices to receive guided self help CBT | 1 | 2 | 3 | 4 | 5 | 6 | 7 |
| H | It is a waste of resources to trial guided self help CBT for those who hear distressing voices | 1 | 2 | 3 | 4 | 5 | 6 | 7 |
| I | I would be willing to be involved in research that is trialing guided self help CBT for distressing voices | 1 | 2 | 3 | 4 | 5 | 6 | 7 |
| J | Attending a separate supervision for those who deliver guided self help CBT for stressing voices would not be practical for me | 1 | 2 | 3 | 4 | 5 | 6 | 7 |
| K | Self help materials e.g. books, would be effective for those that hear distressing voices | 1 | 2 | 3 | 4 | 5 | 6 | 7 |
| L | People who hear distressing voices would not be able to engage in guided self help CBT | 1 | 2 | 3 | 4 | 5 | 6 | 7 |
| M | It is not possible to implement guided self help CBT, within existing mental health services | 1 | 2 | 3 | 4 | 5 | 6 | 7 |
| N | It would be possible to free up time to deliver guided self help CBT for distressing voices | 1 | 2 | 3 | 4 | 5 | 6 | 7 |
| O | It would be possible to find the time to attend a two day training course on how to deliver guided self help CBT for distressing voices | 1 | 2 | 3 | 4 | 5 | 6 | 7 |
| P | Majority of my clients who hear distressing voices would like to receive guided self help CBT | 1 | 2 | 3 | 4 | 5 | 6 | 7 |
| Q | I would not be prepared to receive training to deliver guided self help CBT for distressing voices | 1 | 2 | 3 | 4 | 5 | 6 | 7 |
| R | I would be willing to deliver guided self help CBT for distressing voices as part of my job | 1 | 2 | 3 | 4 | 5 | 6 | 7 |
| S | Measures of other clinical symptoms e.g. anxiety and depression, are a good way to evaluate the effectiveness of guided self help CBT for distressing voices | 1 | 2 | 3 | 4 | 5 | 6 | 7 |
| T | Measures of symptom severity e.g. psychosis measures, are a good way to evaluate the effectiveness of guided self help CBT for distressing voices | 1 | 2 | 3 | 4 | 5 | 6 | 7 |

**Section Two:**

9. Select the answer that best fits your opinion for each statement.

| 1 | 2 | 3 | 4 | 5 | 6 | 7 |
| --- | --- | --- | --- | --- | --- | --- |
| Strongly Agree | Agree | Somewhat Agree | Neither Agree nor Disagree | Somewhat Disagree | Disagree | Strongly Disagree |

| A | Those who hear distressing voices should be able to receive the same treatment options as those with common mental health problems | 1 | 2 | 3 | 4 | 5 | 6 | 7 |
| --- | --- | --- | --- | --- | --- | --- | --- | --- |
| B | CBT can be effective for those who hear distressing voices if it is delivered by a non-therapist e.g. psychological wellbeing practitioner | 1 | 2 | 3 | 4 | 5 | 6 | 7 |
| C | I believe my colleagues would be willing to be involved in trials for guided self-help CBT for distressing voices | 1 | 2 | 3 | 4 | 5 | 6 | 7 |
| D | It would be possible to identify clients that are suitable to receive guided self-help CBT for distressing voices | 1 | 2 | 3 | 4 | 5 | 6 | 7 |
| E | Measures of quality of life e.g. engagement in meaningful activity, are a good way to evaluate the effectiveness of guided self-help CBT for distressing voices | 1 | 2 | 3 | 4 | 5 | 6 | 7 |
| F | Research is a good method of testing a new intervention | 1 | 2 | 3 | 4 | 5 | 6 | 7 |
| G | The resources needed to trial guided self-help CBT for distressing voices are available | 1 | 2 | 3 | 4 | 5 | 6 | 7 |
| H | Having a client who hears distressing voices receiving guided self-help CBT would make my workload unmanageable | 1 | 2 | 3 | 4 | 5 | 6 | 7 |
| I | My team would be able to aid in the implementation of guided self-help CBT for distressing voices | 1 | 2 | 3 | 4 | 5 | 6 | 7 |
| J | I would be willing to attend a course on the therapeutic principles of guided self-help CBT for those who hear distressing voices | 1 | 2 | 3 | 4 | 5 | 6 | 7 |
| K | Measures of acceptability e.g. client satisfaction, is a good way to evaluate the effectiveness of guided self-help CBT for distressing voices | 1 | 2 | 3 | 4 | 5 | 6 | 7 |
| L | Being trained to deliver guided self-help CBT for distressing voices would make my job harder | 1 | 2 | 3 | 4 | 5 | 6 | 7 |
| M | Guided self-help CBT for distressing voices sounds like a good idea | 1 | 2 | 3 | 4 | 5 | 6 | 7 |
| N | Guided self-help CBT for those who hear distressing voices would be unsafe | 1 | 2 | 3 | 4 | 5 | 6 | 7 |
| O | Symptom specific treatment, like guided self-help CBT for distressing voices, are a good approach to treatment | 1 | 2 | 3 | 4 | 5 | 6 | 7 |
| P | Having 6-8 sessions of guided self-help CBT for those with distressing voices would be feasible to implement as a treatment option | 1 | 2 | 3 | 4 | 5 | 6 | 7 |
| Q | Following clients up after a period of several months to administer clinical measures is a good way to evaluate the effectiveness of guided self-help CBT for distressing voices | 1 | 2 | 3 | 4 | 5 | 6 | 7 |
| R | I would be willing to have training to be able to deliver guided self-help CBT for distressing voices | 1 | 2 | 3 | 4 | 5 | 6 | 7 |
| S | Guided self-help CBT for those who hear distressing voices will be very effective | 1 | 2 | 3 | 4 | 5 | 6 | 7 |
| T | Measures of the distress experience from hearing voices is a good way to evaluate the effectiveness of guided self-help CBT for distressing voices | 1 | 2 | 3 | 4 | 5 | 6 | 7 |

**Section Three:**

10. What do you think about the idea of offering CBT for distressing voices using guided self-help? *(Optional)*

11. How willing would you be to be involved in the development of guided self-help CBT for distressing voices? *(Optional)*

12. How feasible do you think it would be to implement guided self-help CBT for distressing voices in the trust? *(Optional)*

13. How should guided self-help CBT for distressing voices be evaluated? *(Optional)*

Thank you…

Thank you for completing this survey.

Your opinions will be used to improve future research.

The findings from this survey will be disseminated once the write up is complete.

If you have any questions please direct them to Miss Cassie Hazell, using the email address [ch283@sussex.ac.uk](mailto:ch283@sussex.ac.uk)
