# Supplementary material for: Barriers to disseminating brief CBT for voices from a lived experience and clinician perspective
Source: PLoS One. 2017 Jun 2;12(6):e0178715. doi: 10.1371/journal.pone.0178715 (PMC5456317; doi:10.1371/journal.pone.0178715)
Supplement: S2 File — (DOCX) [file pone.0178715.s002.docx]

Focus Group – Discussion Guide.

Introductions: (5 minutes)

- Introductions: Name and role
- Purpose: To get feedback from the group on the idea of guided self-help CBT for distressing voices
- There is no right or wrong answer
- It is ok to have a different opinion to someone else in the group
- Participants are reminded to try not to interrupt or speak over each other
- At times the researcher may put forward questions or topics of interest in order to keep the conversation focussed on the research questions

Data Collection: (5 minutes)

- Consent forms
- Demographic information
- Any questions before the discussion begins
- Beginning of the audio recording

Warm-Up Discussion: (20 minutes)

- Discussion on guided self-help CBT
- Understanding what distressing voices are
- General discussion of the self-help book
  - What does everyone understand by the term guided self help?
  - What did you think of the book? What did you like? What didn’t you like?

Subsequent Discussion: (45 minutes)

***Participants will be given a template of a proposed session structure for the intervention and will be asked to comment on this***

- Programme factor
- Probing for:
  - Number of sessions
    - What do you think about 8 sessions for this intervention?
  - Session length
    - How long do you think each session should be?
  - Thoughts on the material
    - What do you think about the content of each session?
  - Structure
    - What do you think of the proposed structure?
  - Order
    - What order should the sessions be in?
- Problem factor
- Probing for:
  - Cognitive problems e.g. memory
    - Can you think of any issues that might make it difficult to engage in the intervention? If so, how could we help with this?
  - Symptom severity
    - Is there anything related to the voices that might make it difficult to take part?
  - Reaction of voices to intervention
    - How do you think voices might react to this intervention?
- People factor
- Probing for:
  - Demographic variables
    - Who do you think this intervention would be helpful for?
  - Client expectations
    - If you were to receive this intervention, what would you expect the outcomes to be?
  - Types of voices being experienced
    - Are there any kinds of voices you think this intervention would/would not be helpful for?
- Provider factors
- Probing for:
  - Where it should be held
    - Where would you like to meet the therapist for this intervention?
  - The support needed
    - How much contact would you want from the therapist?
    - How would you like to receive support?
    - Who do you think would make a good coach?
  - Additional means of contact
    - Would you like to be contacted in-between sessions?
    - If so, how would you like to be contacted?

Advice to the researcher (5 minutes)

Advice to the therapist (5 minutes)

Plenary:

- Next steps of the research programme
- Final questions
- Giving thanks to the participants
