# Supplementary material for: Barriers to disseminating brief CBT for voices from a lived experience and clinician perspective
Source: PLoS One. 2017 Jun 2;12(6):e0178715. doi: 10.1371/journal.pone.0178715 (PMC5456317; doi:10.1371/journal.pone.0178715)
Supplement: S3 File — (DOCX) [file pone.0178715.s003.docx]

**Ideas are easy, implementation is hard: Facilitators and barriers to disseminating a guided self-help CBT intervention for distressing voices from a lived experience and clinician perspective.**

# Supplementary Material.

# Study 1 (lived experience):

## The Self-Help Book:

It is perhaps unsurprising that as participants were given a copy of the ‘Overcoming Distressing Voices’ self-help book (Hayward, Strauss & Kingdon, 2012) that will form the basis of this intervention, that one of the themes extracted identifies the participants opinions on the book. All of the participants had read at least one chapter from the book before attending the focus group.

### Positive feedback

Most participants provided some positive feedback about the self-help book. One of the most prominent strengths of the book was the use of case studies to illustrate theories. Many of the participants felt that the case studies closely echoed their own experience of voices.

Jillian: ‘Yeah referring back to this [the book], um I think that I can relate to all of what’s in here um which is quite amazing reading it.’

Bobby: ‘It is really sort of um, it characterised my schizophrenia really down to a tee. And comments in there by Ruth and Martin [characters in the book] and their own personal experiences so I can relate to it. I haven’t actually read it all. I read it a couple of weeks ago. Um, no it’s good. It’s quite therapeutic.’

Being able to relate to the book helped both Jillian and Bobby to feel less alone. Both commented on how surprised they were to find such strong similarities between their experiences and that described in the book. The perceived ‘realness’ of the case studies appeared to increase confidence in the credibility of the book, and the advice it suggested. In addition to the case studies, the language used within the book encouraged the participants to read the book. The language used was perceived as accessible and easy to understand.

Sam: ‘Yeah it was easy for me to read. I thought the language, and it was quite compassionately written I think, quite kindly written.’

Jonathan: ‘I found while reading it. It’s just very easy to read. And it’s just, and it’s accessible. Some of the things like tables and diagrams I though whoa I won’t be able to grasp it well and it was easy as well suppose. It’s easy to follow.’

Jonathan talks about feelings apprehensive about reading the book after scanning through the pages. The use of diagrams and tables he felt were initially off-putting but this apprehension soon dissipated after he began reading. Jonathan’s ability to understand the book seemed to give him a sense of achievement, and could (regardless of the books content) have a therapeutic effect.

### Negative feedback

Although the feedback from participants was largely positive, there were a number of critical comments offered. Some of these ideas are in direct conflict with the positive comments discussed above.

Jonathan: ‘I struggle to in bring my case into the literature. So I would really have to think and think and think so it’s not as straight forward for me really. It’s helped me to identify only to a certain degree really. So, yeah.’

Tim: ‘It was very different from the experiences given. Um I couldn't, yeah. I could relate to them as separate people. But I couldn't fit my own experience into that category, into that category.’

Hearing voices and mental health more generally is a personal experience that can be influenced by many individual differences. The diverse nature of hearing voices meant that both Tim and Jonathan felt that the case studies did not present their experience. Arguably it is not possible to produce a brief and accessible case study that everyone who hear voices could relate to. However being able to have a personal connection with the self-help book seemed to be important to the participants. Consequently we must consider other means of personalising the self-help book that doesn't use case studies.

Related to this issue, is the criticism from some participants that the book (including the case studies) was too simplistic. In addition to being a personal experience, hearing voices can also be complex.

Joe: ‘It takes a very basic level of um sort of voice hearing and it can be a lot more complex than that. I think the book, I think it’s quite hard for any book to get one person’s sort of view on voice hearing, their experiences.’

This criticism is at odds with the positive comments that praised the self-help book for being easy to understand. Increasing the book’s complexity may help some people to identify with and relate to the material. However this is likely to exclude those that are unable to comprehend information at that level of complexity. The difference in opinion between these sub-themes shows how difficult it is to produce self-help materials that are suitable for everyone.

### Self-reflection

Reading the self-help book prompted some of the participants to review their own experience of hearing voices in light of what they were reading.

Sam: ‘I think its gentle enough. I think it’s, like, it’s not difficult reading, it’s not frightening reading. Well I didn't think. You know facing other people, seeing that other people go through similar experiences is very enlightening it makes you feel part of something – you’re not on your own.’

Jonathan: ‘I think the way it worked for me I started having some sort of intuitions, the voices saying this and that and it expanded, and it confirmed my suspicions. I have been in the services for quite a long while and not being able to talk really about voices and the book confirms my intuitions and that was the greatest thing really.’

Pippa: ‘Yeah because mine is an outrage really of my inner creators and invasion of privacy, although it’s all happening in my mind; and I have to say that other times I feel differently. So when I looked at the table [in the book] I was really able to say ok that's my situation and once you know what your situation is it’s easier to address.’

The participants within this study were not instructed to try to treat themselves using the book – they were instructed to read the book with the view to consulting on a new intervention idea. Despite this, it seems that some of the participants engaged in some self-reflection as a result of reading the self-help book. This kind of self-evaluation is one of the key skills needed within CBT in order to identify and challenge unhelpful beliefs. It is promising of the future of this therapy that a number of the participants engaged in this process spontaneously.

## Therapy Protocol:

In addition to reviewing the self-help book, the participant were asked to consider how it could be used within a guided self-help therapy. The participants picked out a number of topics that they felt were important should be discussed within the intervention. Each subtheme discussed here describes a topic that the participants identified.

### Self

Although the self-help book has a primary aim of reducing the distress associated with hearing voices, participants talked about the self-esteem chapters of the book most often. Considering beliefs about the self and self-esteem was seen as important by all participants. The participants across all three focus groups agreed that the self should be discussed first within the intervention.

Tim: ‘Self-esteem is important because if your self-esteem is really low then you’re less likely to be able to challenge your voices because um I think you give them more power. You see them as bigger than yourself um and you won’t kind of be able to have enough self-esteem to even want to challenge them, you just go along with letting them beat you around.’

Nikki: ‘We can have a day where we do 90% productive, a good day where we have just gone about our business and it’s all been fine, but the 10% that I do that I feel like I can criticise myself for I will. So I will forget about the 90% that happened that actually I was loving and kind towards people and I was good with my family or whatever. I will forget up about that and I will beat myself up about the 10% all the time I will go no but you did that bit sh*t just that little bit of the day and I think challenging that is really interesting and the core belief stuff is really interesting.’

The quote from Jimmy below creates a hierarchical image of recovery. This hierarchy priorities the self before voices. He felt the need to create a strong core from which to challenge the voices that were identified as a product of the self. Jimmy’s response suggests that it would not be possible to reduce the distress associated with voices until he has addressed and unhelpful beliefs about the self.

Jimmy: ‘I agree that um beliefs about self should come first because that gives a sort of basis for everything else so um you can’t um sort of um, when we are talking about voices you are obviously talking about the way you are reacting to those voices and so yeah it so you have got to talk about yourself first.’

### Voices

None of the participants discussed their voices in a positive light – all participants attributed negative emotions to the experience of hearing voices. Many of the participants appeared to understand the aim of CBTv is to reduce the distress associated with voices, and not reduce voice frequency. Many participants understood how this change could result in positive outcomes.

Jimmy: ‘I think the challenging beliefs in the power that the voices have. You know when they are really loud and when they are really insistent I think that what they are saying is true and um and that they can make me do things that if I could challenge that in that moment then that would be you know that would completing change everything really.’

Jonathan: ‘I think the voices themselves are not as bad as the thing they can do with you in terms of how you respond. I mean it could be self-neglect or some other things. I always think that if I only heard the voices then it would fine but it's the other problems like how I respond to them.’

In contrast to some participants views that the self-help book could be too narrow and simplistic, some of the participants liked the symptom-specific approach. For those participants who reported cognitive difficulties (see below), having a specific therapy aim helped to minimise distraction and increase transparency. Participants liked the idea of knowing what they would be asked to talk about within the intervention, and what aspects of their mental health they were trying to improve. Within a briefer form of therapy, like guided self-help CBTv, taking a symptom specific approach can ensure that the limited time is used most efficiently.

Tim: ‘[Therapy] felt a lot more useful if I know I’m there just to focus on a specific topic around voice hearing and not all the other things that might be going on for me.’

Jimmy: ‘As Tim says it has just such a huge impact on your life because it’s there all the time, and um yeah so I think it's [symptom specific approach] a really good idea.’

### Relationships

The self-help book looks at both the voices and the self within both a traditional CBT and relational framework. The relating approach was a new concept to most of the participants. Some of the participants agreed with the concept if a voice-hearer relationship. Even if it was not mentioned explicitly, many of the participants discussed their voices using relational language.

Pippa: ‘This [the book] helps you, well it helped me, to realise that it takes a little work to regain your faith in other people and then in you. It’s all down to the way you reply to them [voices], even verbally, so if you improve your relationship with your voices you will then improve your relationship with the outside world.’

Ed: ‘Well one thing positive one thing [about the book] is having a relationship with them [voices] because it is a relationship. It’s not going to suddenly go away so the whole denying it and stuff... so I think having a relationship with them, and maybe not talking to them, but giving them as you were saying some leeway like an actual amount of time for them to do what they want.’

Ed in particular connected with the idea of having a relationship with his voices. Having this relational understanding resulted in the development of coping strategies that respected the voice as an equal member of the relationship. His response above illustrates one way that Ed manages his voices – by giving the voices an allotted amount of time to take control has helped him to negotiate some protected time that he can have for himself without the voices.

### Coping strategies

Coping strategies was the final topic from the self-help book identified by the participants. Finding ways to cope with voices was prioritised by many of the participants. The participants discussed the coping strategies they have found helpful, and how these relate to the ones suggested within the book.

Sam: ‘It’s just finding as many coping mechanisms as you possibly can, um which I think should be more detailed in here [the book], um because that's what we need you know.’

Catherine: ‘I mean there were times when I didn't turn it around. I have learned coping mechanisms. I will say ‘oh yes I can’ which has given me a bit of strength.’

Catherine’s response illustrates that developing helpful coping strategies is a process of learning and experimentation. Where participants talked about coping strategies, they were discussed in relation to a trial-and-error process, whereby different strategies were tried and evaluated until helpful ones were found. Also having one coping strategy was considered insufficient – instead it was important to have as many as possible for different situations and voices.

## The Therapist:

### Personal qualities

See main paper.

### Therapist skills

See main paper.

### Confidentiality in therapy

See main paper.

## Pragmatics of Therapy:

With regard to this theme, more so than any others discussed thus far, there was little consensus amongst the participants. This theme covers all aspects of the intervention that does not relate to the therapy content.

### Therapy structure

Some of the participants wanted to have some contact with their therapist between therapy sessions.

Jillian: ‘I have just had an occupational therapist um and she used to um give me a text just to ask me how I am and for me to let her know that I am ok. So I think that's a really good thing.’

Lee: ‘To have the option to be able to phone someone up. Just to have that option that you can phone this number and they will get back to you within a day or two if you want to discuss something specifically or you’re really mixed up about something.’

Whereas other participants felt that contact in-between sessions would be unhelpful.

Tim: ‘I find that [a phone call] a little bit intrusive. Because who knows where I will be when that call comes um and I might feel a bit like I’m being chased up and I have to a think of a response, whereas if it's a text message it’s up to me what I do with that.’

Sam: ‘I would be on the phone nattering away to my therapist for no good reason it would be a waste of their time I just want it to be completely focussed for that one hour a week... the boundaries of the relationship become crossed and you’re like ‘oh why am I ringing up my therapist?’ Oh because I want a chat and a cup of tea.’

Again, when asked whether they would prefer guided self-help CBTv to be delivered on a one-to-one basis or as part of a group, some of the participants expressed a preference for individual therapy.

Jimmy: ‘Because you are like talking about your own beliefs in yourself and your own voices, because it is a very personal therapy, personal subjects, I think it would work better on a one to one basis um because obviously everybody in the group is going to have a very different experiences and beliefs. And um I don't know if you would sort of maybe waste time sort of listening to, sounds awful wasting time, but you would spend a lot of time listening to the individual differences and you wouldn't have the time to focus on how you’re going to challenge, or what your own beliefs are in fact, so yeah it would work better on your own.’

Jackie: ‘I would prefer to do it one to one because then you can talk more. People won’t pressure you to talk about things that you don't want to talk about.’

Nikki: ‘I think there’s some things that are very private that you wouldn't necessarily say in a group, so I wouldn't think it would get to the core issues for me because I wouldn't share it in a group. I think privately would help.’

In contrast, some participants valued the social aspect of a group-based intervention.

Joe: “For you to get the most out of it, you know in groups, it’s good because you get other people experiences and you hear about it.”

The differences in opinions reported here show how difficult it can be to design an intervention that is standardised and suitable for all. It is important for research quality and implementation that interventions like guided self-help CBTv are guided by a structured protocol. However to maximise patient benefit and experience, there needs to be a degree of flexibility to accommodate these differences in opinion, wherever it is possible.

### Timing

The participants discussed when they felt would be a good time to engage in guided self-help CBTv. For some of the participants this sense of ‘readiness’ was discussed as following a linear trend – comparing those who have just started hearing voices, to those who have heard them for a number of years. Other participants believed that feeling ready to engage in the intervention followed more of a fluctuating trajectory that was influenced by how ‘well’ they felt.

Tim: “You need to have a certain level of wellness in order to engage with the book.”

Joe: “I know that when I have been ill there is a lot more going on and I think it really depends on how, where you are in your illness. I know that if I had read this book a few years ago, I wouldn't be able to use the stuff. It wouldn't really compute with you or anything like that. I wouldn't be able to sort of um, use it as a self-help book. But obviously further down the line its better, easier to use.”

The participants largely agreed that trying to engage in guided self-help CBTv while unwell would not be appropriate or helpful. Being in a more ‘stable’ state seemed to be important in deciding a good time to intervene. It is important to note that the participants did not feel that intervening at a time of mental ill-health would be unsafe. As described by Joe, intervening at that time would just be unlikely to produce any benefit.

There was some disagreement when considering whether guided self-help CBTv would work as part of an early intervention care package. Some participants felt that the intervention could help to address the initial feelings of confusion when a person starts hearing voices by increasing their understanding.

Sam: ‘I mean these sorts of books [self-help book] should be available in schools as well because like if a kid starts hearing voices, that’s when I started hearing voices as a kid, I thought I didn't even know that was something that could happen to anyone. I didn't even know that even existed. I didn't even realise that was a thing so you know.’

Mia: I think for me, something like that would have been good when it started, when I started to hear voices and to feel down, because I didn't understand what it all was and when I started hearing voices there wasn't any um sort of real help. So I think something like that would be quite good, if you could catch someone who just like an early sort of intervention.’

Jeremy: It’s [the book] nice because you can pick out so many different coping mechanisms and strategies and stuff that I wouldn't personally think about myself, so that's why I think that book would be good for what I call ‘starter outers’.’

In contrast other participants felt that it would be important to have an understanding of you voices before you can engage in an intervention like guided self-help CBT. This understanding is something that comes with time.

Jimmy: “It’s [the voices] all too confusing early on and there’s all too much going on and you don't really, you’re not stable enough um to um properly think about or talk about your voices. From my experience, I didn't want to talk about it because I was really embarrassed about it and to be forced into that position it would be quite damaging I think, um so I think early on you are definitely, it could either be a waste of time or it could be damaging I would say.”

Joe: “Helping people early on with it would be helpful but I think the whole process of trying to challenge everything would be too difficult. Well I know it was when I was that ill and stuff.”

Pippa: “You know I think some therapy is important when voices first start, but because they start with derogatory and insulting content, and you have to give somebody who is just experiencing this for a brief period of time, a therapy about themselves and how they see themselves it might appear to them either a little off topic or that your blaming them and agreeing with the voices about them being weak you know um and vulnerable... if I was in a therapy where I was told to challenge core beliefs right at the very beginning it might be too difficult.”

Starting to hear voices was frequently associated with a sense of chaos and efforts to make sense of the experience. Whereas some participants felt that guided self-help CBTv could be helpful during this sense-making process, others felt that this was something that needed to be done independently.

Jonathan: ‘It would need to be people who are ready for it. I don't know what time it is, I know when I would be ready.’

Sam: ‘I think you have to be at a certain level, a certain place to be able to engage with therapy in the first place, you know, wanting some changes and in a place where you are there.’

Although there is little consensus as to when guided self-help CBTv should be offered, all of the participants acknowledged the importance of timing and intervening at the ‘right time’. As discussed previously, hearing voices is a personal experience. It therefore seems reasonable to assume that the ‘right time’ to intervene is also a personal decision.

## The Presenting Problem:

### Voices

See main paper.

### Cognitive processes

See main paper.

## Networks:

The themes discussed thus far are all related to the development of guided self-help CBTv – this theme is not. However it was a theme that emerged across all of the focus groups. Participants spoke about the various relationships that they have, and unfortunately these were largely discussed as negative.

### Clinical relationships

Many of the participants discussed the mental health treatment they were currently receiving and the relationship they have with the practitioners. Participants were largely dissatisfied with these relationships. The most common issues reported were not being listened to, not being understood, and not having their needs met.

Sam: ‘It said [in the book] if you’re feeling as if life is not worth living you should seek professional help from your GP or mental health practitioner or A&E. From my experience I had tried, well I have wanted to commit suicide a few times, and I have never received any help. I have contacted A&E, I have contacted my mental health worker, and they have done absolutely jack sh*t about it.’

Lee: ‘They [services] give everyone antipsychotics every day and that's it.’

Catherine: ‘You go and see your psychiatrist and it’s what twenty minutes? And he will say ‘how are you feeling?’ And you’ll tell him, and he’ll say ‘Well you know Catherine, we all get this and we all get that.’ No we don't all suffer with hearing voices, and getting told to do stuff we don't want to do. So it’s like they’re not listening to what you’re going through and it, you know, throws you off init.’

The participants became very emotional when talking about these clinical relationships. The stories shared demonstrated a sense of disempowerment and helplessness. The participants spoke of asking for help and not receiving it. This sub-theme strongly suggests that the participants were unhappy with mental health care they were currently receiving.

### Nonclinical relationships

Similarly the participants discussed their relationships with friends and family under the same negative veil. The same points of disgruntlement experienced within the clinical relationships appear to be mirrored within these nonclinical relationships.

Catherine: ‘A few years back when I started getting voices people didn't understand it in my family, um especially my sisters, who I fell out with. You know they had no, they have never suffered with depression or anything like that, so to them it was like ‘oh you’re ill you know’. Your ill and its like ‘snap out of it’ and you can’t.’

Ed: ‘Like my family are in denial still, so I will tell them something that's been going on and they think it’s nothing, its fine. And that's one thing, that's every time I’m having a really good patch I fall back, because of the fact that my family are still not supportive about it and they think I’m doing something. So that's one thing that I think. Your family is your main stronghold so I think if you get them to also help it will be a lot easier process.’

Jillian: ‘I explained to my brother, um I told my brother that I was diagnosed with schizophrenia and he joked about it. And he said, you know, he thought it was a person with um Jekyll and Hyde, with two split personalities. And I said to him, I said you are so wrong. I said what you need to do is you need to go and get a book on schizophrenia, and then you will begin to understand me better as a person.’

The participants reported feeling like their family did not understand their experiences, and often tried to dismiss it (as reported by both Ed and Catherine). The word most frequently used by participants when discussing their relationships is ‘understand’. The participants are not asking for anyone to change their voices or make them disappear – they are looking for someone who can actively listen to their experiences and offer understanding. The responses within these sub-themes supports the findings within the ‘therapist’ theme, and demonstrates the importance of building a strong therapeutic relationship.

### Stigma

Within all of the focus groups, there were points where the participants shared their own experience of hearing voices. Voices were always discussed as being negative. However this distress was more than an emotional reaction to hearing voices, but also involved feelings of shame and fear of judgement. This sub-theme was most apparent when discussing the front cover the of the self-help book.

Nikki: ‘Privacy is important to me and this cover is quite loud. On a bus, it’s hardly coffee shop reading. It’s like ‘oh there you go that's what I’m reading today’ and it’s you could be anybody, like a professional looking into something. I just felt a little uncomfortable with the cover. I would want something more anonymous if I was going to take it out and read it. Just something a little more discrete because if I tell people I have mental health issues that's my business and sometimes I do and sometimes I don't, but if a book starts a conversation that's uncomfortable for me then I would find that a little, it sort of blows your anonymity a little bit.’

Lee: ‘I um I was thinking about that on the bus yesterday and I was thinking because I was on a packed bus with it [the book] and I thought if anyone asks me I will tell them I am a psychologist.’

Catherine: ‘I would say if you are on a bus and if you’re out in public and people are not aware, you can get some nasty comments or discrimination.’

The participant felt that reading the self-help book (which says ‘Overcoming Distressing Voices’ on the cover) in public would put them at risk of experiencing stigma. Lee was so aware of this that he had even considered what his excuse would be if someone questioned him about it – and this response would be to deny that he hears voices. Some of the participants reported even feeling uncomfortable reading the book around family and friends in their own home. As well as being distressing for the participants, feeling unable to read the book because of stigma could impeded therapy engagement. Consideration needs to be given to help patients engage in guided self-help CBTv in a way that does not make them feel ashamed.

### Group dynamics

This sub-theme differs from the other ‘network’ sub-themes discussed thus far. Relationships have so far been associated with negative beliefs and feelings. However this sub-theme describes the process of a positive network in vivo. Across all of the focus groups the participants treated each other with respect, gave each other space to speak, and fully attended to the person who was speaking. In one of the focus groups this mutual respect and support went a step further. Participants took the time to explicitly validate each other’s experiences, as well as sharing advice and ideas for coping strategies that they had found helpful.

Jillian: ‘[To another participant] I think it’s quite amazing. I think you’re quite special to have experienced voices for such a long period of time and still be here. I think that's amazing, so well done you, that's pretty amazing.’

Ed: ‘Yeah other coping strategies. One of mine, one thing they tell me to do is cut myself and I had a big problem with self-harm and that sort of stuff.’

Jeremy: ‘Yeah I went through that and all.’

Ed: ‘So I found one way of doing it is not to deny, like ‘no I’m not going to cut myself’. It’s to get a pen and actually do it with a pen just drawing lines on your skin instead. So it’s psychological in the fact that they [the voices] get something from it because you have done it but at the same time you haven’t caused harm to yourself.’

Jillian: ‘Yeah I think that's genius.’

Jeremy: ‘I don't know if anyone else has tried it, but I was told about it. If you’re out in public and you’re telling your voices to ‘bugger off’ just put your mobile phone to your ear.’

This sub-theme potentially provides evidence for the non-specific effects of group therapy, and support groups. Being in a non-judgemental space with other people who also hear voices seemed to provide some therapeutic effect. Previously the participants spoke about wanting to feel understood (see ‘clinical relationships’ and ‘nonclinical relationships’). Although the experience of hearing voices is personal, because of this share experience, the participants were able to empathise with each other much more quickly and on a much deeper level compared to someone who did not hear voices.

## Therapy Flaws:

This final theme describes the criticisms that some of the participants had of guided self-help CBTv protocol. This theme demonstrates that the participants in the focus groups were not ‘yes men’, and instead felt able to share both positive and critical opinions.

### Theory

Guided self-help CBTv is based upon the cognitive behavioural understanding of voices. CBT hypothesises that it is not the event itself that causes distress (in this case hearing voices), it is the beliefs that the person have about the event that cause the distress (e.g. believing that the voices are malevolent). After reading the self-help book most of the participants understood the concept of CBT, but not all were convinced by it.

Abe: “It says that ‘hearing voices in itself is not a problem’ but I can’t agree with that because hearing voices itself is a problem. Even if the voices aren’t being nasty or not saying anything to you, I have a terrible job trying to listen to two people at one time. Yeah so hearing the voice, even if it’s not being scary or disruptive, it's a matter of I don't want it to speak to me. I just want to concentrate.”

Abe did not agree that hearing voices itself was not a problem because of the voice’s effect on his ability to concentrate. The book also explores the application of the CBT model to beliefs about the self; it states that people who hear voices often have negative beliefs about themselves, but that these are rarely true. Sam and Jonathan did not agree with this part of the book. These objections to the CBT model could be explored and evaluated within therapy sessions. Alternatively these comments may evidence the importance of individualising interventions to the beliefs and needs of the patient, as a ‘one size fits all’ approach may not be appropriate.

Sam: “What happens if you don't believe you have a self? I mean it’s quite difficult. What happens if you don't think you actually have a personality? You don't have anything? You feel empty? I mean it’s quite difficult to kind of find something inside of yourself that's yours, when you’re just completely bombarded with voices, I mean what is you? When you start challenging, asking what you think about yourself, maybe you don't think anything about yourself anymore”

Jonathan: “There was something that I picked out, was um about negative core beliefs. I only read one chapter recently, and it was ‘the important thing to remember about negative core beliefs is in fact they’re rarely true’, and then it gave some examples of some negative core beliefs, and one of them was ‘I am weak, I am vulnerable’ but that could be true about someone.”

The self-help book also explores the notion that people are in a relationship with their voice, and that voice-related distress is often associated with a negative hearer-voice relating pattern. The self-help book suggests way that people can relate to their voices in a more assertive way. Some of the participants did not identify with the notion of being in a relationship with their voices.

Nikki: “For me I kind of see, I don't, the relationship with voices and giving the voices an entity, other than something you would have a relationship with, I didn’t really identify with but that's very personal nark for me about what was going on in the book. Because I don't give the voices an entity. When I hear auditory hallucinations or sounds or voices or things, I don't give them an entity or a relationship or a personality or anything because I see it as my own brain working too hard so I very much accept that it’s just my brain.”

Sam: “I have never felt that they have any sort of reference to anything whatsoever and they are just my mind playing tricks on me, and that um it happens most when I’m tired, when I’m overthinking about something um and medication, yeah, slows my brain and helps me calm down a bit.”

Abe: “I just have one more thing to say about the book. Not always, but in lots of the book they say we all have a two way conversation with the voice. I actually don't. I can shout, scream, speak at the voice and they will speak at me, but we don't have a conversation, a one-to-one conversation.”

Again these objections to the fundamental theory underpinning the intervention is something that could be explored within a therapy session, or suggest that the relating approach is not suitable for everyone who hears voices.

### Missing elements

While some of the participants disagreed with the theory of the book, some thought that there were key parts of their voice hearing experiences that the book did not cover.

Joe: “This just concentrates on voices, but usually there is a lot more symptoms that come along when. I know that when I have been ill there is a lot more going on.”

Sam: “Say if you believe you’re reading other people’s thoughts, that might, that's not hearing voices buts it's another sort of type of delusion that it can have, maybe, a relationship with maybe hearing voices.”

In contrast to the responses from participants within the ‘positive feedback’ subtheme, some of the participants did not like the symptom specific approach. Both Joe and Same felt for guided self-help CBTv to be effective for them it needed to give space to discuss other aspects of mental health such as unusual beliefs (delusions), paranoia, depression or anxiety. Other participants felt that the self-help book and therapy protocol did not acknowledge that there were other ways to understand the experience of hearing voices. Although this intervention is based on the CBT model, several of the participants reported that they would have appreciated the acknowledgement of alternative approaches.

Sam: “I’m not sure if this will be relevant, but I did think that they mentioned, sort of apart from the cognitive behavioural techniques, other ways in which you can deal with distressing voices. Like healthy eating, taking vitamins, like holistic therapies. Um you know all sorts of things that are quite beneficial to mental health but I don't know if that's going to be relevant, if they wanted to put a chapter in there about other ways to help mental health.”

Jillian: “I find it quite interesting that the only responses discussed are those that are emotional and behavioural what about logical and intellectual response as well as biological and physiological? So it’s not only just the mental side. Having um schizophrenia effects everything that you do. It effects your body, it effects how you eat, you know sleeping, um emotionally, intellectually, um and I thought that perhaps that should have been mentioned more.”

Ed: “I think all areas. Not just the medical. You have everything else as well. It would allow people to have a choice on what their experiencing and what they feel like they would be better to go and do.”

This was the most commonly reported criticism of guided self-help CBTv.

## Final Remarks:

At the end of each of the focus groups, participants were asked if they would want to receive this therapy if it was to be offered to them. All but one participant (95% of the sample) said that they would be interested in receiving this therapy. The one participant who did not want guided self-help CBTv was Sam. Sam said they felt they needed a more emotion-focussed therapy, rather than one that focussed on cognitions.

Sam: “The thing is, the fact that it's a cognitive thing wouldn't be helpful to me because I struggle with cognition in general. So it would have to be more of a feeling, it would have to be more emotional therapy rather than cognitive therapy for me yeah.”

# Study 2 (clinicians):

## Positive Attitude towards Therapy:

Many of the mental health clinicians that took part in the survey were very complimentary about guided self-help CBTv.

‘I can think of a number of clients who I think could engage and benefit from this.’

‘Brilliant! Self-help empowers clients, helps rebuild their self-esteem and take some control over something so distressing.’

‘I think this would be a good idea, especially in my area of rehabilitation and recovery. Most of our patients suffer from psychosis and a high proportion may be helped by this.’

This positive feedback generally fell under the following three sub-themes.

### GSH in the context of IAPT

Majority of the staff who were in favour of guided self-help CBTv acknowledged that access to psychological therapies for this client group is currently poor within their service, and that this intervention could be a way to increase access.

‘The provision of CBT within this Trust is awful.’

‘Access to CBTp is a real problem for patients.’

‘It could help increase access to therapy which is at the moment very poor.’

‘Seems like a good way to make a treatment available that people might not otherwise be able to access.’

The clinicians identified that having an intervention, like guided self-help CBTv that was brief could be one way to use current resources differently. The positive response from clinicians here suggests that many are open to the idea of briefer psychological therapies. Even more encouraging are the responses from several clinicians stating that they are hopeful guided self-help CBTv could be actually increase access in practice within the services they work.

‘I feel in my work and with the client group I currently work with it would be feasible.’

‘I think as a trust it would be fairly feasible to bring in these guides.’

‘Clinicians will make the time in order to implement this approach, even given the demands services are currently facing.’

### Staff willingness to be involved

Many clinicians supported their positive attitudes towards guided self-help CBTv with action. They reported their willingness to be involved at three different levels: (1) research, (2) attend training, and (3) intervention delivery.

Research:

‘My desire to be involved in this project is very high, the aim of the project is sound and patient focused.’

‘I would be interest [sic] to help in developing this.’

Training:

‘It will be useful for myself as a support worker so I am able to support those with these issues on a more professional level. [I] would be happy to go on training days to learn more about this.’

‘I would be happy to make the time to be trained to deliver it after research has tested its effectiveness.’

Intervention delivery:

‘I am already a Family Interventions in psychosis practitioner and I believe that being able to offer CBT as a guided self-help tool would be a valuable addition to the armoury we have to help our Service Users in their recovery.’

The commitment from some clinicians to act upon their favourable opinions is promising for the future of this intervention. Positive attitudes are encouraging, but for guided self-help CBTv to be successfully implemented this will require collective action from mental health clinicians.

## Negative Attitude towards Therapy:

While many clinicians were in support of guided self-help CBTv, there was also a significant proportion of the respondents whom did not agree or support this intervention idea. The negative attitudes expressed generally reflected either a dislike for the concept or disbelief that it would be effective.

‘I am very sorry not to be more encouraging but I see a number of problems with offering CBT for distressing voices using guided self-help in my work setting.’

‘No [I would not be willing to be involved] as this would worsen symptoms and increase risk.’

‘Not keen on this [guided self-help CBT for distressing voices].’

The most prominent criticisms of the intervention are described within the following two sub-themes.

### Not a stand-alone treatment

Related to the issue of effectiveness, some clinicians felt that guided self-help CBTv would not work as an intervention in its own right, and would need to part of a larger care package.

‘I do not feel that it would be appropriate as a blanket intervention in all other teams.’

‘It could be a co-treatment.’

Within primary care guided self-help CBT is a stand-alone therapy delivered within IAPT services. It seems that some staff do not seem to think this could be the case when applying this intervention to those who hear distressing voices. Although the notion of guided self-help CBT being delivered to those who hear distressing voices as part of a larger care package is not necessarily a bad one, it could mean that the issue of access would not be addressed through its implementation. Although resource would be saved by delivering this briefer therapy, this would be counteracted in the additional resources needed to supplement the interventions shortfall.

### GSH not an equal treatment option

Related to the above issue, is the concern expressed by some clinicians that this intervention would be used a way of cheating patients out of the more resource-intensive interventions they should be entitled to.

‘I would be concerned that guided self-help is used in place of face to face therapy.’

‘I worry that it would be an opportunity to reduce staff time with the service user and save money.’

This subtheme implies that clinicians believe by reducing the amount of contact time involved in a psychological therapy this consequently makes it inferior. Furthermore the clinicians seem to be concerned about the motivations for developing this intervention. Instead of attempting to create an acceptable, effective, and feasible to implement intervention, some of the clinicians think guided self-help CBTv is a way to save money.

This theme highlights some of the reasons that clinicians would not support the implementation of guided self-help CBTv. Prior to any attempts at implementation it would be important to reassure clinicians as to the motivations for and the effectiveness of guided self-help- CBTv.

## Support for therapy with a caveat:

For some of the clinicians, their opinions on guided self-help CBTv were more nuanced, and conditional on the caveats described in the sub-themes below.

### Importance of clinical training

Some of the clinicians believed that pure self-help would not be helpful for this client group and that they would need support from a mental health practitioner.

‘My experience of this group are that they will require a considerable degree of support to undertake this work.’

Interestingly, a number of the clinicians stated that this support needed to come from a clinicians who was highly skilled, and trained in the delivery of psychological therapy. Some clinicians were concerned that if a clinician who was inexperienced delivered guided self-help CBTv there could be safety and risk issues.

‘The provision of Self-directed CBT in voices needs the support and backup of trained staff to ensure patient safety.’

‘I worry that an inexperienced practitioner would be more likely to blame the patient if the intervention was not going as planned, further stigmatising a massively stigmatised community.’

‘I believe that practitioners working with psychosis should be experienced mental health professionals with sufficient experience, training and skills in this area of work. I believe it to be a very specialised area requiring an advanced practitioner.’

These responses suggest that the clinicians perceive people who hear voices to be a more fragile and risky population to work with, compared to other mental health problems where guided self-help CBT is delivered by clinicians without therapist accreditation (e.g. in the treatment of depression and anxiety within IAPT services). Whether the concerns of clinicians translate into real world risks will require further research.

### Need for evidence

Clinicians also acknowledged that the development of guided self-help CBTv was at its early stages, and that there is currently little evidence to suggest it would be effective in clinical practice.

‘At the moment I don't think there's enough evidence that CBT self-help for distressing voices is helpful for people, but that's why I think it is good that you are doing a research study to find this out.’

‘I would be a bit wary about offering CBT self-help for distressing voices as part of routine clinical practice, as the evidence isn't really there, but if it's part of a research study I think that's fine and that's the point of research.’

Even though these staff members were unable to give support for this therapy, they did seem to support the idea of trialling this therapy within a research study. This theme shows that many clinicians want to work within evidence-based practice, and that they use this evidence to make decisions within their clinical work. The fact these clinicians did not dismiss the concept of this intervention implies that if guided self-help CBTv is found to be effective then they would be willing to support its implementation.

## The Presenting Problem:

### Symptoms

See main paper.

### Cognitive abilities

See main paper.

## Practical Barriers:

### Lack of resources

See main paper.

### Conflict with service priorities

See main paper.
