# Supplementary material for: Barriers to disseminating brief CBT for voices from a lived experience and clinician perspective
Source: PLoS One. 2017 Jun 2;12(6):e0178715. doi: 10.1371/journal.pone.0178715 (PMC5456317; doi:10.1371/journal.pone.0178715)
Supplement: S4 File — (DOCX) [file pone.0178715.s004.docx]

# Study 1 Data:

## Focus Group 1:

Researcher: So, first things first we have already done introduction and have gone over ground rules. So I just wanted to know generally what did you think of the book. So who would like to start us off?

1: I will go. Well I found it quite therapeutic actually. I also looked at psychosis and schizophrenia, understanding psychosis and schizophrenia. This book is more user friendly. Um by XX. It is really sort of um, it characterised my schizophrenia really down to a tee. And comments in there by ruth and martin and their own personal experiences so I can relate to it. I haven’t actually read it all. I read it a couple of weeks ago. Um, no its good. Its quite therapeutic. So..

Researcher: Ok, thank you. Is there anyone else that has any comments?

2: I think what you said about having the examples, the case studies, I found really useful. Um, it was a good way of explaining theory of it. Um, and as you say some of it helped you to relate to it. So um yeah, I thought the case studies were a good idea and were useful.

1: I think it is really good.

2: It puts an example to it

3: The summary at the ends of the chapters is useful as well to just remember so that by the time you get to the end of the chapter you might have forgotten what was in it at the beginning. So it clarifies what you have read and reminds you.

Researcher: is that the bullet points?

3: the bullet points, yeah. It summaries the chapter, yeah.

4: …I found while reading it. It’s just very easy to read. And it’s just … and its accessible. Some of the things like tables and diagrams I though woo I wont be able to grasp it well and it was easy as well suppose. Its easy to follow.

Researcher: Ok. Is there any other general feedback?

5: I can relate to it well with my condition and it was written different. I can relate to it.

2: its quite basic so the language was simple and um I cant imagine um, so I cant imagine, how do I say this. I cant imagine professional reading it um but as I have just read on here um it would be, its possibly going to be used for supporting therapy. Um and I think it would work as that because its basic so that anyone could use it and um you know, you don't need to have a degree in psychology to understand the terms and stuff. Um, so so its yeah its quite accessible.

Researcher: these are all really nice things that you’re saying but I wondered is there anything that you didn't like? You can be honest.

4: I think what you said. Sorry whats your name?

2: Jim.

4: What 2 said, about the examples, a lot of the cases are quite, the book is quite basic really. And it's a bit sort of. I struggle to in bring my case into the literature. So I would really have to think and think and think so its not as straight forward for me really. Its helped me to identify only to a certain degree really. So… yeah.

6: yeah that's what I was going to say. It takes a very basic level of um sort of voice hearing and it can be a lot more complex than that. I think the book. I think its quite hard for any book to get one persons sort of view on voice hearing, their experiences.

Researcher: that's a really good point. So would you say that that especially came across when it was the case studies. Is that where that issue was most important?

6: Um, im not sure actually. I would need to…

1: I think it's the case studies for me. Its just how i … its just clarifies and summarises everything. The book is good. Its user-friendly. Anyone could read it. Anyone from society could read it. You don't have to be a psychiatrist or anything you can read it. Its very user friendly.

3: I thinking about what 6 said about the not being able to relate your own experiences to the case studies that were given. Um I equally didn't relate to the case studies that were given but I found that they, they made sense with the text that went before it. But I didn't um I felt a bit like I didn't, I didn't fit. My experience didn't fit and that made me feel a bit like, um there was something about different, almost kind of wrong about my experience. It was very different from the experiences given. Um I couldn't, yeah. I could relate to them as separate people. But I couldn't fit my own experience into that category, into that category. Um MARTIN’s experience, it felt almost too basic. He one heard one voice. Yeah that was MARTIN. I felt a bit, oh that's a bit, one voice once. Um most people have probabaly heard one voice once. Well, yeah.

Researcher: so I understand you. So the case study was good in that they explained the theory but they weren’t necessarily a good representation of what it is like to have this experience. Would you say?

3: my experience. It didn't fit my experience.

6: I just think you can be more complex. Um, and um I guess it depends on your illness. This just concentrates on your illness. This just concentrates on voices. but usually there is a lot more symptoms that come along when. I know that when I have been ill there is a lot more going on and I think it really depends on how, where you are in your illness. I know that if I had read this book a few years ago I wouldn't be able to use the stuff. It wouldn't really compute with you or anything like that. I wouldn't be able to sort of um use it as a self help book. but obviously further down the line its better, easier to use.

Researcher: well, that something that I wanted to pick up on actually, when do you think using this book either as a self help or as part of a therapy. When would that be best do you think? When you first start hearing voices, later on?

3: I think. At a point where the voice hearing wasn't too, too distressing or too… if im focussing on something that is specifically about hearing voices and how to help that situation my voices will not like that. and they will try and distract me from that or find a way around it um so that it doesn't make sense um they don't like being talked about. So if I was particularly unwell and my voices were already bad and I wasn't coping I think I would find it even more difficult to focus. You need to have a certain level of wellness in order to engage with the book.

6: Yeah, exactly its that insight… I think that you need to be told before you need to learn something a long the way about voices but um. I think, I mean when I first started hearing voices I honestly thought they were um they were people communicating with me and stuff so um and it took a long while to sort of learn that. the books great in what it sets out to um I have completely lost my train there, sorry.

Researcher: no that's ok. That's fine.

2: I don't know if I am thinking of similar thing to you. But um I think the book seemed to be a bit different to my experience of the voices clinic where its very an attitude of nobody knows what’s causing voices so whatever your belief is that's your belief. There just there to help you deal with them. Whereas I felt that the book wasn't like that so much. The books more saying well its caused by this that and the other. Particularly that first chapter, it almost felt like saying, it felt like it was saying that if I believe my voices were demons well that wouldn't be wrong, it could be because well if you know you look back to how it relates to relationships I have had in my past and all that sort of stuff. Then that's where my voices come from. It doesn't come from what I necessarily believe so I don't know if I just got that wrong. Or if that's what I picked up.

Researcher: how was that? kind of reading that?

2: it was ok at the moment, but I know at some points that would be well oh well well they don't believe so Im going to shut it. And um so if im in the bit where I totally and utterly believe what I believe in nothing I read or told will change that. So it would be yeah.

4: I think this book does … I think, I don't necessarily agree with the reasons that the voices that the book uses. I think it does it in a gentle manner and I can still relate to other sort of like, like angry sort of voices and um not incidentally I have been thinking a lot about my voices and at one stage you realise they are not telling me anything they are just sort of like getting at something. And I couldn't think what it was and it says here core beliefs … so reading this was very good because it seemed to consider your problem thinking about the voices it just sort of verbalised it.

1: its better than the wellbeing thing. Well mindfulness as well. The cbt side of it is just you can relate to it. Its so easy to understand. Yeah

6: yeah, I think it is good in that it does set it out like um the abs model and stuff like that I think that is really useful but at the same time it is trying to change what you believe and stuff like that . and that can depend on what, how you are, that could be quite hard to do. Even though it might be, technically the right answer. To do that it could be difficult. I don't know.

Researcher: Picking up on what you said there, so you think it would be different if it was part of a guided therapy. So if it was a case of hears the book, try and help yourself but instead was a tool that was part of the therapy would that change anything that you have said or would it not?

2: yeah I think it would because then you could talk about the fact that um what I would be able to say that what I believe is wrong and then you could talk that through um so um and then I might be more open to looking at things in a different way not being told that things are different but looking at things and being open to the fact that things might be different from what I actually believe at the moment. So yeah I think in a therapy situation it would probably work quite well.

3: I think it would be good in therapy because it gives you a starting point to talk from and often that's the hardest part I find, because knowing where to start or how to explain something and if you can explain it in terms of a comparison or pick out bits that relate to and bits that don't. you have a grounding there for discussion.

Researcher: it's a really good point. Would the fact that its part of a guided therapy make it difference as to when you would want this? So a few of you mentioned that self-help being quite difficult if you were distressed, so if it was part of a therapy would that make it easier to access it while you were distressed would you say?

3: I thinks it all psychological. I don't know.

*Apologising for interrupting each other*

6: I would just going to say if it was early or not with the therapist then it would be easier to have it explained to you and I could have wished that when I was going through stuff I had had a lot of voice hearing and no one would talk to me about it so I wish someone did so. Yeah it would have been easier to understand.

5: It may prevent you to going to hospital because when you go to hospital you have a lot of sitting there and thinking. But thinking sometimes makes it worse because it um even if we had this therapy in hospital, maybe that would be a good idea.

Researcher: so you could see it working in a hospital?

5: yes, yes. I would you know once or twice a week or something.

Researcher: ok. There are a few specific things that I wanted to move onto. But we can come back to anymore thoughts that you have at the end. So the idea that we have got is making this book into a guided therapy um and in the packs that I have given you there is sheet that has session ideas. So I wondered if you could take a couple of minutes to have a look at this and this is kind of like a template for therapy idea that we have. But it is up for debate basically. So that's one of the things that I wanted to ask about today. So um I just wanted to kind of think about the kind of topics that would come up within the therapy?

6: what the voices say, possible sort of beliefs which might be hard if someone is quite, well I was quite delusional when I was ill and then when I got better I realised that I was quite like that so, yeah.

Researcher: so just to kind of expand it a bit more the idea behind this is that in the book it kind of picks up on three different themes. So it talks about beliefs about the self, so the core beliefs that you mentioned, um and that's kind of one topic. And another topic seems to be thinking about voices and those beliefs. And another theme in the book is relationships. So these are the three themes that we have picked out but I wondered if that matched what you took from the book at all.

6: I thought the self esteem part was good as well.

Researcher: ok so the self-esteem part was quite important, that's something that we can definitely make sure gets included. but how would you feel if you were going to a therapist and this is the kind of template that you were given for your therapy how would you feel about going into this kind of therapy?

3: if I was given the whole template at the beginning I might feel um a little bit reticent because, because I guess the word challenging where you know you’re going to talk about one thing on week and the next you will be challenged on it. And that might feel quite scary um because something you are going through… and someone is going to challenge you on it um and it automatically make you think that the person doing the therapy thought that what I was thinking was not correct and they were going to challenge me on it. So your going to get all of my beliefs out of me on one week and then challenge all those beliefs a week later I think yeah I think yeah. If the theme comes up week by week without the titles it might be easier.

Researcher: Ok, so more of a subtle less of a divide. More of a subtle talking about beliefs maybe trying to questions them.

3: How to manage your beliefs maybe? Rather than challenging you beliefs because the word challenging is a challenging word.

1: I agree.

6: at certain times its not sort of possible to prove or disprove your own beliefs as well so it going to be pretty difficult. Yeah.

Researcher: how do you feel about the kind of structure. So if we were going to stick with these three topics, thinking about the self, and maybe bringing self esteem into that, then voices, and relationships. What do you think about the kind of order that we have proposed. Does that make sense to you?

Multiple voices: Yeah.

Researcher: are there any comments that you want to put forward.

6: beliefs about voices before challenging beliefs. Oh you put challenging beliefs 2 after. Oh ok sorry.

Researcher: what do you think about the idea of self and voices then relationships? Does that make sense or would you rather it was different?

3: I like it that way round.

Researcher: is there any reason why?

3: mmmmmmmmmmm it make sense to. Because to look at yourself first because your *coughing* its understanding a bit about yourself maybe and is being, working on your self esteem and then the voices will be the next biggest thing you need to deal with. So you deal with yourself then you deal with your voices and then the next thing you need to deal with in life is relationships. So you can look to your relationship with yourself, your relationship with your voices and then your relationship with other people. That would be the order in which I would prioritise for myself um my voices are bigger than my relationships sometimes and I should be bigger than my voices so that would make sense.

Researcher: is there any other thoughts people had around that?

2: I agree that um beliefs about self should come first because that gives a sort of basis for everything else so um you cant um sort of um when we are talking about voices you are obviously talking about the way you are reacting to those voices and so yeah it so you have got to talk about yourself first. And the same with relationships obviously you are involved in any relationship that you have so yeah it would make sense that order.

Researcher: any other comments about that at all? Ok, I wonder if any of you had had a chance to have a look at the activities that are in the book at all? Did you have a go at any of them at all?

6: er yeah. Most of them as I read through the book.

Researcher: ok. How did you find these?

6: um yeah it was interesting. It sort of er puts like a scan on where you are and stuff.

Researcher: we had the idea that these activities might be a helpful thing to do within the therapy. Maybe you and the therapist trying some of the activities together. To do those is that something that you think you would need to support to do or something to do on your own?

5: would need support with that. yeah.

Researcher: we have the idea around the book of also making like a workbook to go along with the self help book I wondered what you thought about that?

2: I think it would be a really good idea because I always find that whenever I got therapy or anything like that um its fine while im doing it but then its hard once I have gone away weeks later or when im facing the same challenges I cant put into practice what I have learnt because I forget some of it. Whereas if you had the workbook and you had a record of what you had gone through you could flip back through that and that would be a reminder to be able to put those things into practice again so um so yeah I think it would be a really good idea.

Researcher: ok anybody else got any ideas about a workbook to go along with it. So in the workbook it would be some of the activities in the book. we would copy those and put those into the workbook and you could have a go at them more than once. The only problem with having them in the book is that when you have done it once then that's it. So you could have multiple copies. How does that sound as an idea?

4: yeah I was. I mean I thought it would be nicer to have photocopies in a larger format a workbook would make this, yeah.

Researcher: yeah that's exactly the idea. The book is quite small isn’t it and its quite difficult if you wanted to make notes and that.

6: then you could see your progress over time as well with those parts, any difficulties you have, similar any questions

3: it would give you a much more person copy of the book if you like. I mean I was saying that I couldn't relate my experience to the experiences of the people in the book so if I had a workbook that my therapist helped relate my experience to then yeah then its sort of a way of having my own personal sort of my experience as opposed to MARTINS or, I have forgotten her name, oh ruth. It would be my own personal experience

Researcher: so this book would kind of just guide you and explain how to relate it to your experiences?

3: Yeah, yeah. It would to actually write it down how to makes it much more person centred.

Researcher: that's really helpful. In terms of how we would present that information like the workbook. Would you prefer to have a week by week workbook as you were going through the therapy, so for each session or each topic. Or would you prefer a kind of whole workbook so we give you the whole thing and you would have all the information at once. What would be best?

3: I liked it with the sessions that I have with the four sessions in the voices clinic they give me a little book that I liked that was the whole book. Although I worked through it week by week I could see exactly if I wanted to I could see what I was going to be doing that week like so I would only do week one but I could have a look at week two before week two so I formulated some ideas in my head about what I wanted to talk about or ask.

Researcher: does that match other peoples opinions at all?

2: here I got as far as but afterwards having it all in one hand booklet or whatever would be useful because its all in one place and you can flick through to the bit that's relevant rather than having lots of different bits of paper that can easily get lost. Um yeah.

Researcher: I wonder whether giving it all at the beginning of therapy as one workbook could that be overwhelming at all. That was one worry that I had but I don't know if that is justified.

6: I think it would depending on when the therapy would happen. during the course of an illness or something I think that could be quite overwhelming.

Researcher: what do you think the consequences of that could be? If you were feeling overwhelmed in terms of your engagement with the therapy

6: well you wouldn't want to do it or you wouldn't do it you would avoid it.

Researcher: I mean that's something that we could make optional for people. We could give them a choice I suppose at the beginning. I suppose if they would prefer to have it all together or separated. Do you think that would be a good compromise?

Multiple voices: yes

Researcher: Ok, I wondered what your thoughts were, whether you could see any problems related to kind of things that were going on for you in terms of being able to engage in a therapy that uses this book.

1: for me I found I was reading myself a lot I could relate to it. I cant reiterate enough but just theres more empathy … I think the workbook could be too complex to read. It depends, it depends on how easy it is to read. Because some people have dyslexia. I mean when I was ill I was sectioned and now the voices are quite bad I hear the voices every day. So it seems good I thought, I was telling my psychiatrist I was hearing voices and I thought it was just me, I thought no one else had it. And schizophrenia or whatever diagnosis people have. I don't know if people have different diagnosis probably in this room. I don't know. Have you read this XX? Understanding psychosis and schizophrenia? Its really hard to read.

Researcher: picking up on something you just said, the book you found ok to read in terms of the language. So if we were going to make a workbook would we need to make sure we keep it at that same level?

1: yeah at the same sort of level.

Researcher: Ok.

3: Another thing I thing I find, especially if im unwell is concentration. I find it really hard to read um at the best of times lets alone when me concentration is down because im more unwell um. I re-read the same thing so that could be a barrier to accessing the therapy.

5: I agree with that. that's why I couldn't finish this book because I read it over and over and haven’t got the concentration to read it all very quickly.

4: I haven’t read a whole book in years and this is one book I just read pieces, a chapter or two … I read as much as I could. I couldn't really…

5: that's like me. I had very bad concentration. Terrible, took a long time.

Researcher: is there anything we could do in the therapy to help that? so just to make it clear we wont be asking people to read the whole book before they start but it might be a case of you having therapy this week and this chapter is kind of relevant to what we are going to talk about so you might want to read it before you come. Do you think that kind of approach? Is that too much still?

3: I like the bit that you have at the bottom about um summarising it if you haven’t read it um so it doesn't mean you cant engage with the session um I find, I find if I am set homework to do I feel under pressure to do it and then I put it off. Because I think oh I cant do that its too much or I cant get started with it um so yeah it would have to be presented in a way not as you must read this before this date unless we cant carry on with what we are doing I think to say it would be helpful if you read this but don't worry if you cant kind of thing we will go through it at the beginning of the session anyway. Would be a better approach.

Researcher: how do other people feel about that? I mean like if we use that more casual approach, you can do it if you want but it might be helpful but no worries if you don't. how would that suit other people?

1: I think informal is good, casual informal.

2: I agree with that because um I have been in groups or courses where its almost like being back at school and you feel like your going to get penalised for not doing the homework. And its like well if your not going to help yourself then we aren’t going to help you sort of attitude. And that just puts right off straight away. Whereas if you know that theres no pressure and you can do it in your own time and um if you know theres no pressure there isn’t that barrier to do it um but yeah. Telling people they need to read it doesn't really achieve anything I don't think.

Researcher: so for the summaries. We understand that some people might find it hard to read a whole chapter so that why we wanted to do summaries so what kind of length of a summary would be acceptable. Is a page too much?

1: Like 5 sentences, four to five sentences really.

Researcher: so would you bullet points?

1: Yeah

2: I would say like half a page of a4 with bullet points.

Researcher: I don't know if any of your noticed um 3 picked up on this there are summaries at the end of each chapter with bullet points. Is that the sort of thing you think might be helpful?

Multiple voices: yeah

Researcher: one thing I would like to ask about is if we did use this as part of the therapy how often would you want to meet with a therapist and how much time would you want between sessions?

2: I think generally a week is a good amount of time because

1: yeah a week.

2: um because then you haven’t forgotten everything from the last time and you have had enough time to digest what has been said and think about the next bit that you are going to read and have a think about what you had read and yeah any longer than that it feels a bit it gets a bit disjointed um so yeah I would say a week would be ideal.

Researcher: what do other people think?

5: yeah once a week would be quite good.

Researcher: once a week would suit you?

5: yeah.

*1 laughing*

Researcher: are you alright bob?

1: yeah too much sugar.

*everyone laughing*

RESEARCHER: I wondered 20 did you have any thoughts. How ooften would you want to meet with a therapist?

20: Once a week would be ok with me.

RESEARCHER: Would that feel like too much or too little?

20: average. Just right.

Researcher: and I wonder in between sessions. So if we did go with once a week, would you want any contact with your therapist between the sessions at all?

1: for the mindfulness in the voices clinic we get, don't we Joe, we get reminders on our phone. I think that works quite well.

6: yeah they remind us to do mindfulness. Yeah.

1: the meditation.

20: they give you a text to practice mindfulness

Researcher: so you get a text to practice?

20: yeah.

Researcher: How often do you get those between sessions?

1: twice a week.

6: yeah.

Researcher: how does that feel twice a week?

1: its not pressurising at all no, no its quite um refreshing really. It lets you know that they are thinking of you.

Researcher: how do you find those?

6: yeah its good, its good. Its just a nice little text saying this is a reminder from the voices clinic to do your daily mindfulness. That's all it says. Yeah um its good because otherwise you forget and

Researcher: ok so text seem to work for people. In another study of a new therapy that they have done in another trust as part of the therapy the therapist always makes a phone call in between sessions. Do you think that is something that you would want or do you think that's not necessary?

20: not necessary

Researcher: ok, do you think texts are fine?

20: yeah.

Researcher: I wonder if anyone else had any thoughts around that

6: It depends what the therapist spoke about, would it be the same or.

Researcher: so the way that they have done it in the past although that again is up for debate is they kind of called just to check in see how you are doing., go over what you did in the last sessions and the goals that you might have set and see how you are getting on with them. So I suppose it's a bit like the texts in a way reminding you of the kind of things you said you were going to do. But I wondered how that sat with people, a phone call as opposed to texts?

3: I find that a little bit intrusive. Because who knows where I will be when that call comes um and I might feel a bit like im being chased up and I have to a think of a response, whereas if it's a text message its up to me what I do with that.

Researcher: thats a really good point. I hadn’t thought of that actually.

2:also with a text message it will let you choose when you respond to it so its not necessarily sending a reply but um if its like with the voices clinic reminding you to do the um meditation then you know you get to choose when your going to do that. whereas if it um, what 3 said about um if it's a person it might be a bit intrusive and you know you suddenly have to be like well im doing this right now. yeah I don't think id like that.

6: yeah id be the same I wouldn't like it. You know you could be having a bad day and then you know your not ready for a phone call

Researcher: I hadn’t thought of those ideas. So if we use the text approach and if it came from your therapist so it was personalised to you talking about the goals that you had set would that work for people.

Multiple voices yeah.

Researcher: ok that's a really good idea. That's something I hadn’t thought about. Ok so we have talked about this a little bit about this but I just wanted to talk a bit more about this. Who do you think this intervention, who would be the target market? Obviously people who hear voices. you all have some experience or knowledge about services so what do you think if we took this into early intervention would that be like would that work or is that too soon?

4: it would need to be people who are ready for it. I don't know what time it is, I know when I would be ready, its this time.

Researcher: so you think too early on..?

4: I think too early on its not helpful really

6: when I think back to when I was really ill this would I wouldn't have you know even though I would have tried to read it you know I wouldn't have like you wouldn't have got through to me in any way. So maybe after someone has been ill or something or recovered from psychosis.

Researcher: so if im understanding this right. So after that first kind of experience, so maybe when you get to know your voices a bit more, is that what I am hearing?

4: I think the way it worked for me I started having some sort of intuitions the voices saying this and that and it expanded and it confirmed my suspicions. I have been in the services for quite a long while and not being able to talk really about voices and the book confirms my intuitions and that was the greatest thing really.

6: it does aid your insight it gives you insight but so I think you know the voices um whats the word um are attacking or something you know and they can be like that and um so it definitely gives you insight but when that might be helpful im not sure.

Researcher: when exactly is quite a difficult question but im getting the impression that early on is not a good idea?

2: its all too confusing early on and theres all too much going on and you don't really, your not stable enough um to um properly think about or talk about your voices from my experience I didn't want to talk about it because I was really embarrassed about and to be forced into that position it would be quite damaging I think um so I think early on you are definitely, it could either be a waste of time or it could be damaging I would say.

Researcher: that's very interesting.

4: what about talking to them in the services and giving them a form to see if they are interested something like that because maybe some of them in early stages would be ready I don't know maybe someone, might be ready.

6: do like an assessment on each individual person to see do they relate to each of these certain things, these bullet points of these things. That would be helpful

Researcher: so tell people a little bit about the therapy and see if it would appeal to them

6: yeah and maybe you know the book it does give you a lot of insight into it so maybe give them the, definitely helping people early on with it would be helpful but I think the whole process of trying to challenge everything would be too difficult. Well I know it was when I was that ill and stuff.

Researcher: do you think its about being at a certain stage or at a certain place to be able to engage?

6: yeah. I think definitely with the challenging the beliefs and everything like that you, um because um well if you really believe the voices are someone from here or there or somewhere well you believe they are coming from, whatever your belief is to sort of challenge to challenge anything that you would need to be open to it

4: you would need to be challenging yourself and starting to do that

6: yeah just having that recognition I don't know depending on what you believed before that you could be like I don't know

3: I wonder if it would be useful for people um in early intervention just to give them the resource list um at the back so that if they decided they wanted to find out more um or access some kind of support then they've got that list they can then look at themselves um and if they are not ready then they don't have to. But you give them something um so your not ignoring the fact that they hear voices you have acknowledged it you have told them they can look up the hearing voices network or this or that or the other and um yeah and they can then through that maybe get to a place where they are ready to this kind of a group um maybe just a bit of self discovery first

Researcher: you mentioned just then about this being a group. We actually had the idea that this would be an individual therapy so it would you working one on one with a therapist and I wondered what you thought of that actually because that's something we haven’t discussed yet so it wouldn't be part of a group therapy it would actually be you one on one with a therapy kind of working through the book. How would that suit people?

3: That would make it easier because if you hadn’t managed to read the book for whatever reasons you wouldn't feel compared to other people in the group you would just be you like you said um whether or not you or how much you recap or don't recap on the chapter would depend on you know how much you have read or how much you have understood so you could go at your own pace I think definitely as an individual therapy it would work a lot better

6: for you to get the most out of it you know in groups its good because you get other people experiences and you hear about it but also but with one to one I thinks its better because you get you get that really personalised help and um that's like invaluable I think yeah.

2: because you are like talking about your own beliefs in yourself and your own voices because it is a very personal therapy personal subjects I think it would work better on a one to one basis um because obviously everybody in the group is going to have a very different experiences and beliefs and um do I don't know if you would sort of maybe waste time sort of listening to sounds awful wasting time but you would spend a lot of time listening to the individual differences and you wouldn't have the time to focus on how your going to challenge or what your own beliefs are in fact so yeah it would work better on your own

5: and you might not want to open up about your own um experiences really so it might better I think

Researcher: I know you [jack] have done some group work how would you feel about doing this therapy one to one?

20: I would prefer to do it one to one because then you can talk more people wont pressure you to talk about things that you don't want to talk about

Researcher: that's good to know. Its good to hear that our idea is something that would work with you. We started talking about kind of when it might be helpful. Were there any other things that might be going on at the time like when you hear voices that would stop you from getting on with a therapy like this?

6: I couldn't even hold a proper conversation when I was really ill. Yeah I had so many voice hearing going on I was too distracted so to engage in this would have been virtually impossible.

2: yeah I think um if I was really ill at the time um it would be too much for me. I wouldn't be able to engage with it I wouldn't be able to concentrate. I wouldn't be able to even think about what my own beliefs are and take too long to, because I would lose track of myself and lose track of what my own voices think and um it all gets a bit muddled up it would be too much to do it when I was really ill.

Researcher: I wondered if we could think about, the book talks about different types of voices. so there are voices that can tell you to do things, there are voices that can be abusive and things like that. are there certain types of voices that you think this would work best with or do you think any person hearing any kind of voice could get on with this therapy? Does that make sense what I am asking? There might be people who hear voices that tell them to do things and there might be people who hear voices that are abusive. Do you think both of those kind of groups would work with this therapy?

4: yeah. Any distressing voice really I think.

3: could be difficult um if. To get the boundary right if your hearing voices that are telling you to do things that are maybe dangerous or um could hurt you or hurt someone else and you ask to talk about that, the therapist then might have a real problem about whether they need to report that or not and where to go with that um or you might not be able to tell them in the first place because eyour voices wont let you communicate that to someone else um and if you yeah and if your voices are not letting you talk about what they are saying then that's not going to work and if your voices are saying to do things that are really dangerous or um yeah then I think it would be very hard to bring those things up in this setting especially because it might get you to look at what the voices have said and then you are waiting a week to before you look at how you might be able to challenge that so I don't think im getting my point across very well. Just yeah I don't know how it would work if you were to try and communicate stuff that was really dangerous.

Researcher: with things like that we would have to follow trust risk procedures. So when we first met I talked to you all on the consent form that puts yourself or others at the risk then we would need to follow that up. So that's one thing we wouldn't be able to change. So if you did say anything about voices that presented a new risk then that is something that the therapist would have to

3: that could be a real barrier to starting the therapy. If you knew that were the case

Researcher: that's one thing that we cant change sadly. Its all about safeguarding. For those of you that have done other studies or have done the voices clinic. It is a standard thing that we have to put on all the forms yeah. So you said about voices telling you to do things and then not being to talk about the voices because they don't want you to. If that was the case do you think you would still be able to get something from the therapy or would it be a waste of time. Its quite a harsh way to put it but hopefully you know what I mean

3: I think you can get something from it um you might even be able to challenge the belief that you cant say what they are telling you you cant say, that might actually be a really positive thing um I think it what you get out of it will vary hugely from person to person really

Researcher: I wonder if you had any thoughts about diagnosis at all. About whether anyone who hears distressing voices could receive this therapy. Obviously people with lots of different diagnoses hear voices so do you think that's the approach we should take here so anybody with anybody diagnosis can receive this therapy?

3: definitely

1: yeah.

4: distressing voices that they are unhappy with. They sort of like start challenging themselves perhaps and then they can find some help and guidance for that

Researcher: so diagnosis doesn't seem to be very important for people. That's really helpful to know. A few kind of other things that I wanted to talk about if you were kind of coming into the therapy what would be your main goal. What would be one thing that you would want to achieve?

1: no more voices!

*multiple laughs*

Researcher: sadly we don't have a magic wand. I wish I could Bob

1: aw bless you.

5: I think it would be good if you could switch it off, learn to switch it off.

1: its hard though isn’t it

5: it is hard yeah yeah

Researcher: is there anything that you picked up on from reading the book and thought oh I would like to change that actually. Is there anything in particular that you thought would be a good change to work towards.

2: I think the challenging beliefs in the power that the voices have. You know when they are really loud and when they are really insistent I think that what they are saying is true and um and that they can make me do things that if I could challenge that in that moment then that would be you know that would completing change everything really.

Researcher: how does that sound for other people? Like changing how much control you have and how much power the voices have

4: I think the voices themselves are not as bad as the thing they can do with you in terms of how you respond. I mean it could be self neglect or some other things. I always think that if I only heard the voices then it would fine but it's the other problems like how I respond to them

Researcher: just to pick up on that if there is something in the therapy about how to respond to voices more helpfully?

4: to be more assertive yes, and challenging the belief that they do things

Researcher; that is really helpful yeah. Was there any other idea that people had. Any other things that you would want this therapy to help you change. We talked about [interrupted]

1: understanding my, sorry to interrupt, just better understanding really it just gives you that personal, it just gives yourself that personal touch on things just so because we are all individual everyones different unique and we hear all different voices and stress and anxiety yeah you talk about diagnosis, diagnosis could be anything from psychosis drug induced psychosis schizophrenia. Its difficult but I don't want to categorise myself I want to get back to work so I have only been diagnosed with it for two years. You can sort of, what 4 was saying, sort of switch them off, not hearing the distressing voices but its difficult its difficult

Researcher: yeah that's it. I mean there are these things that are difficult, hopefully this is something that we might be able to help you with. So what kind of are those difficult things? So you mentioned responding to the voices in a different way, and challenging the power. I wondered if there was anything else?

6: just getting an insight about voices any insight is helpful um

1: a dvd would be good. Because I am a visual person so watching something might be good. A dvd on that. but I suppose you are talking about more money then to do something like that.

Researcher: it might not be a case of us having to make a dvd but it could be a case of us finding sources that are already out there. I think someone picked up on the fact that there are a list of resources at the back so if we could find more resources and ones that are visual then that is something we could do. It wouldn't necessarily involve us making them. I mean I am no Steven Spielberg so I wouldn't want to try and make a film. I think it would be quite embarrassing. Someone mentioned self-esteem earlier and I wondered whether you thought that would be an important thing to include in the therapy because it is mentioned in the book.

1: we all have different confidence levels don't we. We’re all difficult levels of confidence are we

3: self esteem is important because if your self esteem is really low then your less likely to be able to challenge your voices because um I think you give them more power you see them as bigger than yourself um and you wont kind of be able to have enough self esteem to even want to challenge them you just along with letting them beat you around

6: I think any illness self esteem just plummets anyway and like during recovery I just think just any sort of help will be useful I think

Researcher: did you have any thoughts on self esteem at all [to sue]?

5: yeah um I cant think of anything.

Researcher: that's fine. Ok

5: I was going to say if your working you have more self esteem like you want to go back to work you have more self esteem if your at work and when your not working you don't have as much self esteem really., you bring yourself down because other people have more um they have jobs and houses and cars and when your out of work you cant gain those things really so your self esteem goes really yeah. You feel worse about it.

Researcher: jus to pick up on something you said there. Do you think we could include something in the therapy trying to get people more involved in activities?

5: yeah that's a good idea.

Researcher: do youy think that would be relevant? Or is that a step too far?

4: it could just be one of the outcomes, one possible outcome for certain people.

5: what kind of activities?

Researcher: there are lots of different groups, I mean like the recovery college, getting back into work or volunteering. Ok, so that could be one possible outcome but again is this one of those things where it would be an individual question what do you think? So the outcome of the therapy and the focus of the therapy would that be something that you would want to decide from the outset with the therapist?

2: yeah definitely. I think if um there were desired outcomes already set out you would you know feel like they were forces on you and if they didn't match up with what you were looking for then you wouldn't want to do therapy whereas and you would sort of loose track of all those other things they were going to help you with because the outcomes say oh we are going to get you back into work or help you do this that and the other, and well that's not what Im looking for

6: I don't think you should be so rigid. I mean event these topics I think they need to be, theyre good because their quite universal but at the same time they cant I don't think being too rigid is going to help anyone um yeah

Researcher: well that's one thing I wanted to check out with you. We have these three topics here: self voices and relationships. If we kept them broad at that level is that three topics that you think would suit everybody and then you would have the more personalised details. How would that suit people

3: I think it would work

2: I think it would work.

Researcher: so if you met with someone and they said we are going to talk about these three things how would you feel going into the therapy?

6: depends what issues you got at that present moment. You might need different sort of help and um yeah. I think its pretty good. Yeah.

3: they are things that everybody has to deal with if you hear voices. you have to deal with your voices, yourself and your relationships. You cant escape those things.

Researcher: is there anything major that's missing would you say? Is there anything that you think we have missed?

6: I was thinking storylines and stuff like that but its hard to come across like because its going to be so individual to a person like delusions and different things you know. Its hard to

Researcher: what do you mean by storylines?

6: um because well when I was ill I had different storylines going on with different voices and that so I don't know if other people had similar I have never actually asked anyone so. The book doesn't, I guess it does cover it in terms of beliefs so it would cover that so yeah

Researcher: that's really interesting though because most people do hear more than one voice and they can be different so that's something we would have to work on for the therapy. So do you think its something that you could use to look at all your voices or would it be helpful to look at one and stuck with that throughout the therapy? What would you advise people?

3: I think it would be different for everybody depends on which is more a dominant voice or for me my voices are all equally present and dominant so I couldn't pick one over the others they all work together as a group um whereas another person might have a really dominant voice and some more that aren’t so um you might choose to work on the dominant one or a voice that's troubling and a voice that's not troubling so you might just work on the troublesome one.

Researcher: As somebody who does hear more than one voice do you think you would still be able to go through these topics

3:yes.

Researcher: And engage with them?

3: yes because my voices are one unit so although there is more than one of them they are all talk about the same thing or say similar things they yeah kind of work as a gang.

Researcher: how would that fit for other people and the experience of more than one voice or as you put it the multiple storylines., how would you find getting along with these topics

6: um

5: when it covers voices does it cover hallucinations as well because um some people hallucinations with voices

Researcher: voices are also known as what you would call auditory hallucinations

5: oh I see. They are one and the same

2: is it possible to open a window its really hot

*open window*

Researcher: ok so we have talked quite a lot about what the therapy is and who it would be for I wonder if we could talk about the therapist now. what would you want out of the therapist?

1: someone who is kind and compassionate someone who can use empathy you know because its when you talk to all these doctors and psychiatrists you feel like they cant relate to you because they are sat in the chair and their professionals and their reading from their textbooks you know you want to feel you know like their looking down on you, not looking down on you but they can sometimes not got experience of voices or self harming or not aware of the sexual abuse cycle you know the physical abuse you know theres lots. Have they been in a domestic relationship. …Getting sectioned. But just someone who is kind and compassionate someone whose can understand you

3: I have noticed a difference in working with people from the voices clinic to just the general therapists and I have found that the people with specific interests and training in working with voices um are able to extract information from me in really helpful ways and teer conversations to bring it back to the voices and not going off on a tangent and getting lost somewhere else. It felt a lot more useful if I know im there just to focus on a specific topic around voice hearing and not all the other things that might be going on for me um and somebody that can keep that boundary I guess and keep bringing you back to that place without you feeling like their ignoring what else your saying um so they need to be quite skilled in think in already you know working with voices

Researcher: is that something that other people would agree. Do you think we would need people involved in this study that have this specialism and have that experience of working with people that hear voices?

1: specialism that's a good word.

2: I think what I was going to say is someone who knows their stuff but doesn't have the arrogance they think they know it all so kind of what 3 was saying about um so having that specialism and yeah have that experience of working with people who hear voices um but also having the compassion to want to help not just because theyir interesting to work with but because you know they do have that passion to want to help people um and that's what I found with everybody I have met in the voices clinic, so yeah I don't know how you find those people but keep finding more

*laughter*

Researcher: well hopefully people from the voices clinic would actually work on this study.

1: what someone high up with loads of credentials.

Researcher: I don't know about that. not sure I could swing that

*laughter*

1: I have actually met XX he is a lovely guy. Very compassionate, he wrote this, hes a really nice guy

Researcher: he is part of the team on this study

1: fantastic guy so

Researcher: hes my boss so of course I agree with you

*laughter*

1: one two one two testing

*laughter*

Researcher: so we have gone over the personal qualities of what you want in a therapist. if you could kind of pick people from a team, so like a clinical psychologist who has experience of working with people who hear voices so we would hope that they are very lovely people and have that empathy does that sounds like a suitable person?

Multiple voices: yeah

4: or a trained counsellor

Researcher: ok so someone with experience and the expertise is that what I am hearing

4: there is a difference between knowing about voices and knowing how that can feel the experience of hearing voices so it is needs to be really practical really

2: and somebody that can actually feed back to you and not just sit there and listen whereas there is a place for that in some therapy or whatever in this sort of thing it needs to be somebody that can feed back to you and um like 3 was saying teasing information out that relevant so somebody that can do that

Researcher: that's really interesting so like you said the experience, so their expertise their being trained in delivering therapy but also the kind of knowledge around voices that seem to be really helpful. So the last few questions I had here, I just wanted to talk about any advice you had for us taking this forward as a research idea. What would you kind of say to us?

1: just genuinely just speak to people with voices and you will get a concept an understanding really um just listen, active listening just that's always good um

Researcher: I don't know if any of you been involved in research studies but you would normally meet with someone from the research team and you would do an assessment and then you would have the therapy and then there would be another assessment afterwards, that's generally how it is. Is there any advice that you would have for us in being able to actually take this forward as a research study, anything we need to be aware of?

1: I think your enthusiasm is great. Honestly XX your great. Both of you have got your professionalism and your enthusiasm down to a tee. So as long as they are all like you.

Researcher: thank you Bob. Is there anything for the running of the research study?

3: well we touched earlier on on the people who may or may not be suitable um to do this kind of um therapy so the initial assessment would be really important to gauge if someone was in a place that they could manage to read the material um from the book that they could um that they are ready to challenge those beliefs um and that they wanted to move forward in that way um if they weren’t able to do those things or weren’t ready or wanting to then trying to engage them in a research study would be pointless.

6: and also people issues and the issues I know I had I had a lot of help afterwards even after I got better started to get better and it doesn't really fit into beliefs about self or beliefs about voices their my own thoughts sort of thing so um I think without I just think there could be lots of other issues that could, but I guess that's more to do with other kinds of counselling that you might get as well. I guess you could fall into that um just talking about other issues whereas you want it to be more sort of about overcoming distressing voices.

Researcher: yeah so this therapy is taking the symptom specific approach. So the idea that we can then create a brief therapy so this would at 8 sessions would be considered a brief therapy and we are going to target something specific and make a specific change. You make a very good point that people have lots of other things going on for them. But we would try and make it clear upfront that this is kind of therapy and this is what we set out to do

3: I would like to volunteer to have the therapy. It sounds great

Researcher: that's actually a question I was going to ask you all

*laughter*

*phone goes off*

Researcher: that's picked up on something that I wanted to ask you all about this symptom specific approach focussing on just the voices. how does that idea sit with people?

3: I really like it

1: I would be fine to go with that yeah

3: um I found the four sessions that I had with the beginning of the voices clinic being specifically about the voices really helpful because out of I think everything that I have ever struggled with that's been the one thing that's always been ignored. No one has ever offered to talk about that with me um and so to get some time that specifically about that one issue was invaluable um because its something that affects me 24/7 really um yeah and somehow your made to feel like you cant talk about it so if you know that's why your there just to talk about it it makes it a lot easier. You don't feel you cant bring it up because that's what its about

2: I think I totally agree with that. one of the reasons that I didn't talk about it for so long was um because I felt so much shame and um and when I did admit it the reaction that I got weren’t actually very helpful um and this is the first time I have actually been given the chance to talk about just the voices. and as 3 says it has just such a huge impact on your life because its there all the time and um yeah so I think it's a really good idea.

Researcher: what about other people. Have you got any thoughts on that. if you were going to therapy and being told that we are mainly going to focus on the voices. how does that fit with you?

5: I think it's a good idea yeah. Yeah.

1: again it's the individual. We are all individual really. Sit down spend time, hear the stories. Because I could write a book, I could write a book of my 32 years.

Researcher: maybe you should? Why not? Did you have any thoughts on that, if you went into a therapy and they told you that voices were going to be our main focus how would that sit with you?

20: I think there are other parts of the diagnosis that need to be looked at as well. Everything seems to be voices. you got paranoia, anxiety, depression. I think there is lots more than just voices

Researcher: that's a really good point. Are there any other points that people had?

3: I think maybe picking up on that it's a really really good point but um maybe its important that for somebody to engage with the project that they have support in place that they can take those other things so that they know that this the time is you know what I mean, this time in the therapy is for voices but that they have got you know a care coordinator or some other professional person that's there for all the other things as well so that their not, its not looked at completely as a stand alone issue but it can be related to all the other issues with somebody different

Researcher: so it's the therapist time to talk about voices and then the other parts of your care are for the other

3: and also it enables you to talk about your voices with the other parts of your care because I have never talked to my care coordinator about my voices until I started coming to the voices clinic and then I could and then I have been able to find strands of information that I can feed into what was going on in the other parts of my life you know inform my care coordinator about my voice hearing experience as well as everything else um its kind of tied everything together.

Researcher: one final thing I wanted to ask you about. Say you have had this therapy and there was something that you found helpful what would you want the end of therapy to be like in terms of you working with your care team in terms of going forward to try these things. What would be a good way to end the therapy?

6: theres not a good way to end therapy is there

*laughter*

Researcher: all good things must come to an end

6: yeah.

Researcher: is there a way that we could feed abck to I don't know your care coordinators or your CPNs

6: what about the workbook you know see the progress. Then you could see yeah

RESEARCHER: ok so the workbook to go forward with that. do you think a workbook should be given to your care team so you can see the things that you have been getting on with or is that private

4: I personally want to keep it to myself

3: yeah

2: yeah

3: yeah I wouldn't but um I know that I think I know with the sessions that I had at the beginning a note was made in my clinical record of a very brief outline of what we did um and that was enough to enable my care coordinator that this was an issue and um and then I could choose if and when and what I told her about what we had done. So there was just that link to say you know that you have attended the therapy and maybe for them to have a copy of the structure of therapy so they would know as an overall banner what you had looked at but they wouldn't know anything about how you had looked at those things but it might then enable them to engage in a conversation with you about how you felt it went and what you might like to share

2: I don't know whether it would even be possible to even have a handover between the therapist and your care coordinator with you there and um yeah so the three of you could talk about what you’d so you were sort of more in control of it whereas they write notes about you on the system you have no idea about whats been written and whats on the system whereas if your there in a meeting and um the therapist is handing over and saying we have talked about this that and the other you have more control over whats then going to be followed up or um yeah I don't know if that's possible. That wouldn't really work

Researcher: no that's definitely possible, that's definitely an idea. How does that idea suit other people? If you went through this therapy and then at the end there was a meeting with you and your care coordinator is that something that people would want?

3: I would want the option um because I don't know how I would feel at the end of the course. Whether I would want to or not share stuff. I might feel put on the spot um in that kind of meeting and feel kind of cornered talking about things that I didn't want to and in terms of putting notes on the system I think they shouldn't be personal they would just be an outline of what the structure of a session was without personal details what you said.

Researcher: how do other people feel about that three person meeting?

5: I think that it's a good idea.

Researcher: if you were given the choice would you choose to do that?

5: yeah I would I would choose to do that yeah

Researcher: in terms of questions I have, I have run out. Is there any other points, anything that we haven’t picked up on that have kind of come to people about the book, about the idea of therapy or research study anything at all

3: I cant stop thinking about white rabbits

*laughter*

3: ever since I read that part about white rabbits that's all I can think about is white rabbits

Researcher: did other people read that section. If your told don't think about white rabbits then all you can about it white rabbits

3: its been plaguing me for weeks

*laughter*

Researcher: are there any other thoughts that people had?

3: how do we sign up for this?

*laughter*

Researcher: Anything else at all? Ok I’m going to stop the recording then. Nobody say anything really brilliant now!

## Focus Group 2:

Researcher: what did you think of the book.

8: it was interesting, very interesting yeah. So far interesting., I haven’t read it all yet.

9: what I thought was I thought it was very um it felt kind of completely took in a lot of different viewpoints of peoples experiences of what oi read of the three chapters. I thought it was good.

10: Yeah very good. There were some helpful tips in there as well what I haven’t thought of.

Researcher: were there any specific ones?

10: like coping mechanisms and um

12: I thought the challenging your core beliefs was really interesting um and straight to the point. I have never really thought about that

11: I could actually associate myself with the book it was um like I cant think of the guys name martin I think it was. I could really associate myself with him and the things that it said

Researcher: ok, so is that the case study bit?

11: yeah.

Researcher: did you find having the case study bit in there helpful or was it not necessary

11: it was helpful

9: yeah I was going to say. Yeah I think its on chapter two theres like a test that tells you where you sort of fall in depression and stuff and I found that really useful and I have been thinking about that a lot and um if you get between 17 and 28 out of 30 your pretty sort of normal but you might be sort of um a little bit depressed if you're a 17 and you almost alright if you're a 28 and I got a 14 for it and im on antidepressants and it just made me think a little bit that I might need to push myself because I did the test as they told me to do ti and there was no sort of influence or bias on that so its as it came

12: I think its quite difficult to assess because I got an 11 and im not on any antidepressants. So I got lower than you

Researcher: how did you actually find doing that test?

9: yeah it was quite straightforward

11: can I just say something about the cover?

Researcher: yes

11: privacy is important to me and this cover is quite loud on a bus its hardly coffee shop reading. Its like oh there you go that's what im reading today and its you could be anybody like a professional looking into something I just felt a little uncomfortable with the cover I would want something more anonymous if I was going to take it out and read it just something a little more discrete because if I tell people I have mental health issues that's my business and sometimes I do and sometimes I don't but if a book starts a conversation that's uncomfortable for me then I would find that a little it sort of blows your anonymity a little bit. The covers quite loud

Multiple voices: yeah

12: I didn't like the colour of it actually either. I think it should be like a green because green is a more compassionate colour like green for compassion

9: I um I was thinking about that on the bus yesterday and I was thinking because I was on a packed bus with it and I thought if anyone asks me I will tell them I am a psychologist.

Researcher: that's a really interesting point. Do you think in terms changing the front cover that would be quite a difficult thing to do. What do you think about some kind of sleeve or something to cover it? Would that address the issue for people?

10: yeah

8: yes definitely

11: yeah I found myself with the book in doors that I would turn the book over so nobody could see the front cover because I didn't want my family to see it.

Researcher: that's a really good point

10: my family have seen it and they understand my illness so they got they wouldn't sort of discriminate they are aware but I would say if you are on a bus and if your out in public and people are not aware you can get some nasty comments or discrimination

12: I think that I think that the under title a self help guide to using cognitive behavioural techniques might be something that you want to put in bigger rather than this bit because that would be so obvious. That that underneath bit could be a self guide to using cognitive behavioural techniques could be the big part the big chunk of the book, whereas the overcoming distressing voices could be the smaller in smaller writing.

Researcher: ok so more of a focus on the self help bit and less on the what it is for?

12: yes the cognitive behavioural bit

Researcher: ok that's interesting. Were there any other parts about the content of the book that people really liked?

13: its quite easy to read.

Researcher: in what way? The language or the ways its structured?

13: yeah there wasn't too many difficult words to understand

Researcher: that's really good to know

12: yeah it was easy for me to read I thought the language and it was quite compassionately written I think quite kindly written

14: I like the way at the end you get the actions by scale so I found myself able to identify with it and the things it was saying ... like the hearer distance ... it kind of gets mixed up together it helps with identifying the kind of treatment you might want if you can understand your status according to this book

Researcher: ok so is that the part about your relationships with your voice and how close you are

14: yeah because mine is an outrage really of my inner creators and invasion of privacy although its all happening in my mind and I have to say that other times I feel differently so when I looked at the table I was really able to say ok that's my situation and once you know what your situation is its easier to address

Researcher: that's really helpful

9: there was something regarding the comparison between somebody who um has experience of voices but wont let it get to them and like it would come up and don't go out because of it and then theres another person who kind of distracts herself from it and tries to ignore the voices and I found that quite interesting

13: theres an outcome of that isn’t there? I remember reading that a while back. Didn't one person go on to achieve quite a lot of stuff

9: yeah, well I have got to chapter three so

Researcher: you might have given away the ending there

9: there is a bit in there about um how relationships are influenced as well and um for someone who learns to deal with their voices and their depression is more likely to have successful relationships with people generally and more intimate relationships

10: yeah

13: I think one of them becomes a recluse and the other person didn't

9: yeah that's right

13: the one that challenged it more

9: yeah. But one guy just ended up spiralling down in depression

13: it's a while back since I started the book. I cant really, are those two people based on real people are they?

Researcher: not exactly no but kind of based the experience of the people that have written it people they have worked with in the past

13: right just ideas

12: where was the example of the woman who ignored it

9: yeah I will just try and find it

12: thanks

Researcher: picking up on something that you have said there um the kind of mention of relationships comes up a bit in the book I wondered how you found that because that's not something that always gets discussed when we are talking about voices and I wondered how you found the inclusion of that in the book?

10: well I found im able to talk to not a first a few years back when I started getting voices people didn't understand it in my family um especially my sisters who I fell out with you know they had no they have never suffered with depression or anything like that so to them it was like oh your ill you know. Your ill and its like snap out of it and you cant um but I find with people that I have met friends over the years its helped me a lot because their suffering the same so your not along you know what I mean? So its helped me as a stepping stone to um im not alone something that I can cope with but um I spent quite a few years with my sister didn't understand it believing I was sort of not normal to me its not the case you are normal but your ill. Its getting people to understand that

Researcher: there is a section in the back of the book for carers

14: I read that this morning I read that section actually

12: I thought that was an interesting section because you know the people that are closest to you how it can effect... because I always felt really guilty about the effect im having on my family and what my mental health is doing to them you know and so I didn't read the whole book I read the section on carers because it thought it was good to hear their side of the story and how they felt and if there was anything in there that could shed some light into like whats going on in their experience of it so I appreciate having that chapter in there

10: but like a year ago my sisters came back to me and sort of wanted to get back in a relationship but they had actually read up about it and were understanding of it and so it took like three years for them to understand

Researcher: that's really helpful. So the relationships bit if im understanding you correctly that's quite an important part of whats going on for you, so how did the content in the book about relationships I don't if anybody got to that part or noticed that as they were reading how did you find that section

8: I haven’t got that far yet

14: very helpful for me. Um basically it explains to you that your responses to other people can be um assertive or non-assertive or regressive without you even realising which is what interrupts your life a bit it interrupts your interaction. So what he was suggesting was to practice different responses to simple questions or situations um and put emphasis on the fact that if you have a terrible relationship with your voices you most probably going to be aggressive towards other people. I personally at the very beginning, which was a few years ago actually couldn't stand the sound of a voice that's how bad it was i had so much going on in my mind I couldn't speak on the phone it was just another voice to me although it belonged to somebody dear I just couldn't stand the actual sound of a voice and that had a tremendous impact on my loneliness because you know I just kept conversations short and spent a lot of time with the voices. this helps you well it helped me to realise that it takes a little work to regain your faith in other people and then in you its all down to the way you reply to them even verbally so if you improve your relationship with your voices you will then improve your relationship with the outside world

Researcher: how does that sit with you?

14: I don't see how im going to improve my relationship with my voice because we are fighting literally to be in the same body so I could never be welcoming to them ever ... I don't have that relationship with other people I don't feel that they are meaning me harm or trying to take something away from me so the fact that you still the fact I have this huge distraction what is whats happening in my mind means I can be a little bit abrupt and not friendly to people or not completely understand their point of so you have to work at it a bit more

13: did it recommend how you could improve the relationship with the voices did it?

14: yes by being more assertive

13: what like fighting back?

14: yeah well so the fighting back, I don't really its not really a suggestion I don't really recommended it either its more a question of being assertive back to your voices

12: do you mean when you say assertive assertive as in when you speak or when you think when we are talking about working with voices are we talking about the way that we react towards them in our minds what we think towards them or it's the way on the outside the way we present ourselves because for me with my personal experience where I really really struggle with any sort of therapy is because um my medication has always silenced my own thoughts so I cant work from the inside I cant stand up for myself from the inside I cant create a thought pattern that is going to change the voices because I cant even have a thought pattern because of the medication makes it so quiet I can hardly hear my own thoughts so how am I going to work with the voices when I haven’t even got my own voice to work with that's what Im saying

Researcher: so in the book it kind of suggests you could do it in a very behavioural way so kind of writing things down almost like affirmations in a way or saying things out loud vocalising it or if you felt it was more helpful you could do it having recurrent thoughts but more assertive thoughts instead if that makes sense

12: ok thanks

Researcher: I just wondered was there anything that you didn't like about the book

12: quite a few things actually um yeah I thought that they didn't really mention um it says a self help guide using cognitive behavioural techniques but im not sure if this will be relevant but I did think that they mentioned sort of apart form the cognitive behavioural techniques other ways in which you can deal with distressing voices like healthy eating taking vitamins like holistic therapies um you know all sorts of things that are quite beneficial to mental health but I don't know if that's going to be relevant if they wanted to put a chapter in there about other ways to help mental health rather than working with it in but it might just be a bit about cognitive behaviour therapy then if it is that's fair enough um and also there was a page where on page 25 it said if your feeling as if life is not worth living you should seek professional help from your GP or mental health practitioner or A&E from my experience I had tried well I have wanted to commit suicide a few times and I have never received any help I have contacted a&E I have contacted my mental health worker and they have done absolutely 20 shit about it so there are some points in here where its like oh if you feel like you need talking therapy then please just ask your GP. I have waited six years to get talking therapy so its not as easy as it says that it is in here about certain things

10: I agree with that

12: that's all I wanted to say

Researcher: that's a really good point. Thank you. I think that's one of the things that was discussed in the last group that we ran about maybe some things in the book being too simple.

12: yes

10: its not that easy to get the help you need especially today.

Researcher: was there anything else that people didn't really like about the book?

13: there was something that I picked out was um about negative core beliefs I only read one chapter recently and it was the important thing to remember about negative core beliefs is in fact theyre rarely true and then it gave some examples of some negative core beliefs and its one of them was I am weak I am vulnerable but that could be true about someone

10: yeah

Researcher: yeah I think what the negative core beliefs is trying to get at is that people tend to have this negative view of themselves so if you ask people to list things that theyre not very good at or negative things about themselves people find that a lot easier then if you ask them to talk about their good points so people can reel off all these negative core beliefs and nine times out of ten their not true because what is important about negative core belief it is supposed to be something that is true and stable over time. So there might be periods of your life where you are feeling weak and you are feeling vulnerable but that doesn't mean you are a weak person or you are a vulnerable person does that make sense? So that's why

13: but you could be a vulnerable person you could have that negative belief about yourself and that could be true

Researcher: well yeah that could there is always that possibility

12: also you run the risk of lying to yourself as well like I mean theres so much you could go and say about positive affirmations and telling you to think oh I feel really good today just to make yourself feel good but really deep down you don't theres a risk in that a real risk in lying to yourself and not admitting that you actually feel crap today and the books telling you maybe you should try and challenge that core belief and actually you might feel good I think if your lying certain feelings you can start hiding them and that can be quite scary because I went after reading that exercise in the book and it told me to challenge core beliefs I said to myself I don't hear voices anymore and I went around thinking I don't hear voices just to challenge the idea and its really risky for me to do it. It's a lie

Researcher: I think in terms of challenging the core beliefs it would be something like I don't hear voices but um I don't know you might have a belief about yourself that your not a very friendly person or you're a bad person so I think the idea is that you would challenge that kind of core belief where actually how many people are there in the world that are bad people very few people are inherently bad people so it's the idea that we can have these negative views of ourselves and they might not actually be true its true what your saying they might true you might have negative beliefs and they might be true but to have loads of negative core beliefs is not really

13: yeah like the negative spiral yeah

Researcher: yeah I think that's what its trying to get out

14: to have these opinions about yourself can influence your behaviour and because your behaviour is being controlled by this negative core beliefs that's why if you change those or try to evaluate them then you have a different relationship with your voices I mean if voices say to you um youre a weak person your no good, you could challenge them in an assertive way and reply even if you speak out loud and say im not weak. But to do that you would have to overcome your own opinion of yourself which is that I am weak and they are right. What they are asking you to do is to have a good look at yourself and realise that you can challenge those beliefs about yourself they don't have to be true forever

10: what I do if I get a voice saying you cant do this I turn around and say yes I can.

Researcher: yeah that's basically the idea behind this you might have these beliefs and what the book is trying to do is give you ways to challenge those but I wondered how you mentioned you do try and do that I wondered how other people would find that experience maybe trying to challenge some of these beliefs does that seem like the sort of thing that you would want to do, would like to do or is that a no go area?

10: I mean there were times when I didn't turn it around. I have learned coping mechanisms. I will say oh yes I can which has given me a bit of strength.

8: I turn it around to a more positive yeah

*speaking over each other*

10: I have had to learn that

Researcher: so this is something that you both are doing. How do actually find that?

8: quite hard sometimes but its enjoyable

10: its enjoyable to say to them your not going to end me your not going to beat me.

9: with the core beliefs I have noticed when I do mindfulness a lot of the time that I end up sitting and thinking about it and um in terms of what do I think about myself and what is my opinion on something and then I will sort of automatically challenge why I think that about something so I have er been thinking about the beliefs

12: yeah mindfulness is really interesting and I have found mindfulness really helpful. I have done three courses in mindfulness

Researcher: there is a part in the book I think it comes in in the part about the self when it talks about acceptance and that acceptance is trying to bring a bit of mindfulness into the book. Was there anything else that people didn't really like at all

11: for me I kind of see, I don't, the relationship with voices and giving the voices an entity other than something you would have a relationship with I didn't really identify with but that's very personal nark for me about what was going on in the book because I don't give the voices an entity when I hear auditory hallucinations or sounds or voices or things I don't give them an entity or a relationship or a personality or anything because I see it as my own brain working too hard so I very much accept that its just my brain. I wouldn't give it a personality or I know that's very difficult but that's just personal my experiences not everybodys experience but for me it was that you would have a relationship with something that is your own you know what I mean? I don't kind of fragment that its just my brain the synapses ticking over too quick as far as im concerned my brain is working too hard and the medication slows it down so you know I can cope um the other thing about the core beliefs I wanted to say I had a lot of criticism in my youth and I know that I can self criticise myself very easy and that negative stuff is almost like it started off with some critical parenting and some abusive behaviours in my youth I now then do that to myself quite easily I have taken on that I can criticise belittle be negative have negative core beliefs for myself its like you can put the bat down but I will carry on beating myself type thing so this was really interesting to kind of challenge that negativity around that. But as for voices having a relationship with that I didn't I don't follow that because I just see it as its just me its my head you know so but that's the way I sort of see it. Chemically the synapses are just clicking over

Researcher: so having that kind of understanding of voices it seems like the parts of the book that talked about the self that could be helpful but the other parts not so helpful for you?

11: yeah not so well as in im telling myself on a daily basis and I continue to resend that information to myself as in what I will, what I, like you said we can have a day where we do 90% productive and good day where we have just gone about our business and its all been fine but the 10% that I do that I feel like I can criticise myself for I will. So I will forget about the 90% that happened that actually I was loving and kind towards people and I was good with my family or whatever I will forget up about that and I will beat myself up about the 10% all the time I will go no but you did that bit shit just that little bit of the day and I think challenging that is really interesting and the core belief stuff is really interesting. But when it came to the way it talks about having a relationships with the voices I kind of didn't identify with that.

Researcher: so that's where it kind of lost you?

11: that's where is kind of lost me a little bit because that's not my experience and its just that's just my experience I understand that not everybodys

Researcher: no that's a really interesting point I wondered if anybody else didn't identify with that idea of having a relationship with their voice

12: yeah me. Im exactly the same as you. I wasn't going to say anything because I feel like I have said enough already and I get embarrassed because I cant think straight so im sorry um but yeah um I have the same experience as this lady here, exactly the same. I have never felt that they have any sort of reference to anything whatsoever and they are just my mind playing tricks on me and that um it happens most when im tired when im overthinking about something um and medication yeah slows my brain and helps me calm down a bit so that's the way I see it over brain activity actually and even though they do have certain characters to them like they the voices I hear are everyone in everyday life like everyone I come across turns into a voice and some voices are stable and have been with me for 10 15 years from when I was a child um I don't I definitely don't believe that they are real or that theyre just that sort of echoes that seem to just repeat patterns in my brain its like ive thought about them too much and listen to them too much and they've just got stuck I don't sort of separate them into like what would you say like try and think about them and sort of like a type of relationships like ive got to try and have a relationship with the voices and their sort of a different character than me I think its me doing it so um I said what id like to say

9: when I first started to do something about voices I didn't want to talk to anyone about it and I still kind of feel like its better to keep it to myself outside of this building and er the thing I was going to say about that was recently when you talk to someone who has no idea of voices then you have to do a lot of explaining and you obviously want them to understand it in that particular way as well so its difficult and its far easier not to kind of effect their overall impression of you unless your using it as an excuse for something like talking about it

Researcher: so we have talked about some general things so far. Some good points some bad points I wondered if we could go onto more specifics. I have given you a handout here and what this is is an idea from the research team that has for how we could use this book to base a therapy on. So as you can there are 8 sessions and the idea that I wanted to put forward to you was that we have noticed three main topics within it so we have kind of covered those in our discussion so far. Something about self, something about voices and something about relationships. And I wondered how the kind of plan that we have put forward. What you think of that really?

11: to do an actual group or to not do an actual group?

Researcher: so we saw this as a one-to-one therapy

12: what happens if you don't believe you have a self? I mean its quite difficult in session two like it says beliefs about the self. What happens if you don't think you actually have a personality you don't have anything you feel empty I mean its quite difficult to kind of find something inside of yourself that's yours when your just completely bombarded with voices I mean what is you? When you start challenging asking what do you think about yourself, maybe you don't think anything about yourself anymore

Researcher: do you think maybe the fact that you don't think anything about yourself that theres something in that that could be talked about

12: yeah yeah that's just sort of my experience

Researcher: no that's really helpful thank you

*noise*

10: I don't know if this will help but I spent years up until my early 30s not knowing who I was you know what I mean because id um not, emotional um turmoil in my childhood and um so I was about 30 until I actually felt myself I felt lost and um it wasn't till I went into see a psychiatrist who then diagnosed with psychosis depression um that I actually started to find myself because I thoughts I wasn't a person or I didn't know who I was but finding out the um diagnosis and why I was getting all this negativity and not being positive it gave me a reason to start um believing in myself trying to get the strength to deal with it because I actually knew what it was

Researcher: so you kind of talked about having some difficulties towards of the start and not knowing yourself so how do you think if you went into a therapy and they said this session is going to be about yourself and what you think of you how would that of gone do you think?

10: what before I found myself? I would have been lost

Researcher: that's really interesting. Is that something that other people feel. Are there times when any of these topics would have not been relevant for you?

9: well I think that when your depressed it is um very important how depression effects you and when you ask some questions when your depressed you cant find the answers or you don't want to find the answers and um the other thing I was going to say was triggers theres nothing in here about triggers and I think triggers is really important as well in terms of voices because that can make your experience worse if you don't deal with it so

10: its like my dads voice when I was younger its loud um certain times in my life if I hear certain loud voices it will trigger that off

Researcher: so triggers is quite important. Sorry what were you going to say?

11: I was just going to say when I was going through ... it theres the bit about heatlh eating, yoga the mindfulness theres been times in my life that ive exacerbated my situation and I wouldn't have had any relationship with self um because I was doing too many unhealthy habits and things so although I was quite unwell mentally there was almost a dual disorder ticking along alongside which meant that I wouldn't have had a relationship with self I would have been lost and none of this would have been relevant so I had to address that issue first. So its like you know which comes first the chicken or the egg you have to take care of yourself physically, mentally and spiritually I believe it's a three fold thing um and if your not talking care of yourself to the point where you too ill in one are then almost the cbt is not going to become relevant if your exacerbating your situation already. *interruption* like loads of drugs and alcohol on top when your younger isn’t going to make you feel any better about your relationship with self or have a connection with yourself

9: if your on self destruction there wouldn't be much point in a lot of that you wouldn't take it on board you would be just thinking about where am I going to get my next drink from or where im going to get high

Researcher: ok that's really interesting

11: you know the misspent youth if your young and irresponsible your not going to know a lot about yourself anyway

Researcher: one thing I want to pick up on is this idea of when if we were going to make this into a therapy when would be a good idea to engage with this. And it seems from what your saying that if people are in a really bad place that this wouldn't be a good idea. I wonder if people might want to say a bit more about that

9: I think people sometimes hide the fact that they have addiction or that they do something that is self destructive because they don't want anyone else to know it's a personal thing and sometimes you need someone to sort of analyse them and I always think you know when people go to the doctors and they have blood tests then theres evidence to say they are this or they this and its as simple as that

12: the thing is from my experience about drug addiction and alcohol is that you know I get I sometimes get you know a bit drunk or a bit high or whatever that's because of the medication that im on its just so damn boring its like makes you such a zombie that you just want to feel something going on you just want to a feel a bit dizzy you just want to feel a bit laughable a bit excited you know what I mean? The amount of drugs that they put you on sometimes it just helps you sometimes loosen up. That's what im saying. Its not necessarily always about addiction its sometimes a bit about boredom.

Researcher: how do you think that would work if you were using substances for example do you think you would still be able to engage in a therapy like this

12: I don't think like you should be doing therapy is your on drugs or alcohol its like anything you shouldn't go to work you shouldn't use machinery you need to get help with your addictions first yeah

9: that's right

Researcher: that's really helpful

11: its not the same for everybody I mean its not about addiction some people can use them to have a good time and they don't need to call it addiction its just being normal and most people so relax and unwind

Researcher: I wondered how people felt about I wonder if you can think back to when you first started hearing voices or when you were particularly distressed how would you of found it trying to engage in this therapy

11: I would have found it impossible I was so institutionalised I was so ill um I don't think they did a lot of therapy you did theres a big criticism that you would just sit around drink coffee and smoke cigarettes but actually what could you do when you that mentally ill I think you have to be at a certain level a certain place to be able to engage with therapy in the first place you know wanting some changes and in a place where you are there otherwise you know you just you know you can be ill and need but you don't necessarily have to be ill and want it

12: I mean the help that you get in hospital when I was in there is ridiculous it was like yeah we just sat around smoking and drinking coffee where is this sort of self help this sort of self help book I mean its 15 years on since my mental health problems and it's the first time someone has given me a self help book on cognitive behavioural therapy I mean what happened 15 years ago why haven’t they given me the book in hospital

13: yeah that's a good point

10: yeah I remember when I was 30 I had a counsellor come up once a week that took me back all through my life um which I found helpful but they don't do it now you know that's all gone

Researcher: so it seems like there is a need for this especially in hospitals but if you were in that distressed place do you think you would find it helpful

*talking over each other*

12: well yeah I think you would find it more helpful than you would when you like medicated and less in touch with yourself yeah right in the moment when your feeling it

10: you need someone there

12: yeah you need to talk to someone and from my experience in the most distressing times they stop it they don't do that they wait until your down the line and your calming down and you have forgotten everything and you just become a big mist of cloud its just ridiculous from my experience but

Researcher: that's really helpful thank you

12: its difficult maybe for workers to work with people who are having really extreme thoughts and extreme behaviours but isn’t it like that's when its most awake isn’t it that's when things are most awake aren’t they

Researcher: for me right now its not about would it be difficult for workers that's not whats important about this. Its about when would it be helpful for you that's the point of this group if you would find it helpful early on then that's something we could do

9: I think I was at *a hospital* for a little while and um I think um it could work in there if it was accessible to people because I did a lot of reading in there because you cant go out and your just sort of stuck theres the tv and you can sit outside in the garden but for about six months I just read books and if something like that had been available it might have just speeded things up

10: yeah I agree with that. you don't get the one to one in there help they just leave you to deal with your own

9: they give everyone antipsychotics everyday and that's it

10: theyre not listening to you they just want to shut you up

11: they just give you another diazepam if you go up and say your not feeling too good lets just give you more tablets

10: and that's not the answer

Researcher: do you think something like this could help in that environment

10: definitely

12: yeah

Multiple voices: yeah

12: I think its gentle enough I think its like its not difficult reading its not frightening reading well I didn't think you know facing other people seeing that other people go through similar experiences is very enlightening it makes you feel part of something you not on your own

10: your not on your own

12: it doesn't scare you because your going through something scary as well

11: I definitely would have read it when I was in hospital

Researcher: that's really good to know. Just another kind of thing about the sessions I wondered what you thought about the order. If we were going to go for these three main topics how do you feel about the order. First working with the therapist around the self, and then voices, and then relationships how does that order fit with people.

10: I think its good

Researcher: would you put it in a different way?

13: as its listed here you mean?

Researcher: yeah so you work through the therapy and it would be self voices then relationships

10: therapy to start with I would say

14: looks alright to me id say. The triggers could probably be incorporated um but it doesn't need a whole session on ... which could be quite a good way of explaining the theory and trying to apply hearing voices of a father after a loud voice it can trigger an emotion and that can trigger a voice so that should be included

Researcher: ok so something about triggers

14: yeah

Researcher: yeah definitely, that definitely something we can take forward

14: apart from that its well organised but its um only um doing the core beliefs really and there is much more in the book that's interesting as well

Researcher: yeah so this is quite a superficial list but like you said there are lots of things in the book and what we would like is for there to be that flexibility for you to choose what parts so you might have the topics of the self but you might core beliefs more interesting than the self esteem stuff so you could choose what you wanted to focus on within that topi. If you were going to sign up to a therapy and this is the outline you were given how would you feel about that?

10: it would be good

9: yeah its all important content in there I just think that um a) triggers and b) depressions really important it makes such a difference when your not depressed to how voices effect you in my experience anyway

8: no that's true

10: that's true

Researcher: ok some other things that we might need to include there

10: get the help when you need it not I don't know when you cant deal with it you know what im saying

Researcher: so one of the other ideas that we had was obviously this is a self help book and some of you may have noticed that there are some activities in there so we had the idea of making a workbook to go along side it so that some of the activities you would have multiple copies and you would have short summaries of the chapters so you could have a got at them more than once and have that workbook as your own record of the therapy I wondered what you thought of that idea?

11: it's a good idea

10: yeah a good idea

Researcher: if we were making a workbook is there anything in particular that you think we need to be aware of or anything you would like to see in the workbook.

9: have you seen this one XX?

Researcher: yes I have. So how have you found the stuff that you have been given from the voices clinic?

9: its ok it was quite straight forward really yeah um I found that a lot regarding the exercises a lot of them were a lot of the exercises repeated throughout after each section it was basically how do you feel and how is it effecting you and my opinion didn't really change for most of the group so I didn't fill in most of it it was just the same

Researcher: ok. So that's something we need to think about for this one. The repetition and is that useful yeah definitely is there anything else about the workbooks?

11: I like the idea of being able to come back to it so give several sheets for the activities that your do include rather than having to go back and write them out although the repetition, its just enough that you can go back and revisit it incase it does change you know or if you want a new chat(?)

Researcher: that's a really good point. Ok um that's all on that point. So I kind of wondered if there was anything about the persons situation that you think would be a barrier when engaging in the therapy at all? Can you think of any potential barriers that we might have to anticipate for people

9: well addiction is one init

Researcher: potentially yeah

11: I think high levels of medication in institutions is another as well um you know there were points I was so zonked out on the medication when I was younger I wouldn't have been able to do that

Researcher: I wondered if you had any opinions on how long people have heard voices and then when would be a good idea for this therapy. So would it be a good idea to offer people this therapy right at the very start when they first start hearing voices *interrupted*

10: yes yeah

Researcher: ok.

10: it helps it helps otherwise you are all along you need that

12: I mean these sorts of books should be available in schools as well because like if a kid starts hearing voices that when I started hearing voices as a kid I thought I didn't even know that was something that could happen to anyone I didn't even know that even existed I didn't even realise that was a thing so you know

9: you don't really get psychology in school

10: yeah yeah

Researcher: so you think trying to engage in a therapy like is something that would work at the very early stages of voice hearing?

9: I didn't have any help for about 12 years but as soon as I was diagnosed with a particular illness like in two years I have done three or four courses and I have sort of learnt how to deal with it but I was suffering for a long time before

10: as a child I was having suicidal thoughts and I couldn't tell anyone I didn't even tell my mum it wasn't till later on in life I could actually speak about so I have nothing to no support or it was dealing with it on my own

Researcher: its very interesting to hear that everyone thinks early on would be helpful because actually in the last group that we did the consensus was the reverse that actually having this early on would be to confusing for people because they were still trying to make sense of what voices were so therapy about voices would have been too much so its quite interesting

9: I think that also having it early on because some people are more susceptible to things like suicide and that just having someone to talk to is um sort of proactive and helpful in that respect even if they don't take it all on board they can do it again later cant they I mean there are options to do that stuff

14: you know I think some therapy is important when voices first start but because they start with derogatory and insulting content and you have to give somebody who is just experiencing this for a brief period of time therapy about themselves and how they see themselves it might appear to them either a little off topic or that your blaming them and agreeing with the voices about them being weak you know um and vulnerable and other negative plus the relationship with other people then beginning to suffer basically it would make them feel it was their own fault because I don't believe that the voices are part of me I believe theyre an external thing um but if I was in a therapy where I was told to challenge core beliefs right at the very beginning it might be too difficult

Researcher: so what would you say would be a better thing to start talking about first

14: um well to read the whole book is quite good isn’t it because it introduces the concept of voices how they can be positive and negative if you follow the book exactly how it has been written it would be a very good book to give to someone who is suffering right from the start

10: oh yes

14: whereas if you had to do it from this page um I suppose you do have session one, session two is quite sudden you might want to change you know number two with number six you know and then you're a little bit more in the order of the book

12: so did the first group think that it would make you more confused is that what they said

Researcher: yeah the first group basically said that this particular therapy would have been more helpful when they had kind of got over the first period of voices being brand new and they were kind of into the habit of it more like they knew a bit more about their voices so they said it would be more helpful a bit later on a bit further down the line for them at first it would have been too confusing

12: well its best to ask someone then isn’t it its sounds like there is a difference of opinion so would you like it now or would you like it later

11: I think for me it was misdiagnosed for ten years so it didn't *laughter* I was on the wrong you know so yeah

Researcher: yeah that's really good that we have had that difference of opinion because that shows that theres not a blanket rule for this

12: I mean I think that the doctors are quite fearful I think they have a lot of fear in them and I think that their worried that if they intervene too much at someones most extreme state with any sort of psychology or self help or um you know anything apart from medication then you know they do I don't know I don't know what their going to do but they don't seem to want to do any of that they just want to calm you down and then they that's all they seem to want to do I don't understand what their scared of I don't understand what the doctors are scared of how can you if someones feeling really really extreme and really confused I don't understand why doctors refuse to actually engage with anything that that person is feeling they seem to be scared of being engaging with anything that that person is feeling at their most extreme what are they afraid what is it that they don't want to face with that person they don't want to join in and face with that person its like people who don't have mental health problems are scared even the doctors of facing what the other person is going through and I think that's really sad I think there should be people to stand up and be there for people and say right I am brave enough to go through what that person is going through like someone like XX he did this documentary with this person called XX and he went through her stages with her through her psychotic breakdown through her whole stages he was brave enough to be there for her and not put her on medication and she came out the otherside and she stopped hearing voices and she went back to being a doctor

10: wow

Researcher: he is pretty amazing

12: people aren’t brave enough theyre not brave enough from the other side on the other side of things people don't I mean he he had experience of mental health problems so he knew that he could understand and step up there and be like that because he knew there wasn't anything to be afraid of but a lot of the psychiatrists they theyre oppressing people suppressing people because they don't understand theyre afraid because their on the other side they think theres something they think there something mysterious about it they think theres something that they have to control and that they cant get in there and be above it and work through things with people

9: it might just be the fact that they have got 60 people in the hospital site and they've got two psychologists and they just aint got the time to go through it in that much detail

10: but they should make time

12: yeah its true but even a tiny just even a tiny bit I know what your saying its like theres just too many people

9: what they did do when I was in hospital and I didn't really take advantage of it they did lots of relaxation classes and breathing and that things like that are helpful so there was something

12: so maybe the idea of doing this in a group would be a good idea to cut down on numbers and to cut down on time and cut down on money maybe working through it as a group

Researcher: how do you think that would work as group?

*inaudible comment*

8: I think it would be good

10: because you go and see your psychiatrist and its what twenty minutes and he will say how are you feeling and youll tell him and he’ll say well you know 10 we all get this and we all get that no we don't all suffer with hearing voices and getting told to do stuff we don't want to do so its like their not listening to what your going through and it you know throws you off init

8: yeah

9: theres a broad spectrum of experiences as well I mean um when I was doing the group with 14 there are people in the group who are giving examples of their experiences and I was shocked by some of the things I heard because it varied so much so its very hard to relate to someone because unless they have listened to voices themselves

10: they don't know it

9: no and you know that and its just you feel like your wasting your time sometimes

Researcher: that was kind of one of the reasons that we thought of this as more of an individual therapy because a lot of the things in the book seem to be um topics that could vary quite a lot amongst people and we wondered how that would actually work in a group

12: well at least everyone has an experience of something whereas if you are working with someone who doesn't have an experience of anything that can feel quite alienating and a bit pointless really

Researcher: so would you go for group over individual

12: I would yeah

10: yeah

Researcher: I wonder about other people what would you go for?

8: I would go for a group

10: group

9: I would say group

11: I wouldn't personally just just because I think theres some things that are very private that you wouldn't necessarily say in a group so I wouldn't think it would get to the core issues for me because I wouldn't share it in a group I think privately would help but I think you could offer both couldn't you like you read book and then offer therapy in a group you can come to a group or go do it one to one just this on offer would be good a good idea wouldn't it

Researcher: that's a really good point ok I wondered um if we can think about kind of the therapist in this what would you want from a therapist in this particular therapy

8: understanding

10: understanding of how your feeling and um

9: guidance

10: guidance emotional support

12: recognition

11: fairly qualified stuff as well isn’t it its not just a therapist is it its fairly qualified stuff

10: people who know or have got knowledge of or somebody who has been through it themselves

Researcher: what do you mean hearing voices?

10: yeah

Researcher: ok so someone who has got those qualifications and that experience

12: or has at least done a lot of reading on it

10: yeah

12: is well qualified in the experiences of hearing voices yeah well there is no point talking to someone who doesn't really

10: understand it

9: maybe what you could have is um people that have successfully got over voices that have been to your groups and maybe you could invite them back to speak within the group and therefore people would listen because they would want to know how they did it

Researcher: that's a really interesting idea. So we have kind of thought in terms of this therapy once a week for the kind of course how would that sit with people is that too often

10: no

11: no

13: that's how it should be

Researcher: for an hour?

13: yeah that's it

Researcher: but again that's up for debate again

12: I think an hour

11: I think an hour once a week is ok

Researcher: an hour once a week is ok

10: yeah

Researcher: would that leave enough time in between sessions in terms of looking at the self help book do you think

8: yeah because I haven’t finished it yet

11: I have read it once and I am reading it again because theres bits in it that I have forgotten because I have short term memory loss so yeah I am reading it again and Im finding bits in it I obviously missed the first time around

Researcher: just to pick up on something you said there actually with the memory problems because sometimes the medications or other things that are going on for you can make it quite difficult to retain information is there anything we can do to help you

12: yeah like give us a chapter homework a homework chapter like break it down

10: yeah break it down

12: so like this week we are going to do a chapter and next week we are going to do a chapter so you not like your not like you know your focussing on one small bit rather than the whole book at one time

Researcher: can you think of anything that we could do?

11: cant think off hand

Researcher: ok so that is something I wanted to pick up on actually in terms of the workbooks and things like that if we were going to give you the workbooks would you want the whole workbook at one time or would you want it broken down by chapter

8: I would like it in one go so that I could flick through bits because that's what I have done with that although I have actually read it ive actually flicked through bits and pieces as well so I would like the whole thing so I could see it all from beginning to end

Researcher: really good point what about other people

9: I think when its in booklet form that's um I mean you got to bring it with you every week whereas if you got handouts your more susceptible to losing them and things

14: I think the handouts are good because you've already got the book and then your getting another book and its something you've got to do then its overwhelming if you had to end up course where its *inaudible* you know you got to read the textbook and then fill out the the other one as well whereas if you've got the worksheets every weeks its um it paces better

Researcher: so you prefer individual sheets over a workbook

14: yeah

11: yeah I would prefer individual sheets

Researcher: ok that's really helpful um that was another thing in terms of the therapist we have talked about once a week being an ok frequency I wonder would you want any contact with your therapist between sessions

11: well that would be nice

10: that would be nice

11: but could we afford it?

Researcher: so one suggestion that another study has done is phone calls in between sessions how do you find that

12: I don't want one at all actually because I want to be completely focussed in the therapy groups I wouldn't see any sort of I mean I would be on the phone nattering away to my therapist for no good reason it would be a waste of their time I just want it to be completely focussed for that one hour a week and that would be it and no contact otherwise it just becomes the boundaries of the relationship become crossed and your like oh why am I ringing up my therapist oh because I want a chat and a cup of tea and because I need to sort it out is that because ive got core beliefs or is that because I just need to go and have a cigarette or something you know what I mean you just don't where for me I just wouldnt know where I was at so I would just like to keep it complete in the session and that would be it

9: to have the option to be able to phone someone up or email them might be something just to have that option that you can phone this number and they will get back to you within a day or two if you want to discuss something specifically or your really mixed up about something

11: I think email could be a good idea

14: I mean if the therapist is not involved in the book and the sessions revolving around the book why would you need to see about they are two separate things to me one is going to be working through the book um working through the course and the other one is like your normal relationship with your psychiatrist with the voices clinic you go and vent that's how it was for me um when I did the voices clinic I mean I didn't think that I didn't think of phoning outside of the group for support or advice

Researcher: ok that's interesting so I know in the voices clinic you get um they send texts in terms of reminding you to do your mindfulness practices

14: I never got any

Researcher: you didn't receive any?

14: no I didn't

Researcher: you were supposed to receive texts. How would you find *interrupted*

9: I think I did

12: that would really annoy me if someone was texting me asking me to remind me to do certain practices I would like you know go away you know like you know if I its up to me if im going to do them or not thats my opinion

Researcher: how would other people find texts?

8: I like texts

10: yeah I would find that helpful

8: I would find it helpful

11: I think it would be really important can I just say something I think its really important that if your going to challenge core beliefs and your going to challenge your relationship with your voices and turn things into a positive for me I would value a phone call because sometimes if the stresses are on top it can save a life so I think if your going to offer that as a line of support then it would be important to have that as an option you don't have to phone if you don't want to but sometimes you may need to rather than just want to and that because its kind of if your going into core beliefs its kind of quite its quite heavy therapy isn’t its quite qualified therapy quite heavy therapy so if you want to go in there and have a look then you might need a bit of extra support in the week if you want to obviously you don't phone if you don't want to

Researcher: so it would be good to have that as an option

12: actually I think that's true if the therapy when your working with the therapist and it does trigger a really emotional thing and you go home and suddenly you just cant cope you find yourself you cant cope I think that's a I can see a different side of it now I think you probably might need a follow up email or phone call or something

8: yeah definitely

10: yeah yeah

11: as an option

Researcher: that's really helpful um was there anything else that anybody had kind of around the therapy any ideas or thoughts that they wanted to put forward. How would you feel about if somebody kind of was pitching this therapy to you would you be likely to sign up to it or would you think its not for me

8: I would sign up

10: I would sign up for it

12: I don't think I would bother because um I haven’t got the thinking capabilities to I don’t even know if it's a core belief but I haven’t got the strength of thought to change anything because what we are working with here for me is changing the way that we think about things and I cant even use my thoughts I cant even think properly let alone change my thoughts so for me I think it would be a waste of time

9: the way I look at it if I was to go on a course I would probably find something within the course that would be helpful to me and therefore I would be doing something proactive to deal with my problem

10: yeah

12: I mean I would be interested in seeing how I felt about myself the feelings I have towards myself like um I don't like about myself I don't like about myself but the thing is the fact that it's a cognitive thing wouldn't be helpful to me because I struggle with cognition in general so it would have to be more of a feeling it would have to be more emotional therapy rather than cognitive therapy for me yeah

Researcher: do for you this approach isn’t one that would work for you

12: no it wouldn't work for me no it would have to be totally emotion based if that even is a therapy I couldn't do the whole thinking thing it would work

Researcher: that's really interesting. Is there anybody else that thinks this kind of approach wouldn't work for them?

*silence*

Researcher: is there anything you wanted to add Researcher?

Researcher: no I have just found it all really interesting the way that some of this group is different to the other group on some things and then other things its just interesting to get the two groups and how things are going to work

Researcher: yeah its really interesting its good to have those differences in opinion as well. Is there any kind of advice for us if we were going to take this therapy forward which we are planning to as part of a research study um to try it out to see if it is effective is there any advice you would have for us in terms of designing the therapy or designing the study anything like that

9: um one thing from the voices group that I have done that effected it quite a lot was perhaps that some weeks not many people would turn up and one of the main things I got out of it was listening to the experiences of other people not the actual er content of the groups so maybe to have some sort of restriction on people if they miss more than one session or something just to make it worthwhile for everyone I suppose

Researcher: any other things, any other advice to us going forward

12: yeah some helpful tips on how to remember things as well because a lot is covered in a therapy session I guess and um you know it's a lot take on board so it would be good to find little ways that you could try and remember whats been covered and you know when you do find something that you find that you've overcome or that you come to terms with it that you can have it written down or have your therapist make note of it somewhere and for you to keep it somehow because otherwise it could just be lost I guess

Researcher: so is that something that we could put in some sort of handbook or workbook. Some sort of notes section?

12: something for you to take away after the therapies been done

Researcher: something that got brought up within the other group um it was discussed about workbooks at the end of the session being shared with maybe at the final point with the therapist and with your care coordinator is you would want that shared or not the work that you have been doing as a final session

8: yeah that sounds like a good idea

Researcher: yeah that was something we talked about is how to end the therapy so is this something that you would like to keep between you and the therapist and its you and then up to you to take it back to your care team if you would want to or is that something you would want us to facilitate

11: no it would be good if you(?) did it

9: yeah it might put an unnecessary stress or pressure on someone I would say as in to you must do the homework and you have to explain this to your care worker at the end of the therapy and stuff but having the option to do that I mean if someone wants to help themselves they probably will

Researcher: is it something you think we could help with? To kind of facilitate that discussion between you and your care coordinator or is that something you would want to do on your own

9: yeah I mean maybe before the group even starts to come to an agreement as to whether you want to talk about it afterwards because you will probably know at the start of the group whether you want to share your experiences or comments with someone

Researcher: does anybody else have any opinions on that at all?

8: I would like my care coordinator to know

Researcher: what would be the best way of us doing that

8: well just telling her what the group was about because she likes to be involved in um knowing what other groups are doing so that she can help me because I have just done four weeks of um talking therapy and um she was in on that and she knows that its happened so that she knows the next time she sees me it's a good place to start at

Researcher: would you because you mentioned that they have had a phone call or an email between them. How do you feel about not being a part of that conversation

8: my care coordinator always tells me what they have discussed

Researcher: so for you that's not an issue

8: no

Researcher: ok how would other people feel about that at all

11: well its an issue for me I think therapy is therapy and it stays completely confidential in therapy and that a relationship with my therapist the relationship with my psychiatrist who gives me medication is different and I think I would probably trust my psychiatrist I would probably tell him somethings but not necessarily tell him everything I do in therapy so therapy is therapy and that's a different thing

Researcher: 13 did you have any opinions on that at all so if you come to end of therapy would you want your care coordinator to know about whats happened in the therapy

13: yeah I wouldn't mind some of the stuff yeah it depends what happens really it depends what the outcome of the um course was like um I might decide that there is some stuff I didn't want her to know but I don't know whats going to come out I don't know how successful its going to be

Researcher: so if there was things that you wanted to share that you had agreed with therapist is it as simple as sending an email to your care coordinator or would you want to be a part of that conversation

13: I might be up for that I would have to think about it first

Researcher: that's really interesting. Was there anything else that anyone wanted to put forward at all about the book or about the therapy

12: I was just thinking right now does it go over into ideas of um you know how you know um like whats the word you know like when you hear voices it can also get mixed up into believing like say like your reading other peoples thoughts and things like does the book just deal with the sound of voices or does it actually go over onto other experiences as well

Researcher: so this book is specifically about voices

12: but say if you believe your reading other peoples thoughts that might that's not hearing voices buts it's another sort of type of delusion that it can have maybe a relationship with maybe hearing voices

Researcher: so I suppose you could go over that in relation to voices there is scope to do that in terms of beliefs about voices and beliefs about where they have come from

12: so it would the therapy would go over strange beliefs

13: yeah that's what I was going to ask is it schizophrenia is that would that be a part of it

Researcher: yeah so I suppose that's something else we could discuss if we were going to recruit for this study do you think we should just recruit people that just have kind of psychosis or should we recruit anybody that has experience of hearing voices that

10: anyone

9: otherwise you could potentially close the door on someone which can *interrupted*

10: and that's discrimination

9: yeah

10: you should involve everyone

Researcher: that's my thinking. Its nice to know you agree. Yeah so this book is just on voices but the overcoming is actually a series of book so there are loads of different ones so there is another book for unusual books, so there a book for depression and a book for anxiety and they are supposed to be specific self help books for specific issues

12: I think its nice they have done it in small chunks yeah

Researcher: there are absolutely loads of them. But this is the one we would be focussing on but like you said there would be some overlap

12: with the book they use they could be a bit flexible on maybe overlapping into depression in therapy group even if you are working on distressing voices they would be a bit flexible with that

Researcher: is there anything else that anybody had

9: regarding the books in the hospital um I can think of one other book that I read before I was diagnosed with anything that helped me through a difficult time and its called the road less travelled and its by an author called um [name]

8: I have read that book its really good

9: it's a really good book

8: theres another book after that as well

9: oh right

Researcher: ok um well if that's everything that everyone had to say I just wanted to say to wrap up so firstly thank you.......

## Focus Group 3:

Researcher: Hopefully you have all had a look at the book because that's obviously what we are here to discuss. Just to start off um what did you think of it? That's a kind of open ended question to start on. Does anyone want to start us off?

*interrupting each other*

15: no you go

16: I felt especially the first few chapters were very repetitive they kept asking you to refer back to these two people at the beginning so one minute your trying to concentrate, I don't concentrate very well. You read a paragraph that says as the two people and you have to go back again and then forwards and then back again for quite a few of the chapters. I think it would be quite handy for someone that's just found their hearing voices its just happened to them because a lot of it *interrupted*

15: did you find did you find because I found from my own experiences in life anyway and I had counselling before I generally knew most of it anyway. Did you find that?

16: yeah that's why I suggested it

15: although do you know if with cbt repetitiveness is how its supposed to be because you have got to get yourself into a certain frame of mind

16: yeah

15: and keep thinking the same positive thoughts until it sinks in so

16: yeah so maybe for someone that's been diagnosed or needs that extra bit of help it's a good book for somebody that needs that bit of extra help that's how I saw it

17: I found some of it I was doing already especially the bit about core beliefs um you know when it was saying um that things were from when you were a child and you need to say sort of whether it was true or not I found that I have been doing that anyway

15: so you regress like me. Do you feel like you've regressed in yourself somewhere or you know um

17: yeah I think

15: do you think a lot about certain things in the past which you

17: I can do if im not feeling very well yes then things from the past will come

15: is that quite chaotic for you?

17: yes yes it is

15: I understand that

17: but I found that things like when the voices to me you know your no good nobody likes you and it when I first started getting the voices that's all they did they kept saying the same thing over and over again and it got a bit repetitive and um but over the years they have got more sort of chatty and get more involved in what im doing

15: ok ok so you've changed with them

17: mmmm

15: so it sounds to me like your learning to cope like myself with the voice going on inside of you which is really great rather than to fight it and not get anywhere

17: no because I found trying to argue with the voices it just makes it worse

15: yeah yeah

17: one way I have find helps me to cope is because mine were fairly constant so I might say to them right ive got to do this this morning and I will give you a bit of time this afternoon about say four oclock

15: oh ok that's a good coping mechanism

17: and and then they don't go but they go to the back and then about 4 oclock I will let them have their little little like yeah yeah somehow it just eases them off a bit

15: yeah referring back to this *the book* um I think that I can relate to all of whats in here um which is quite amazing reading it and um there the thing that strikes me the most is the core beliefs is you know its you're your changing your worth

17: yeah

15: you know your having confidence in yourself and changing how you feel and think about you and working on that um I think that's very true and I think that um to be able to have therapy where you can talk to somebody privately about how you feel and what you think and what your going through is a must have and I wish that I had somebody that I could go to and you know its just so expensive now like 45 50 quid a session to go and have counselling and stuff um but I think the cbt, cbt works but I think that the person whose um encompassing that cbt has to do it for a duration perhaps two years three years in order for them to get well again. I mean I like the book very very much. I did find I still have got a little bit left to finish um I found the explanations in the book *shows a page from book* um very good but some of them were quite complicated.

Researcher: do you mean the diagrams?

15: yeah the diagrams I you know for example I found that quite complicated to digest and I had to um try and I had to go over it several times to be able to get it

16: it could be worded a bit easier

15: yeah um yeah it could be um more simpler um but yeah I think yeah I actually think it's a good book and I have read hundreds of books in my life I think it's a very good book though I did also notice that um I have written it down somewhere it only I suppose if it's a first book isn’t it? Um its only it only I find it quite interesting that the only responses discussed are those that are emotional and behavioural what about logical and intellectual response as well as biological and physiological so its not only just the mental side having um schizophrenia effects everything that you do it effects your body it effects how you eat you know sleeping um emotionally intellectually um and I thought that perhaps that should have been mentioned more of that but I suppose that's um going into a more complex form of a book really um but I thought most people they don't when your ill you don't think that um certain parts of you I mean I think about the parts of your brain are working in a totally different way or theres a totally different chemical reaction going on inside yourself and sometimes when I have been by myself and felt really depressed I remind myself that I am depressed and that I have a depletion in a certain chemical like the sun so I will go out and sit in the sun for a bit and that might lift me up a bit you know just stuff like that the more you know the better it is to know because it can help you rather than hinder you you know its how you take it

18: its like music with dopamine listening to music that you like theres more dopamine getting in your brain

15: which again is another coping mechanism its just finding as many coping mechanisms as you possibly can um which I think should be more detailed in here um because that's what we need you know talk about drugs you know alcohol you know chemical drugs which is great that in the long term um you know it doesn't help which I thought was very good that that was mentioned but we need to know more about the therapy side of it

Researcher: that's a really good point. So a long with the book there is a workbook as well. The book itself is published but the workbook is still being so we can include more stuff in the workbook about coping and maybe encouraging people to go and find more information about coping. So that's definitely something we can change

15: oh great. Maybe more support groups I mean this is like I haven’t shared this information with anybody um theres only um two people that know my doctor and my brother and ive seen health therapists and things like that but nobody knows this about myself because its so personal and private to me and I think that having people to talk to about it is really great so thank you for listening to me

16: there are hearing voices groups theres one in Worthing and one in Brighton

Researcher: is there?

16: the one in brighton every week Tuesday

15: too far

16: and theres one in Worthing every first and third Wednesday of the month

17: theres a hearing voices group here actually

Researcher: theres two in Worthing

16: theres two in Worthing well I didn't know that

17: theres one here on a Tuesday at 4 oclock

16: is that every Tuesday?

17: every Tuesday. Its actually been postponed to the 21st because I help to run it and yes um but it starts again on the 21st and its from 4 till 5

15: so do I have to be referred to it or

17: what you need to do, I think *staff member* is in today just have a word with her and she will have a chat with you

16: the one at *place name* you don't have to be referred to you just turn up

15: right its like a drop in

16: for that one yes we go to that one

15: oh right

16: its alright good bunch of people um

19: you never know how many people are going to turn up do you

16: yeah

19: sometimes its five sometimes

16: its nice because you can pick out so many different coping mechanisms and strategies and stuff that I wouldn't personally think about myself so that's why I think that book would be good for what I call starter outers

*laughter*

Researcher: the newbies? Ok

16: because if I wake up one morning well I did and then started hearing things I would want as much info as I could because when I first heard it its was just all gone I didn't have it

17: I tried to deny it no Im not hearing this no no

16: I thought I had a super sense like superman and I can hear people

15: that's the delusion part of your psychosis

16: oh god and then they started calling my name out

Researcher: you have kind of mentioned some positive things so far. Is there anything that you didn't like about the book?

16: yeah just what I said about you having to go back and forth to chapters I think it was done too many times

17: like the lady said about the diagrams they were a bit difficult to get your head around them

15: I think the um headings are very good so like when your reading through and its titled the paragraphs are titled so you know when you re read it again I remembered I could go back through and remember that part that's a positive that's a good positive

16: and it was bold and I quite like that because I am as blind as a bat

*laughter*

Researcher: was there anything you wanted to share?

18: I think one thing well what negative or positive?

Researcher: whatever you like

18: well one thing positive one thing is having relationship with *voices* because it is a relationship its not going to suddenly go away so the whole denying it and stuff

17: its not going to work

18: it will just become worse that's when it will be uncontrollable so I think having a relationship with them and maybe not talking to them but giving them as you were saying some leeway like an actual amount of time for them to do what they want um that what I try to do and sort of yeah give them some attention sometimes but also try and get on with your life and leave them sort of sort of I don't know realise themselves as if their real people realise themselves

15: so what your saying is that its its helping the book helps you to understand your experience?

18: yeah understand my beliefs

15: understand what your going through

18: yeah

15: yeah with whats happening to you

18: yeah

15: yes that's true

18: um negative um I think there was one bit oh and the fact that everyones experiences are going to be different and everyones feelings and attitudes towards it are going to be different so I think one thing about the book is that it just gives one kind of view

16: it generalises it too much

18: yeah that's it generalises it just a little bit whereas everyone is going to have their own experiences so they should sort of put that in there your going to have your own coping strategies and they might work for someone else and they might not for you

Researcher: that's a really good point and that's something that has come up in the other groups that we have had that they didn't necessarily relate with them and this is where the idea of the workbook came up so the idea that you could basically almost have a copy of this book but it would be like bringing the book to you using your story. How does that sound to people as a way of addressing that issue? Does that sound like that's enough?

16: so the book becomes like personalised?

Researcher: yeah.

16: yeah that's good

18: yeah sounds good

17: I think as you say everyone is different for me I do find writing helps so writing something down in a book would be good for me because I can look back at it and I would be able to deal with it better by writing it down I know everyone is different but I even if I am having a bad time when they say to me oh nobody likes you or whatever and I will get out a book and write down so and so likes me you know just to try and

16: give yourself some positive feedback

17: yeah yeah

Researcher: so something like the workbook that would work for you?

17: yeah yeah that would be something that I would like yes yeah

Researcher: what about you Abe?

19: yeah yeah I like that its been a while since I read the book so I had to write a few notes while you were talking. There is some really lovely phraseology in there from ruth’s story being too noise sensitive to leave the house and too scared to stay there. I really empathise with that. I think I have mentioned it many people as old as me that have been hearing voices keep it to themselves for a very good reason you would get committed to an institution also hearing voices from an early age is very difficult to ignore the commands just to not take them literally and go burn something go and destroy something always there always very difficult and I quickly learned as I was going into my teens that um that I cant do this anymore I have to take a sort of different view of what the voices are telling me its *inaudible* it also briefly mentions the use of alcohol as a means of get a rest from voices but if you dare to tell psychiatrists these days that this is the case the confuse cause and effect and they always tell you that hear voices because you're an alcoholic and its absolutely not true I didn't drink when I was six years old and I heard voices *inaudible* theres the description of a panic attack which I have also and also medication its speaks a lot of and antipsychotics I really haven’t found thing antipsychotic wise to help me I am lucky enough to be prescribed some lorazepam which for when I feel these panic attacks I am able to take the lorazepam which is far better than any kind of um but antipsychotics seem to dumb you down and your walking around like a zombie that's the best effect they can have with those. The self esteem tables I completed the self esteem tables and I was only able to score 16 um perhaps im not saying it did but it seemed to me perhaps suggesting that I hear voices because I hear voices and I believe it is the other way round I believe have low self esteem because I hear voices before I could hear voices I was the most gregarious child you could meet you know um so I did complete all the tables and whatever

15: I think its quite amazing I think your quite special to have experienced voices for such a long period of time and still be here I think that's amazing so well done you that's pretty amazing

19: lots of the medication exercises obviously certain techniques are mentioned all of which tend not to work for me something to do with this ... causes the voices to be worse than ever and um I thought the section about changing beliefs about voices was good well written not unlike some of the work I am trying to do with *support worker* um I scored fairly low on those tables I score 16 I thought the whole chapter was really good. But my core beliefs are old and really old and I think I would need some professional help to change that but the tables were particularly helpful um I have no criticism of the book. It says that hearing voices in itself is not a problem but I cant agree with that because hearing voices itself is a problem even if the voices are being nasty or not saying anything to you I have a terrible job trying to listen to two people at one time yeah so hearing the voice even if its not being scary or disruptive it's a matter of I don't want it to speak to me I just want to concentrate

17: yeah I find that. what I also find distressing is if im talking to someone im seeing my support worker and im talking about my voices the voices don't like me talking to people about them so they will say things like tell *name* to f off and I hate that because I

15: that's not how you really feel

17: that's not me you know

15: its going against all of who you are

17: and I actually told her because she could see there was something distressing me so I said sorry *name* but they are telling me to tell you to f off

15: but that's really good that you can tell somebody

17: yeah

15: so they understand whats going on in you

17: understood yeah and she said to me she said I will go when you want me to not when they want me to go

15: oh that's great that's really great

19: I haven’t built up the support network to be able to do that I find it terribly

15: maybe that's something you can do

19: I find terribly embarrassing infront of my daughter especially when she was younger in a queue or in a crowded place where I just cant be but I have to get out of there quickly and I would do something you know bash into some ladies trolley just to get I needed to get out

15: so you find yours very distressing

19: I do yes even if the voices is not being particularly distressing but its is and its impossible

15: so you must suffer quite severely from anxiety

19: I do I do i have ongoing nightmares

15: do you think its like a catch 22 I think its all related isn’t it

19: yeah and I have just one question its says theres no scientific evidence for this but um I once was told by a professional that you can see in a CT scan the voice hearing part lighting up

15: yes you can yes your right your absolutely correct

19: and I was interested in that I don't there are any means of like but it's a start

15: its shows that theres more brain activity in parts of the brain where it shouldn't be then there is when it should be does that make sense

Researcher: yes

15: which there is I mean you fight to have private counselling you know have someone say this is the prognosis your going to get well we are going to do this this this this I would do that but that's not a reality of the situation for anybody I don't think

19: I just have one more thing to say about the book not always but in lots of the book they say we all have a two way conversation with the voice I actually don't I can shout scream speak at the voice and they will speak at me but we don't have a conversation one to one conversation the voice will eventually answer or pass opinions but it wont be immediate its very very rarely immediate

15: so do you think that happens more when you relax its comes more to forefront when you relax and when that does happen you start to get anxious again

19: that's right

15: I understand that

19: I understand that many people have a regular two way conversation

15: I have that

19: do you?

15: yeah I have had situations where ive been in my lounge trying to watch the television having to deal with that then they trying to talk to like a past boyfriend at the same time whilst voices are going on in my head I mean how I coped I mean there was times when I just wanted it to it makes you angry and it makes me feel frustrated and theres been moments when I would just like to go argh! But you cant do that although I have done it where I live once and they all think im mad but I don't care um but yeah its not nice feeling out of control and being empowered by certain arguments with voices that are going on in your own brain because this is this belongs to me and I often feel that im being infiltrated all the time you know um and I do I realise now from the experience that you know in a medical world what we experience is called schizophrenia but in the spiritual world its called Claire-audio. And I find that quite interesting you know that I used to go to a spiritual group and they used to welcome this um you know voice hearing and stuff like that and you could talk about that if you wanted to um but I wouldn't I would not recommend doing that because it can make the situation much worse so if you can try and you know keep more to the medical side of things I think its better

19: well your giving more grievance to the voices aren’t you?

15: yeah exactly exactly.

17: I mean what I have always found interesting was when I hear the voices and I say that I feel depressed people think its because im hearing voices I am depressed but its not when Im feeling really depressed its what makes the voices worse because I cant fight them off I haven’t got the energy

18: the willpower

17: the willpower to do it

15: its like they overtake don't they they take over and that's something also that could be mentioned as well is I mean I am on psychotic drugs um which obvioiusly dull the voices down they kind of work but they don't

16: you’ll never get rid of it

15: yeah I know um and to have a bit more information on medication would be really good you know explaining what types of drugs are doing what type of thing that would be a good thing I think because I don't I only have a limited amount of knowledge and it would be im such an avid book reader I like to pick things up and find as much research as I can on the particular subject so that I know I know what it is going on with me and I know what I am talking about but that's kind of what I am like but I think that would be good

Researcher: is that what other people think? Is there a place for this to have more information on medication?

17: medication yeah

18: I think all areas. Not just the medical you have everything else as well. It would allow people to have a choice on what their experiencing and what they feel like they would be better to go and do

15: yeah gives more of a choice of how they want to deal with it

17: what their options are

15: yeah you know also more support groups and stuff like that would be a good you know details on what services you could go to if this is happening to you rather than having to go to your GP and you know go through things that way you know I have gone to the book shop and bought many self help books and you know at the back they give you details of which places you can go to get counselling for that specified genre you know of whatever it is I think that would be a great idea

Researcher: yeah that's something we can do

16: whats that thing you said about that chemical in the body?

18: dopamine

16: dopamine something like a chapter on natural products instead of tablets all the time

18: like ginseng

16: yeah im on about natural like like mediation music I think raising your dopamine level in your body naturally is not for everybody obviously is just as good as some medications but you have to stick at it you wont get it in a week or a day all the stuff that I have used to help me get through the night I have been plodding along with that for weeks and I find after a few days it gets a bit better and then another few days it gets better but it's a hell of a lot nicer than taking tablets

15: so what type of music do you listen to then?

16: its all meditation music

15: oh that's beautiful

16: its about 8 hours long some of them I don't listen to it for 8 hours but what I generally do is I do a non smoking hypnotherapy um dvd on youtube um that's when I was smoking so that I didn't have a cigarette before bed and then I do meditation and relaxation and this women talks and tells you how to calm down all the energy blah blah blah and again I didn't batter an eye lid when I first you know im not doing that rubbish I’ll just pop a pill but its perseverance you will always find something that helps you

15: alternative therapy

16: yeah

15: alternative therapy

18: mine was sport

15: sport is excellent

18: sport was mine like it I would just be waiting to play sport and that's the one time I wouldn't hear anything at all and I would be there for hours on end because I wouldn't hear anything at all and that would be like the one moment when I actually like

15: did you feel good about yourself

18: oh yeah yeah definitely that's the one

15: that's your road

18: yeah and that what I yeah other coping strategies one of mine one thing they tell me to do is cut myself and I had a big problem with self harm and that sort of stuff

16: yeah I went through that and all

18: so I found one way of doing it is not to deny like no im not going to cut myself is to get a pen and actually do it with a pen just drawing lines on your skin instead so its psychological in the fact that they get something from it because you have don't but at the same time you haven’t caused harm to yourself

15: yeah I think that's genius

18: yeah

15: I think I might do that

16: mine was to relieve depression

18: yeah

16: from the voices but as soon as I did it I don't like admitting it it worked

18: it does for a little bit

16: and I did it what 2002 and I did it about 5 weeks ago where I am at the moment and my friend came round and she told me off and she took my little pen knife away um

15: was it triggered by anything

16: night after night with no sleep

15: oh

16: and I had had enough. The pills weren’t working the doctor wasn't listening and then I had a flashback to when I was in a psychiatric unit when it worked and I just picked it up and before I knew it I had done it it wasn't anywhere near as bad as it was then it was just so I could see blood but that's a good idea the pen especially a red pen

18: yeah exactly a red pen

16: that's a good idea actually because if you put its not so much the pain it's the visual

15: the act of doing it isn’t it

16: yeah

15: yeah

19: but sport also as you mentioned works. I used to climb rocks rock climbing back in when I was a young man back in *place name* but um im old now

15: old-er

*laughter*

19: I cant even climb a staircase now

18: do you know *place name* that place is heaven for me I will walk up there by myself and because your in nature it's a lot more open you know you can have a conversation with them because no one is looking at you so you do have time with them as well but also it's a lot more open you feel more I don't know less constricting

15: that place is amazing. Less oppressed

18: yeah exactly yeah so its just I love walks. Walks are the one thing that's why I really want a dog. Because it's another companion to walk with

19: can you have a dog?

18: yeah I can. But

15: its quite a big responsibility weigh up the pros and cons before you actually you know take a dog on board

16: I don't know if anyone else has tried it but I was told about it. If your out in public and your telling your voices to bugger off just put your mobile phone to your ear

*laughter*

17: yeah it looks like

16: I always worry that you do that and then your phone rings

*laughter*

16: so I didn't do that one

Researcher: I wonder if in the packs that I have given you. One of the forms its called session ideas. Its this one that's got like the table. We had the idea of using this book to base a therapy around so giving people the book so they can engage in some level of self help but also having a therapist there to support them through it. Because some of you have picked up on the fact that some of the diagrams are a bit challenging or difficult to understand so if you had the time with the therapist hopefully you would be able to talk about things like this so this is the kind of therapy schedule that we have come up with so hopefully you can see from this that there would be 8 sessions and there are three kind of main topics that we picked out from the book so thinking about the self that relates to the core beliefs that a few of you have mentioned, beliefs about voices which I think you mentioned 19 and relationships which you mentioned Ed. Um so I just wondered what do you think about these kind of topics do they seem like they would be important? Whats not so important? And what order would it make sense for you think about them in the therapy?

16: you mean to say eight sessions with someone

15: so this is session one is it?

Researcher: this isn’t the therapy

16: this is the summary

15: oh right yes as if it was the therapy

16: eight sessions I would be too oh ive only got 8 sessions to get through it

15: immediately you would back off it because you would think oh ive got eight sessions and I have been having this for years you know eight sessions isn’t going to you know

16: its just the numbering if you just put sessions down it's the numbers if I came to see someone about whats happening and they passed me this I would be like wow session four I might be able to talk about that one and then ive only got so many left to talk about the other ones you know what I mean it's a bit. You got only 8 sessions it's a bit harsh

17: and you get to session six and you think nothings changed

*laughter*

Researcher: we can make lots of changes to this therapy but it would have to be eight sessions that's the only thing we have to stick to

18: I think the session two beliefs about your self and how you start to think about any negative beliefs about yourself I think that should be brought up later on rather than at the beginning because it might um if your talking about with someone for an hour about how your negative and this this this it might effect you in the whole rest of the session whereas if you did it towards the end once you have learned your beliefs the coping strategies you have done so you can actually start looking at yourself now and think what are the negative things so it wont be as I don't know maybe that's just my opinion

Researcher: no that's a good point so coping seems to be an important thing to come up early on

15: sorry so this is the strategy of how the book would be relayed are you just asking which is whats the best session to put first

Researcher: so what would be the best topic to talk about first in therapy so would it be talking about yourself talking about voices or talking about relationships what would make sense what would be easiest for you to talk about first

17: I think myself first about how I was feeling and what I was going through I think that would be

16: yeah the voices are a close second because your there for your voices to understand it and gain more knowledge about it im a firm believer in putting it all on the table and not pussy footing around you know because sometimes that's shock therapy but saying it as it is sometimes is better than going around the gardens you know

17: the only thing is if its like that and your in a session when the session finishes I think about it afterwards and then im on my own with it

18: yeah

17: its almost

18: touch and go

17: yeah

16: that's why it is good to have a come down period of ten minutes so say you had a mad session you do an intense half hour and 45 minutes of yes you've got voices how does it feel but and then maybe just I don't know talk about something else come off the subject slowly

17: yeah

16: so it doesn't seem so

15: it's the same type of thing as if you know your cooling down from an activity it's the same that's a good point that's a good point

Researcher: yes so the people you would be working with are kind of trained therapists so it wouldn't be a case of them getting you to talk about all this negative stuff then saying see you next week um

16: theres plenty of trained therapists out there that do they listen to you and then as soon as your time is up they look at their watch they are out there

15: yeah yeah

*talking over each other*

15: I went to go to a cognitive behavioural therapist when all these voices started happening I was so distraught and so distressed and er I you know booked a firs trial session and he wanted a £135 an hour and what had happened was I went to go over to see him and I got the wrong address so I turned up initially at one place and when I did turn up which was an hour later and I knocked on the door and I said oh im really sorry you know I got the address wrong he told me to clear off and not to waste his time and I was just so taken aback by that so your right they are out there money grabbing

Researcher: I imaging those working in the NHS aren’t in it for the money if not then they are working for the wrong company

15: oh gosh no

Researcher: so obviously this will be within the NHS with NHS clinicians that have worked on our trials before but the idea of a cooling off period

16: even if its just like a drink you know and how are you not how is your head but how are you what have you been up to you know have you been out you know so you don't begin the session with voices and end the session with goodbye voices because that is its surprising what ten minutes of not talking about it can set you up for when you leave

Researcher: how would that sound to other people if at the end of the therapy session you had the choice to have that cooling off period

16: yes exactly I would say everybody is different I prefer I can talk for about half an hour 45 minutes solely about what has happened to me and night time but after that im ibe had enough

15: yeah theres only so much you can say about yourself

16: and its nice to get to know not to get to know the therapist but because your pouring out your heart to the therapist just a like a little connection you know what I mean

18: Yeah

15: yeah yeah

16: some people are different some people like the fact that you don't know the person you just say ta-lah if im talking to someone about private stuff I want

17: to know them

16: yeah you know what I mean not so much about them but

17: to feel comfortable with them

16: yeah that's it

15: trusting isn’t it to learn to trust

16: I don't trust many people that takes a long time

Researcher: that's a really good idea and that's something we can do really easily is to offer people that if they want that at the end a little cooling off period

16: yeah a little cooling off period 20 daniels and coke

*laughter*

15: would it be ok if I had a glad of water please im really parched

16: its really really hot

Me; so we have talked about the kind of topics and you mentioned that self would make sense for you

17: yes yeah

Researcher: whereas you would prefer to get straight into the voices have I understood you correctly

16: yeah I think it needs to be up there at the beginning of the meeting session one session one discussing what was going to happen or what you would like to happen

15: yeah that's a good idea

16: so maybe its not set in stone sessions one being *interferance* how do you feel what do you want to do some people will have stuff they want to get off their chests really

19: to incorporate into the introduction as well

Researcher: ok so within the introduction how would it be if it seems that coping is an important thing how would it be if in the first session if you wanted to you could talk about voices but you could talk about coping strategies does that sound like a good compromise and then you could into self and then back to voices in a bit more detail when your feeling a bit more

17: yeah

Researcher: comfortable

15: I think relationships should go last

Researcher: relationships last ok

15: definitely it should be touched upon

16: is that the relationship with just the voices isn’t it

Researcher: um with that theres kind of the option so you could look at relationships

15: in your social world with other people and your relationships with individuals

16: so there are social yeah

Researcher: whatever would make sense to or be helpful to you. So relationships going last how does that suit other people would that make sense to you Ed?

18: um yeah

Researcher: cheers ok so we have talked about how it might look and I mentioned to you that we would be putting together a workbook so this is quite a practical question how would it make sense to you for us to give you the workbook would you like it in modules so you kind of have like

16: not an NVQ again

Researcher: not an NVQ don't worry so kind of like we are going to talk about the self so heres the self book or would you prefer to have it all together upfront like you have with the book what would be easier for you

16: personally all upfront

15: yeah

17: yeah I think I would rather have it all together you know

19: same here

Researcher: I wondered if that would be intimidating for people

15: or too intense like too much information for somebody to feel like they cope could with yeah but a book is almost like a library

16: you can always close it

15: yeah exactly I can go to whichever part I want and go over it again um yeah you know

16: depends what you class as too much is it like this thick

Researcher: no its thinner than this

15: oh well yeah

16: personally I would prefer to have it all together

17: yeah

Researcher: ok that's good because yeah you have these thoughts oh god will it be too much but you just want to sound it out with other people so that's good to know um I was just wondering now if you were going to have this therapy is there anything that you could think of that would make it difficult to engage with it or difficult for you to get on with it

16: you need to find out before the first meeting because some peoples especially with me its took me four years to speak to a man I always prefer to talk to females because the voices in my heads a male and its put me of completely even my brother and everything so maybe if they've got the choice of a male or female

17: yeah I agree with that I think it makes the difference because like you my voice is a male and I have had a lot of bad times with males and I do find it easier to relate to a lady rather than a man

16: I know its not always possible

17: not but if I had a choice

16: yeah Im the same with doctors I always ask for a female its not always possible

15: I mean I have had a mental liaison you know who I was great for me to go and talk to and he was absolutely brilliant absolutely brilliant and id always wanted women before so I think it just depends on the person

16: that's why I think there should be a choice

15: yeah exactly

16: if possible

19: for me your caveats around confidentiality will always be the most important because ive just had a problem with *3rd sector organisation* I used to go in there through the *name* up at *place name* you know and um I told the girl I had stopped taking a particular medication and she said well I will need to inform your doctor of that and I said im not sure you do because you said to me at the start of these sessions that everything I say to you is confidential and she said but I had to balance that against my duty of care

15: did she think you were a danger to yourself

19: I don't know I was telling her I was stopping taking one of the trivial medications and I said to her if you do contact my doctor I will disengage with your organisation and she did so I did

16: I suppose it's a tricky one really

15: yeah yes

16: I totally agree with you about um confidentiality but if your on a tablet I don't know it's a difficult one

Researcher: so the confidentiality within therapy is the rule is if its anything that presents a new risk to either yourself or others

15: that's usually how they work at *organisation* because I used to go there

19: did you?

15: yeah this is when I first started to have schizophrenia

19: if I decide that a tablet isn’t doing me any good anymore I have every right to stop taking it I believe

16: course you have like I did

15: of course you do but then there are policies where you go to your GP and discuss it with them don't you so in a way

19: I will in my own time

15: yeah yeah

Researcher: something like that wouldn't violate confidentiality in therapy because that's not putting you at risk if you had stopped taking your medication and then had become very unwell there were new symptoms and you may be felt very low then maybe they would have to break that but the fact that you have stopped taking a medication

16: that would all be outlined anyway in the confidentiality agreement surely what can and cant be said

19: that's why I like the group that we go to because its pretty well confidential

16: yeah

17: I was just going to say that I went through a stage of not taking my medication because for some reason when im not well my body says I just don't want to take anything and I would see the psychiatrist and say yeah im taking them im taking them and I was getting worse and worse and what I did in the end was when I went to see the psychiatrist I told him the truth and we had a chat about it because what was happening was theyre antipsychotics that I was meant to be taking but the voices didn't like me taking them and I would get in such a stew about taking them that I would take them and then be sick and I thought I cant keep on doing this so I had a chat with them

15: that would make you ill anyway

17: yeah that would make me ill in the end wouldn't it but I have a depot now so once its done its done theres nothing I can do about it and im much happier with that now so I think its what is right for you really

15: whereas with me I have a tendency to im really good now I have got like a 7 day week medication box now but id actually forget to take medication or I would take too much medication but now I have got it all spot on I mean I hardly drink any alcohol anymore I don't smoke and I realise how precious medication is for me and I know that unfortunately I cant get through the day without being on medication because I would be so ill and that's how bad my schizophrenia has become

17: I ended up in hospital the last time I stopped taking them

15: I was always in hospital and then theyd have to give me pure codeine because my headaches were so bad you know um so for me you know with medication it was me not taking them or me taking them too much but now ive got it just right which is great

Researcher: I wanted to pick up on something that we have picked up on a bit earlier about wanting to feel comfortable around them I wondered how much contact would you want from a therapist one idea that was put forward was that um you would have a phone call between therapy sessions to see how you were getting on some people have liked that idea some people haven’t

15: I have had that I have just had an occupational therapist um *name* and she used to um give me a text just to ask me how I am and for me to let her know that I am ok so I think that's a really good thing

17: I would rather have a phone call then a text

Researcher: you would rather have a phone call then a text?

19: so would i

15: or either shed either phone me or text me depending on her day you know because she knew that I wasn't needy but for some people they might get a bit too attached or become too needy so its got its pros and cons but it was very positive for me I actually felt like she cared which she did by doing that so you know

19: do these therapists work for your organisation? What organisation is it?

Researcher: so its sussex partnership nhs trust um and we are part of research and development so they are therapists that work in that they have previously worked in practice and they have come to be a part of research studies now

18: you know *care coordinator* hes the person that comes and sees me sometimes and I find it better because he comes to my house and he sits with me in my living room and we have a just chat and its all really informal and I prefer that a lot more than actually going to *mental health building* having to sit there walk up the stairs walk in the room sitting opposite each other and he has a piece of paper and hes writing down I don't like that I like it informal its just a conversation then you feel more at ease to actually say what has been going on

15: that's a good point

Researcher: ok I don't know what the policies are around that whether we can do therapy in someones home but its something I can look into definitely um

18: its just a more comfortable place

15: I didn't know they did that

16: I don't know if health and safety would allow it on behalf of the therapists

18: yeah that's true

16: because theres a lot of violence unfortunately that are incorporated with hearing voices

17: your support worker is allowed to come to your house so I don't know

Researcher: something I can look into so the environment somewhere comfortable. So if it couldn't be at home

16: down the pub

*laughter*

Researcher: so having choice over where

18: yeah

16: I think that though surely if it helps I know you cant abuse the system but down the park of a summers evening for an hour or walking along the seafront

18: strolling along the beach yeah something like that

16: we had a walk along the seafront with our nurse the other day because there was only two of us left in the group the hearing voices group so we went out had a walk along the sea front you know just stuff like that its like you say when your in a room with a table and two chairs no layout the last thing you want to do is sit and talk to someone

Researcher: something to think about thank you

18: I think one thing also one thing in this book maybe they should touch on more is getting your family to sort of understand whats going on

15: I understand

18: because that's the one thing you want to have the most is them to support you but sometimes you don't because of a lack of understanding

17: yeah

18: like my family are in denial still so I will tell them something that's been going on and they think its nothing its fine and that's one think that's everytime im having a really good patch I fall back because of the fact that my family are still not supportive about it and they think im doing something so that's one think that I think your family is your main stronghold so I think if you get them to also help it will be a lot easier process

15: yeah more knowledge and understanding

18: more understanding

15: because I explained to my brother um I told my brother that I was diagnosed with schizophrenia and he joked about it and he said you know he thought it was a person with um Jekyll and hyde with two split personalities and I said to him I said you are so wrong I said was you need to do *brothers name* is you need to go and get a book on schizophrenia and then you will begin to understand me better as a person and he just shut up

16: doesn't matter what you feed some people though

17: no

16: they ultimately think straight away drugs alcohol or your making it up

15: yep yep or your crazy you know they just don't have a clue they have no knowledge or understanding so family acknowledgement is really important definitely

Researcher: there is a chapter in the book for carers or families did you have a look at it?

19: I did. *mumbling *The only really significant family I have is a daughter I told her I her voices but I haven’t told her any of the things that er would be supportive to me to um know that I was going to see her on regular occasions would be much better than it being a bit of a sporadic phone call im coming to pick you up dad or something like that I find that difficult yeah so it would be more supportive if I had pre planned dates *mumbling* when she is coming back down to *place name* to do a masters

Researcher: so the family kind of work I don't if maybe that could come into the relationships topic but also not just you and the therapist working on that but taking involvement to families

18: yeah involving families a bit more or at least your significant others

16: yeah maybe like a 2-3 page leaflet instead of having a full book like this but for someone who hasn't got it just an explanation you know basic of auditory visual emphasising don't panic but something that's to the point a couple of pages I don't think you need a book book for family

Researcher: like a pamphlet

16: yeah like a leaflet that maybe comes in with a book

15: that they can refer to like ok right this is the um behaviour you know whats happening

16: because for someone who doesn't hear voices it must be really difficult to understand and believe it

15: yeah believe it

Researcher: ok so there is the carers sections in book so maybe like we are making a workbook to go along with the book we could maybe make a smaller version for carers like a 2 -3 page thing

16: yeah just a little pamphlet

Researcher: that's something we can do definitely. You are giving me lots of work to do. A few other questions I had. If we were to deliver this therapy who do you think it would be suitavble for? Who do you think our target market is? Obviously people who hear voices is there anyone in particular you would say?

17: I think for me something like that would have been good when it started when I started to hear voices and to feel down because I didn't understand what it all was and when I started hearing voices there wasn't any um sort of real help so I think something like that would be quite good if you could catch someone who just like an early sort of intervention

16: yeah

17: a bit like the book as you say I think the book would be good for someone who had just started hearing voices and had started to have mental health issues

Researcher: do you think just to play devil’s advocate do you think it could have been too overwhelming at the beginning potentially too much

16: well it depends because some people can put it by for a little while maybe im just hearing things and no necessarily think it's a voice talking to them um but I think you need to catch it especially with vulnerable people as soon as possible so that they know theyre not sorry for the word crazy and that it does happen and that your not the only because when it first started it happened to me I thought I was the only one in *county name* that heard voices

15: its actually quite common it says its 1 in every 100 people but so that's quite common isn’t it its quite surprising also there must be people in the workplace who have schizophrenia that don't actually know they've got schizophrenia or they that they know they have and have kept it quite

16: some people don't need the help they just get on with life its not distressing sometimes I wouldn’t call it lucky because I don't think mine is nice whatsoever I would take a pill and stop it right now altogether but I have forgotten what I was saying

15: we were talking about the workplace

16: yeah well some people put up with it and get used to it and enjoy it might be the wrong word but

Researcher: some people do like it

16: I know somebody that gets not down but upset if the voices don't talk to her for a while because shes accepted it as part of her life and without it sometimes she classes it as guidance and she cant go through the week without a bit of guidance and that's what she gets from her voice but I think you should be referred as soon as you start coming up with the notion that you might be hearing something maybe not a full whack on 8 or 9 week one but an initial assessment or something

Researcher: so everybody here has heard voices here for quite a while at least a few years so how do you think you would get on with this therapy is it something that you would like

15: I would like therapy but as I said to you before I would like a long period of therapy not just you know a short period I don't think it I mean its much better things work better if theres consistency and you know for if you have sessions for short period of time you can only get out so much from that um but it's about being able to trust the person that your with and um to get a good rapport going and to allow somebody to allow you to help you change you know

16: its all about numbers though isn’t it you know that's what the 8 weeks is for you know you got other people to see stuff like that

15: yeah timing I mean you can only see I think about a patient at the doctors can only see you for 15 minutes each and I get a double session which is half an hour and I feel quite lucky that I get that but I think its 15 minutes each that the person gets seen so you are a statistic at the end of the day which doesn't make you feel great

16: I understand it though at the doctors because they can refer you on to different departments but yeah its not nice having a time limit

Researcher: I think with most therapy in the NHS I know private is different but there is always a kind of end goal so it like we will see you for there are this many sessions

16: oh yeah theres an end goal but the NHS has to realise that everybody is different gentleman there might need 8 weeks I might need four lady in front of me 52 weeks you know what I mean that was just a number that came into my head

15: I know I know

16: a week and a half

15: that would be great wouldn't it

Researcher: no that's very good points very good point. One of the kind of last questions I had was if you had any advice to us so we actually do have the idea of taking this therapy and kind of trialling it out as part of a research study so we wondered if you had any advice for us first in terms of it being a research study any thing we need to think about to make it an enjoyable experience for people to help people um and also advice to the therapist that's doing it

17: the only thing that would worry me is if I had these eight sessions and that was it could you be referred to someone else

15: that's a good point actually

17: because if you had eight sessions and you thought oh

15: like you started something

17: yeah like you were starting to get somewhere

15: and then you all of a sudden its finished

16: the nhs usually you get a block set and then a gap so other people can use it and get another block set I only know that because of physiotherapy I know its completely different but it's the NHS

17: the only thing I would sort of worry about was that I started to open up and it brought up things and after eight weeks it all stopped

Researcher: so we would need to put a plan in place for the end of therapy

17: yeah yeah

Researcher: to make sure you weren’t just left

15: structure structure

Researcher: is there anything that you think, obviously the answer might be more therapy but if it wasn't more therapy what could be do to make the end a little bit more easier any ideas at all

17: I think if it was me I would even if it was telling me where there was I mean I know now, but telling me where there was a hearing voices group somewhere I could go to

15: yeah support network

16: phone numbers as well I have got three or four I never knew about in my drawer but just having them in my drawer because I know that if I am sitting up scared in my wardrobe ive got a phone call that I can phone up anytime of the day a helpful phone call

17: yeah

Researcher: all things that can be done thank you you guys are brilliant there are so many ideas that we have come away with today any other things that you have got any other advice for the research or for the therapist

18: more of this

16: don't make it so mechanical um

15: I mean obviously you need theres got to be some kind of boundary set but obviously not so strict to let the patient know what to expect as soon as they come into the you know

16: outline it all

15: its like you said at the beginning putting the cards on the table so that then therefore they have the choice of whether they want to continue or not

16: maybe emphasis that it will be an hour or hour and a half sessions but if you want to go if you don't say that some people will sit there for an hour and a half and get agitated but if you say your free to go this is your hour your free to go whenever you want just say I can provide help if you need its just little things like that can add up to make it a lot more enjoyable not enjoyable but whats the word

Researcher: making it easier?

16: yeah yeah

Researcher: again more great ideas add that on to the list. Is there anything at all anything about it being part of a research study that we need to consider at all

16: as long as they know because it sounds good because if its going to be a part of research thing it means the NHS are also trying to find better ways of doing things

18: yeah

15: I think also something that has just occurred to me is children you know is the research going to be slightly different towards children because children are like totally different kind of um human aren’t they from being an adult theyre not so tainted you know by reality or ideas they are more um pure and untouched

16: carefree

15: exactly um you know maybe the research would be very very different

Researcher: so for this particular study we will only be working with adults and see how that works and then if we find benefits there are lots of other questions we can ask from that could we offer this to younger people theres lots of other questions

15: no I think that the research done with this would be no god for children

Researcher: any other thoughts that anyone wanted to mention

16: would this this at the back is this explaining what we you are going to be doing

Researcher: that is about another study and I will talk about that in a minute

16: oh right ok

Researcher: yeah that's another study. Is there any other thoughts that anyone had

16: not from me

19: when are you planning to go ahead with it all you said you are

Researcher: the plan is actually to start in September

15: quite soon

Researcher: yeah quite soon

17: how do you choose the people that you are going to give this therapy to

Researcher: I don't chose I ask I invite anybody to come along and be a part of it um we kind of in the same way that this study was advertise we would advertise that and see if people are interested and people can come along and we have an assessment first and then if people want to take part and they do. And in the same way that we met you all signed consent forms it's the same process

15: can I be really cheeky and say that it would be really nice if we were offered a higher payment

*laughter*

15: than we get. Im just being cheeky

Researcher: I wish I could

16: that £100 is fine for me

Researcher: your not supposed to tell them that your getting that

*laughter*

16: shall I give you back that laptop as well

*laughter*

Researcher: ok thank you for your feedback. There are lots of things that I can take and make that happen

15: good luck with that

Researcher: the invitation to take part is there for all of you as well.

# Study 2 Data:

**What do you think about the idea of offering CBT for distressing voices using guided self-help?**

Sounds very feasible for the right sort of service user

Positive

I think it is dependent on the client but I think it has the potential to be effective

I think it is good to engage people with helping themselves with their own recovery

It should not be their only option. NICE recommends also access to art therapies

I think it's a great idea. After the initial workload it makes sense for the load to transfer more to the client.

A needed service. Reduce the ‘revolving door’

Depends on the client. Good for some but my clients would need a lot of support with it

I think it is a good idea but other ‘services’ should also be considered

It is a good idea

Great!

Potentially useful but pragmatically potentially challenging to deliver. Worrying if trusts then put all, or too much, or most resources into guided self-help rather than offering a range of appropriate services

Good idea. Why not? I offer self-help to all my other clients

I think it is potentially a good idea, depending on the client and their situations/history/level of motivation

In theory, sounds good. However I work in forensic mental health and service users have very complex needs. I’m not sure it would be effective for this group. Additionally I’ve found the therapist/service-user relationship essential to their type of work. I’m not sure how this would be achieved with guided self-help

Also primary diagnosis of depression or PTSD should be treated first

It depends on the severity and complexity of problems of the service user

It will be effective if I can convince the patients of how efficacious it is a treatment option

Innovative and potentially easy to administer intervention

I don't have much knowledge into this but I do believe CBT for distressing voices will be quite beneficial

I think it would be beneficial

Good as long as there is/will be an adequate evidence base

It could help increase access to therapy which is at the moment very poor. However quite a number of clients I have work with would struggle to engage – they would need more input from a worker. However I can think of a number of clients who I think could engage and benefit from this.

It would be good to review the existing literature

Evidence for CBT self-guided is lacking

It's a good idea but I’m just a bit unsure as to whether this would be effective with people who experience extremely distressing voices. But I think it's a really good therapy to use for early intervention

Very supportive

I feel this would be a … measure which would be of benefit to service users, but would routinely need to have a parallel reinforcement process

Good

I feel that this could be an effective way of "dealing" with clients whom hear distressing voices. However, due to some client’s lack of motivation, I feel that giving them a self-help guide is not necessarily the way forward. They will probably need or want someone like their support worker for example to read through it with them. This will give the client somebody to work with.

I think that it needs to be underpinned by qualitative research, given that each person's experience will be different according to their presentation and the therapist with whom they work. I am surprised to read the statement above in Section 2 9.b - "CBT can be effective for those who hear distressing voices if it is delivered by a non-therapist e.g. psychological well-being practitioner" I am a Psychological Practitioner and believe that to be called a "non-therapist" ignores the importance of a therapeutic relationship with our clients and the positive impact this has on recovery. I would be interested to understand what you believe constitutes a "therapist"?

I strongly agree in principle, but without sight of the material it's hard to say for sure.

Useful

Though this sounds like a good idea from the point of view of wider dissemination and cost-effectiveness, especially in services where no targeted treatments are available. my experience of this group are that they will require a considerable degree of support to undertake this work, due to high levels of anxiety, and often low levels of self organisation skills. I suspect that many will not work independently through the self-help materials, and will do better being guided through them with an actual person present, and supported to practice them. However, I am open to being surprised.

Any additional treatment which gives people skills to manage voice hearing is worth trying

The below questions are ambiguous and difficult to answer: - Guided self-help CBT for distressing voices would be effective for those with long standing symptoms - Guided self-help CBT for distressing voices is an appropriate treatment option - Self-help CBT materials e.g. books, would not be effective for those that hear distressing voices - People who hear distressing voices would not be able to engage in guided self-help CBT - Guided self-help CBT for distressing voices sounds like a good idea - Guided self-help CBT for those who hear distressing voices will be very effective - Guided self-help CBT for those who hear distressing voices would be unsafe That is because the answer is dependent on many different factors, especially who is conducting the intervention and the individual characteristics and circumstances of the patient. I feel strongly that an individual without training and experience in delivering psychological interventions would not be well placed to offer this intervention. It is potentially asking a great deal of the patient to go through this course given that there is a greater proportion of people who experience distressing voices who also lead chaotic lifestyles and are educationally impoverished. I worry that an inexperienced practitioner would be more likely to blame the patient if the intervention was not going as planned, further stigmatising a massively stigmatised community. I would worry that an inexperienced practitioner would struggle with the content of sessions; that they would find it hard to negotiate between what the patient wants from the meetings and what the manual demands of the practitioner; that they would struggle to properly incorporate the clinical material into a workable formulation and that these and other factors taken together could end up leaving patients feeling more helpless and hopeless than less. I understand that practitioners would receive supervision but I strongly feel that the majority of PWPs would not have the sufficient training and experience to take on this work in an effective way.

Open to the idea. Patients with poor insight and poor engagement with services are unlikely to comply without prompting/incentive

The provision of CBT within this Trust is awful. I have had my application to undertake CBT training blocked for 5 years in a row. Self-directed CBT and Computer Based CBT is cost effective and should be offered to more patients, especially those with common MH problems. The provision of Self-directed CBT in voices needs the support and backup of trained staff to ensure patient safety. This Trust does not invest in its staff; my CBT training has been done at my own expense. Despite years of highlighting the resourcing issues on in-patient wards we still do not have enough resources to give time to offer adequate 1:1 time with patients let alone CBT based interventions. My desire to be involved in this project is very high, the aim of the project is sound and patient focused but the resources to provide it are inadequate in many areas in the Trust.

Brilliant! Self-help empowers clients, helps (re)build their self-esteem and take some control over something so distressing. Besides, it seems to me that guided self-help will never be a further strain on overwhelmed resources, but a great use of clinician's time and an intervention with the real potential of bringing better quality of life to a larger amount of clients, who otherwise might not have access to full CBT.

I think this would be a good idea, especially in my area of rehabilitation and recovery. Most of our patients suffer from psychosis and a high proportion may be helped by this.

The questions are difficult to answer as they are a bit sweeping and provide generalisations. It is impossible to say whether guided CBT would be helpful in general as it depends on the client, the voices, level of distress, support they have at home, support from mental health services, any cognitive difficulties, and other mental health or developmental disorder type problems they may have, substance or alcohol use, literacy etc.

A very needed resource in the Trust. More freedom for person with distressing voices to feel confident in helping themselves. May prevent a crisis occurring if voice hearer can intervene with guided self-help before distress is too overbearing/overwhelming. May introduce individual with distressing voices to others, and reduce isolation. May increase voice hearer’s awareness on a day to day basis.

Access to CBTp is a real problem for patients. Any attempt to address this issue must be supported. I wonder if such a brief and minimally-guided intervention can be beneficial for people who have been distressed by voices for many years.

I think this is a treatment option that some patients might find acceptable and useful

At the moment I don't think there's enough evidence that CBT self-help for distressing voices is helpful for people, but that's why I think it is good that you are doing a research study to find this out. I would be a bit wary about offering CBT self-help for distressing voices as part of routine clinical practice, as the evidence isn't really there, but if it's part of a research study I think that's fine and that's the point of research.

Not keen on this - Distressing voices is not a 'common' mental health problem, PWP's would get far more complex people in at step 2 and therefore less likely to reach recovery with them.

The questionnaire is biased to the positive and does not give opportunity to express preference for other treatment approaches. I would be concerned that guided self-help is used in place of face to face therapy. I would also be concerned about it being delivered by a healthcare worker who has insufficient knowledge and skills of working with those who have psychosis. I would be concerned that treatment would be too standardised and not individual enough to effectively help.

Research needs to be done to evaluate effectiveness

I believe that irrespective of what their mental illness are, they should have options available to them to consider treatments of their choices.

Good to consider on principle but need to have understanding of depth and breadth of proposed interventions to accurately determine level of impact to nursing workload and potential benefits to service users.

I feel it would be a useful tool and could fill a large gap in the treatments we can offer.

As with all treatment options i am sure there are some patients for whom this self-help would be helpful and some for whom it wouldn't. It would also depend at the level that self-help is pitched as a large number of patients would be very familiar with their symptoms and do not need to be patronised.

Great idea, I often feel people with more complex mental health problems would benefit from simple interventions which are often unavailable to them. Obviously patients would need to be carefully selected / supported to engage and it will not be an appropriate treatment for all.

I would consider this to be helpful although unsure whether this would be aimed at clients with less distress as a result of their psychotic symptoms. Clients when floridly unwell may not be able to engage in this process on a planned and regular basis which is an essential component of effective CBT.

It could be a good treatment

I can understand wanting to encourage independence and autonomy under the recovery model but the difference between hearing voices and hearing distressing voices is a large gap. I would be concerned that the person would feel isolated rather than independent. This intervention would be better for symptom awareness and relapse prevention, also for new presentations and to build insight. Distressing voices needs more staff time and intervention and it is acceptable that the person is a little more dependent on services during these times.

I think offering CBT for distressing voices using guided self-help is a really good. It will not suit all patients, in my opinion. The receptiveness of the patient will vary over time and what else is going on in their lives. The person delivering the guidance needs have an understanding of the distress experienced by people who hear voices. It would be important that the person delivering the guidance is committed to the idea rather than a junior member of staff being told to go and fill in the book or whatever

I would need to hear about the evidence before I could comment.

It is a good idea as an option

It's a good idea.

Could be a good idea. As long as it is not going to be an intervention where the primary motivation is to save money and to have unqualified people providing CBT for a very vulnerable client group.

THE SELECTION OF PARTIICPANTS AND THE MEASURES FOR ASSESSING OUTCOME WILL BE KEY IN DETERMINING WHETHER THE RESEARCH IS VALID OR NOT

Because of the (possible) complexity of some clients, a good assessment would be required first.

In my experience you need to adapt therapy to fit people who experience psychotic symptoms so there would need to be a high level of flexibility within the approach for it to be useful.

Good for some clients, would need careful assessment. Some clients might need some preparatory work with a therapist - not sure what is meant by 'guided' self-help. If this is online, clients have commented to me that they found this unhelpful - I think it would need initial face to face - with possibility of client being followed up with face to face reviews. This client group will often suffer in silence - danger they might 'fall through the net' if not kept in touch with proactively.

It could be a valuable co-treatment

6-8 sessions would be difficult to deliver within CRHT unless the majority of staff were able to deliver the training to clients. It would be difficult for the same member of staff to deliver all the sessions as we are a very short term team with a team approach. Two members of staff might be able to work in tandem. No individual caseloads and mean stay with CRHT is 2-3 weeks. 6 weeks maximum

A good opportunity to offer clients a different approach that could be very effective for them. not everyone wants to engage in 'therapy' so this offers something clients could do in their own way during the week - some struggle to attend appointments with therapists who can be 'rigid' in their scheduling due to their own way of working

I think it would be useful if the suitability of the person was fully assessed first. Many of our client groups would struggle to engage with the CBT model so it would be important to ensure that it wasn't a "one size fits all" approach. It would also be necessary to ensure there are adequate staff to support clients through guided self-help, maybe by training the BURS team in this so there could be 24 hour support?

Do not know enough about it to comment

I think it is a good idea and worth trialling.

I think this would be a valuable resource for our clients

I think it is worth trying this but I am not sure that people will be able to engage with it effectively enough work through exercises etc. to make it an effective treatment. It may be that there is something about talking (i.e. individual therapy) about their experience that is very useful for people who hear voices, connected to the modality of their experience (i.e. hearing voices may be connected to the hearing/ speaking modality)

In my experience of working with this client group engagement is key and guided self-help may not be active enough in engaging them. I also think a therapeutic relationship is important for this group which may not be possible in such short session numbers. I also think it is important that trained therapists are working with this client group. However I agree in equal access to services regardless of diagnosis and so if there is a small subsection of this group who can find guided self-help useful then it should be tried. It should not be a substitute for psychological therapy for those who can't make use of guided self-help though.

I think this would be an excellent way of supporting clients who hear voices. However the initial motivation would need to be there for the client to take up the opportunity and this in turn might impact on the results and available data as those who are willing and able to take up the opportunity would be those who would be dictating the results.

Good idea, but wary of anything that increases workload

Great

Great idea - teams and individuals might struggle to find time / space for training / delivering and evaluating.

I think that this service is valuable for clients who wish to consider this support, and to explore options of managing distressing voice-hearing. I believe that local research can help us to understand if our local mental health resources are sufficient for client's psychosocial needs, including to offer relevant realistic hope and options of empowerment for clients, and to find if there is sufficient staff available for this service. I believe this can contribute to national research/mental health service approach. Latest findings can challenge and improve our individual and service intervention, and national research on using CBT with clients who experience voice-hearing. Within our AOT however, we have a Clinical Psychologist who offers CBT for people with distressing voices, an AOT Psychiatrist who is a qualified CBT therapist, and also works with people who have distressing voices and two Thorn Nurses in the AOT who can also work with clients who have distressing voices. Our findings in the 14 years we have been running, is that the majority of our clients choose to work through these experiences on an informal basis, and not over a set period of sessions or time.

I don’t know about the evidence base for this but yes, a good idea to look into.

It would be good for those with mild distress, or those that do not wish to engage in 'traditional' psychological therapy

I am very sorry not to be more encouraging but I see a number of problems with offering CBT for distressing voices using guided self-help in my work setting. 1. If service users are engaged enough to benefit from this therapy then it might be argued that they do not meet the criteria for Assertive Outreach Services. 2. Resources have been cut back so that we do not have time to take training or deliver this intervention. 3. In designing a broader care pathway for psychosis we would be use peer working much more. It would make sense to use a model from the Hearing Voices Network in which 'experts by experience' would lead interventions to help people who hear voices.

Probably wouldn't be that effective within an AOT population.

To have a range of options to support / treat people would be beneficial.

I tend to see people when they are experiencing an acute episode so timing of the intervention would need to be important but I think an intervention that could assist people to reduce the distress and understand the impact of hearing voices would be positive

I think it is a good idea to investigate the effectiveness of this further it think it is likely to be effective for some people - if the research can make clearer how we identify this group and how we support more clinicians to be delivering this sort of intervention to them it would be really helpful

I understand the need for generalising and categorising in mental health research but hearing voices does not affect the individual’s unique needs. It is not suitable for all voice hearing people. I believe all CBT training informs and enhances practice options and can be delivered in a less formal psycho-social way by all professional and non-professional staff. It should not be delivered in isolation and the opportunity to communicate openly is most important. CBT can be designed to promote and compliment this approach I think the goal should be to lessen the distress and help the client to feel in control of the symptoms with stress relieving strategies and responses.

Good idea.

Anything to bridge the gap in service uptake is a positive thing

As a trainee CBT therapist I and an inpatient nurse I would very much like to see this type of therapy being implemented and can see no reason why it would not be.

Valuable to have a range of tried and tested options to offer clients, particularly bearing in mind the individual's capability to engage. Problems that may occur with a guided self-help are where there may be interruptions, such as the voices themselves interrupting concentration and focus, the concurrent anxieties that they may provoke. Or the effect of long term medication, impaired cognitive ability learning disability, not only for focus and concentration, but also for processing information and transferring skills into daily life. Other difficulties that I have encountered are that the voices may be worse, and the content may be very threatening afterwards, causing additional problems, such as anxiety, lack of sleep, OCD, etc. Also consideration of running CBT parallel to other therapeutic interventions and valued activities, requiring different levels of support and or engagement, to enable individual to experience positive and life affirming events that may counter the messages of the voices, and also support / add meaning to the CBT.

I think this would offer a non-pharmacological treatment option, which aims to focus on the goals of the person experiencing voices to reduce distress and improve quality of life. I also think this client group are treated as different to others (and therefore feel stigmatised), but in my practice have found that psychosis is often on the anxiety/depression spectrum.

It will all depend on the individual and how distressed they are. some people hear voices all of the time and maybe helped while others only hear voices in a psychotic episode. I think that a lot of my clients do not have the internet and as we are hot desking i do not think that we are able to offer rooms and computers for clients to use. I would like to see evidence that this type of CBT done alone is effective rather than having a trained therapist. I would be worried that people would not be able to understand the sessions or practice them without "reinforcer " sessions with their care coordinator.

I think in this environment where there is little help on offer to some patients would be very useful. Having staff members trained in supporting people using a self-help tool would also be beneficial in order to offer more support and be able to start this with patients quickly.

I work at Amberstone which is a rehabilitation unit on the psychosis care pathway, so I deal every day with service users who hear (not always)distressing voices. I am already a Family Interventions in psychosis practitioner and I believe that being able to offer CBT as a guided self-help tool would be a valuable addition to the armoury we have to help our Service Users in their recovery.

I feel any new ideas and interventions are a good idea as hearing voices is very distressing at times and impacts on the patients quality of life.

This could be tried as an approach for living with 'voices'

This sounds good but clients will probably do better in a 1:1 therapeutic partnership with a clinician working together on this.

I think it is an obvious extension of paper based self-help guides and may help clients

I guess that I have been using a very primitive and basic version of CBT on and off over the years for individuals, however because it was not formalised or structured in a specific delivery method it has tended to be a little ad hoc; in some cases people were able to live a bit more effectively, or delay a relapse, however the client whom I was working with were all prescribed adjusted dose antipsychotic medications across the spectrum. It was very difficult to evaluate what offered the most benefit.

I think it would very much depend on the client. I think some clients would embrace it and be willing to put the work in to use the support but there are others who may struggle to do this and would not be able to engage with the treatment option. I think it is beneficial to research the effectiveness of any method which may help people with distressing voices to manage them better and reduce their distress.

Great way of managing symptoms and inviting clients to maintain their own therapy. Cost effective

Good idea but would need additional staff

The first thought that came into my mind is that it is a decision made to deliver low cost treatment which is cost effective. I am not sure about its actual value, as I believe that the therapeutic alliance is the basis of any healing process. However, it is worth giving it a try, with a control research study. Moreover, the nature of the psychosis as an illness which causes the symptom of auditory hallucinations might make it very challenging to deliver this type of intervention.

If it proves effective

Is an interesting idea, would like to know more. May be issues around engagement as many of the people on our unit who hear voices often don't have insight into their illness or are acutely unwell.

Seems like a good way to make a treatment available that people might not otherwise be able to access.

I think it would be very effective- particularly in an inpatient environment. Patients can be very reflective at this stage and willing to spend the time to engage in such treatments to improve their mental health. It would also give people the time to equip themselves with the necessary skills to then continue the treatment in the community.

Yes i think this is a valuable tool for treatment - however i feel for many it should be used in conjunction with other treatment options such as medication and alternative psycho/social interventions / therapy / mindfulness.

I think that increasing the range of psychological approaches available to those distressed by symptoms of psychosis is always a good idea - I often hear that people would like more alternatives / complements to the usual reliance on antipsychotic medication. I believe symptom specific treatments are better than diagnosis specific treatments. I think an even better approach would be person specific treatments, including flexibility and recommendations based on individual interests, values, and formulation of where the symptoms may have originated.

It is difficult to evaluate if it would be effective/deliverable without all the information. I work with Specialist Older Adults, they are far less likely to be able to engage with self-help treatments, they prefer to have face-2 face encounters

I think it depends on the client. I think consideration needs to made as to the client's ability to use and access a computer, their initial motivation 'to recover' and their belief as to the effectiveness (through other success stories). I do think however, that it is great to be able to offer an intervention that could be accessed faster than CBT treatments with a qualified practitioner. Would clinical supervision be provided?

I think it's fine to try and may help us be more effective in offering these clients equal levels of support. However, I can see there might me challenges in engaging this group.

I feel that there are some clients that may be able to get benefit from this and some who would not, but that is the same with all types of therapy offered and this is an under treated group.

This is a good idea. It would be good to learn from the existing self-help materials e.g. produced by Ron Coleman or by the hearing voices network. We can think of it within the framework of different layers of CBT for psychosis. I would be interested in considering using this as part of a recovery course like we do in the WRAP courses. Be good to consider IAPT psychosis and recovery focused outcome measures (see IAPT website and ImROC website for Geoff Shepherd paper on outcomes: - personal goals - CHOICE - SWEMWBS and distress and powerfulness of voices

It maybe helpful

Very good idea, although i think there may be more challenges in engaging AOT clients in self-help interventions.

with limited resources this sounds like a good idea in principle, especially if it can be shown to be clinically effective.

Excellent in principle.

**How willing would you be to be involved in the development of guided self-help CBT for distressing voices?**

Would be willing but may not have the time

I think it would be very useful and interesting

I would be very willing to be involved

Not unwilling

I personally would be very willing

Yes in the future after undergraduates clinical training

Yes, willing depends on time commitments

8/10

Very willing

Very

I would be willing to be involved assuming time restraints permit

Yes

I would be willing to be involved if time could be freed up to do so

I don't have the time

I would be keen however I think it would be inappropriate with my client group

Very!

By finding out more

Depends on time commitment

I would be interest to help in developing this. I have an interest in working with psychosis and have experience of this client group (PBCT for voices group research trial, work in EIP service, clinical experience delivering CBT for psychosis in secondary care).

I think face-to-face appointments with a trained clinician is more appropriate

I’m not sure

I was involved in a similar project of telephone guided self-help for psychosis

No as this would worsen symptoms and increase risks

I would be willing to help research on it

The only issue I have is that I am unable to take on extra work as I am working at far above my capacity currently

I am interesting is aspects of this area having previously received training around CBTR with psychosis

Yes

I would be more than happy to be involved in the development for CBT, it will be useful for myself as a support worker so I am able to support those with these issues on a more professional level. would be happy to go on training days to learn more about this.

I am positive about such a development and would wait for research outcomes to prove the efficacy of this approach.

Very.

a little

Not enough time to do the work that I need to do at the moment, so would not be able to do so!

I wouldn't want to do this as no one in psychological services, nor psychologists in teams will ever accept responsibility for implementation of the Mental Health Act or Safeguarding Vulnerable Adults, and it takes up a substantial amount of time in my working life. I don't therefore have time to do psychological work alongside statutory work.

Would be interested in the outcome of your research but don't have time to participate in a research project at this time.

The provision of CBT within this Trust is awful. I have had my application to undertake CBT training blocked for 5 years in a row. Self-directed CBT and Computer Based CBT is cost effective and should be offered to more patients, especially those with common MH problems. The provision of Self-directed CBT in voices needs the support and backup of trained staff to ensure patient safety. This Trust does not invest in its staff; my CBT training has been done at my own expense. Despite years of highlighting the resourcing issues on in-patient wards we still do not have enough resources to give time to offer adequate 1:1 time with patients let alone CBT based interventions. My desire to be involved in this project is very high, the aim of the project is sound and patient focused but the resources to provide it are inadequate in many areas in the Trust.

My role at work is more geared towards teaching and supervision rather than direct clinical work. However, in this capacity, I would be very willing to be involved in any way I can.

I would like to be involved, but it would depend on how much time would be needed for the training etc. and whether my manager could backfill the post

Quite interested Peer / service users should also be asked for their involvement.

Very willing, though have had no formal training in CBT.

I would be very keen to be involved. This is an exciting learning opportunity.

Not willing. I am nearing retirement and beginning to 'close down' work outside my caseload, for example extra training.

I'd be very willing to be involved, I think it sounds like an exciting development and that if it is helpful for people it could make access to CBT better for this client group who often don't get offered therapy.

Not willing.

I am willing to be involved but in principle do not greatly support the idea. I think that those with psychosis should have the same rights of access to a CBT service that primary care clients have. I believe CBT is better delivered face to face and by a qualified practitioner. I believe that practitioners working with psychosis should be experienced mental health professionals with sufficient experience, training and skills in this area of work. I believe it to be a very specialised area requiring an advanced practitioner.

very

Again, further information required to determine level of involvement and to consider feasible involvement from perspective of nursing team.

I would be very willing to be involved.

Unfortunately I am currently on maternity leave. I would otherwise have been interested to be involved,

I would be willing to be involved to some degree

It depends whether it is within secondary care or primary care - I work within secondary care. If it is the later I would be interested and willing

I would be interested to learn more about it and to hold an awareness if it were to be taken forward and promoted. If this were part of somebody's care plan and I were to meet them during the course of my work I would need to be able to support the plan they have and the interventions in place so that there is consistency between services. One could say that it would be essential if it were an intervention in common use.

It is something that would very much interest me but due to time constraints would not be a feasible option

I would be willing but I am not sure practically how that could happen.

Interested but pressures of work would make it difficult

I would be willing to be involved, although current work commitments may make it difficult.

I have to be careful with time because of other work commitments in my workplace. However, if it was linked to the Voices Clinic in secondary care it may be possible. Or on a Bank Therapy basis for Wellbeing outside my working hours.

WILLING IF TIME CAN BE FREED TO ALLOW FOR IT

Willing but limited by service changes (reduced staffing)

Although I have some experience and interest in working with distressing voices I do not particularly use a formal CBT approach so am not sure if I would be that helpful.

In theory, depends on time demands.

willing

I would be willing to - but only if the approach could be used within CRHT

I would really like to be involved, however the pressure on the Trust and our team are overwhelming at this time so it is difficult to find space to do further work as we are already having to prioritise and stop some activities

I would be willing to be involved but unsure in what remit as unsure of how it would be developed.

willing if trained and work pressures allow

I would be willing and very interested it is just about fitting it in with my current workload

I would be interested in hearing more about this.

i would be very willing

I would be willing to be involved.

I would be willing to trial it, but as I have no training in CBT and a tentative grasp on it, I would not feel able to contribute in any knowledgeable way.

Am currently involved in another training, but yes in principle.

Very

as a CMHN I'd be willing - except for vast array of current commitments - if I could be given a reduced case load or less pressure to take on new clients and if time / space (for thinking / training / developing self) was recognised by the trust as important part of the role.

I would be willing to promote the service locally, including to our service users, but do not feel that i have the time or due to my individual and team work, any time to commit to being involved in this service.

I would be willing to be involved with the supervision of practitioners, not the delivery of the intervention.

This does not seem possible with our current resources

I am interested however the workload in the ATS TEAM IS HIGH SO IT WOULD NEED TO BE PROTECTED TIME AND not to be an add on to what I do but with no extra time to do it.

Yes - willing to be involved

Yep

I would be very willing but limited in time

Very willing.

Very

Work load at present does not allow any commitment in this respect.

Very willing.

i would want to know more information first as our case loads are very high and it is really hard to find extra time for research and training etc.

receive training, encourage other staff to do so

I would be willing to undergo the two days training to equip me to deliver the guidance for using the self-help tool, but as a Charge Nurse, the time I could spare to becoming involved in the development would be limited.

I would be very willing

Having never worked with CBT as a treatment before this would be very interesting.

Interested - I have a first class degree in CBT and Family Work for SMI.

Pressure of work would be the only obstacle to helping support implementation of self help

this area does interest me, though as I work for a team that has clients on caseload for a limited time period, we have very limited scope for working with individuals hearing voices. borderline personality disorder seems to be the most prevalent group experiencing 'voices'

I would always appreciate training in ways to help people with distressing voices and would try to implement these ideas where possible and appropriate to the client. Whether it is part of my job description or where I would find the time for this is another matter...

I find it interesting but do not think I have the time or the capacity to offer full input

It's not something I personally would want to pursue at this time.

No capacity but interested

It sounds interesting and I would like to be involved, however there may be problems with my particular role, as I have had consistent difficulties in developing my current role (Support Worker) in a way that included any therapeutic interventions and training (apparently there is a "Conflict of interests" as therapeutic interventions are not a priority for my role, which needs to be mainly practical and I am not insured to deliver therapeutic interventions).

time and caseload permitting

Very willing- I think it's a very interesting concept and also a very cost effective of administering psychological therapies.

Very interested but would depend on my current case load.

I would be very willing to be involved in this.

I would be happy to make the time to be involved in trialling this intervention or to be trained to deliver it after research has tested its effectiveness.

I think as an acting manager at present it would not be practicable for me to be involved

I would be interested to learn a new skill, I also think it would provide me with the confidence I would like to guide an intervention with this client group.

As I'm a trainee on placement for a year, it seems unlikely that I would be available to do this, however if it was possible I would not be opposed to being a part of it.

I have no formal CBT training and do not think offering self-help CBT fits with my current job

yes I would be interested and think it would fit with my job role

I'm very interested in it, but not sure it would be as relevant for AOT population so given my part time working i don't feel this is a priority for me.

willing but I am not a trained cbt therapist for psychosis

Very, but I am dependant on managers who may be pressured to achieve targets and may not see interventions such as these as essential. It's pivotal to have leads and managers on board and offer protected time to learn and use these interventions.

**How feasible do you think it would be to implement guided self-help CBT for distressing voices in the trust?**

I’m sure it would be challenging but perhaps also attractive to trust/CCGs from a cost perspective.

Depends on resource

If people commit to it with a reasonable amount of time for training it would be feasible

Fairly

If the case was made for its efficacy I’m sure there are a lot of practitioners who would gladly have a ‘proven’ tool at their disposal

Feasible for the lucky few

Yes feasible

6/10

Should be but would need to get staff on board

Depends on resources but potentially yes

No idea

I think it is feasible if appropriate training and support are available

From what I’ve heard it could be a lot of work to get it started but very effective in the long run

Not sure

Very

Not sure

If it could be included in our psychosis care pathway and staff were trained in delivery I think it could be implemented. A caveat would be fitting it into care coordinators case loads. Appointments are often inly every 3 weeks due to high caseloads this may not be frequent enough. However a telephone call between sessions could help increase frequency.

I think it would feasible

Need commitment at different levels. Need good training and supervision (on-going) and management commitment to this

Due to budget cuts and staff workload, probably not that feasible

If we plan and protect peoples time in order to carry out the work

Very feasible

It would be feasible if the administration was kept to a minimum

It will depend on staff attitudes and capacity

I think as a trust it would be fairly feasible to bring in these guides.

As above.

Entirely feasible, as long as resource implications are addressed.

ok

I think it would be feasible.

I have no idea. All staff are asked to do unrealistic amounts of work, and this may simply need too much time.

As per my answer to 10. it would depend on what "implement" means. If it meant rolling this out with a team of PWPs I'm sure it would be logistically and financially feasible. But I'm not convinced it would be ethically or clinically feasible. I am willing to be proven wrong by the data. If "implement" meant using MDTs with experienced psychological practitioners then, although this would be the ethically and clinically superior method, I doubt it would be feasible.

Feasible as self-guided CBT less costly than guided CBT. Certainly good to have the resource available as an option.

The provision of CBT within this Trust is awful. I have had my application to undertake CBT training blocked for 5 years in a row. Self-directed CBT and Computer Based CBT is cost effective and should be offered to more patients, especially those with common MH problems. The provision of Self-directed CBT in voices needs the support and backup of trained staff to ensure patient safety. This Trust does not invest in its staff; my CBT training has been done at my own expense. Despite years of highlighting the resourcing issues on in-patient wards we still do not have enough resources to give time to offer adequate 1:1 time with patients let alone CBT based interventions. My desire to be involved in this project is very high, the aim of the project is sound and patient focused but the resources to provide it are inadequate in many areas in the Trust.

My experience with staff and training is that both staff and managers who support them are very willing to engage in training which directly contributes to clinically supporting clients. Such intervention, in my view, would be very welcome.

I think it would be a cost effective way of reducing severity and length of stay in in-patient units.

Depends on client group - see above.

I feel it could be feasible with willing participants and well managed weekly sessions. Continuity for the voice hearers ,with a good follow up programme to help maintain their guided self-help programme after session end. A possibility for participants to have a person to contact after if they find they are 'stuck' in practice at any point.

If the intervention were found to be beneficial, it would make a valuable addition to the care pathway.

I think if an allocated person was offering this treatment and it was offered with no 'strings attached' then it could be really helpful. Trying to offer specific interventions as part of a 'care coordinator's catch-all role' would I think be difficult. Awkwardly though, it is the very patients who might benefit from such an intervention, whose most contact is likely to be with a CCO. Asking patients who are distressed by voices to attend for 'therapy' is likely to be difficult. An 'assertive approach' is likely to be needed by the practitioner who is going to deliver the intervention (e.g. a Psychologist)

If it proves to be effective I would think it would be very feasible to implement in the trust. I like the idea in principle of training up PWP-type workers in the ATSs who could offer these kinds of interventions. This could help to really improve the accessibility to service users to effective and helpful psychological approaches without it requiring as much resource as it would to be fully compliant with NICE guidelines for psychosis.

Not very feasible

I think it could be set up and trailed reasonably easily.

Very

All things possible if there is a positive approach with good research evidence based practices.

Could only be determined with trial runs in a number of localities to determine infrastructure and potential roll out to the rest of the trust.

I think that enough people will be interested and that in the long run it will improve services in an area where it is sorely lacking.

I believe it would be feasible, but it will require enthusiasm at ground level rather than implemented from above. It will also require support from senior level - for example for people to attend supervision, and I suspect the reality of service pressures will make it challenging for services to support this.

I think it is feasible but consider this best delivered by clinicians with a formal training in psychosis. Staff who deliver step 2 and 3 interventions are not in requirement of clinical training in psychosis and so would not have the knowledge to recognise this condition and the signs of relapse and risk.

It seem feasible if staff can be found with time to implement it

I worry that it would be an opportunity to reduce staff time with the service user and save money. It would be ideal as a SMILES care plan to bolster independence and autonomy before discharge to the GP but I do not feel that it would be appropriate as a blanket intervention in all other teams. Also, in my experience, the socio-economic factors in many mental health cases would complicate whether the person would be able to engage and understand any written work to a meaningful level.

I feel that all staff are under huge time pressures but see that this programme could be seen as an investment in the patient and may reduce distress and possible relapse in the future. Sadly, all too frequently, the trust seems unwilling to encourage preventative interventions to reduce future problems

This would depend entirely on whether or not the trust viewed it as a cost effective solution.

Given the limited resources and pressure on staff, it would be difficult for people to commit to the training

Difficult unless properly resourced.

Not sure because I don’t really know what the project involves yet.

yES - IT'S A MATTER OF RESOURCES WHICH IS BEYOND MY GIFT AS I AM NOT A BUDGET HOLDER!

As above ~ would depend on staff being freed up to implement this as part of a pilot study.

It would depend on who does it and how it was done. I think skilling up staff to offer interventions for those people that they see who hear voices would be possible and useful but making it very formal, with lots of demands on the staff, might not work so well. There is also the hearing voices research that is currently being undertaken in ATS which would also limit the number of people available to support the research.

If studies have found that results of successful implementation means the sufferer is less distressed and needs less contact then people might get on board. Also, need support and presence of medics.

a trial to test feasibility first would be a good idea

Yes - but I wonder if it would need to be delivered to clients who have one named worker.

It would be feasible of the Trust signed up to this - allowing professionals to attend training and ensuring protected time to then do the work with clients and attend supervision - again due to the current pressures this will be very difficult to implement

only feasible if enough staff are involved

I think it is very feasible if you target the right practitioners and the people who do the training understand and appreciate any time commitments involved prior to undertaking this and they and their managers sign up to same.

This would be feasible with the correct staffing levels/skills.

I think that resources are available and staff are willing

I feel in my work and with the client group I currently work with it would be feasible.

Feasible if implemented as a policy

Easy

at present and personally - not at all within the trust - it should be happening and i believe it is feasible

Yes, i think it is possible, but clinicians involved should have time protected for this, ideally ensuring that they do not have to do this in addition to current caseload, and have time to attend course learning and supervision.

Very- if sold as a way to reduce people's workload! I think given how stretched people are thought it could be seen as 'another thing' to do

Very feasible, if funding is available for the required resources

There might be structures and services in the Trust where this approach could be developed and delivered.

It needs to be well planned and communicated fully in the trust and with all the teams.

I think there are 2 ways of looking at this - first is workload. Increasingly clinicians are wanting to develop ways of working with people who hear distressing voices and time would need to be incorporated into a person’s week. Second is the culture and medicalization of symptoms - this could be a challenge in some but not all teams.

I would think this is possible, at least initially to set up as research and then roll this out further

It seems to me that as psychosis does not produce results or turnover suitable to corporate organisations it will remain the poor relation within services. I would like to see self-help CBT available for referral to care coordinators and care teams as an integrated element of care plans.

Very difficult in our team due to resources.

Dependant on resources across areas, but may be useful to roll out as a course available from recovery college.

I think there will be resistance from both practitioners who rely solely on the medical model and also potential resistance from psychologists who disagree with psychological therapies being brief and delivered by other professions.

as above access to computers is a problem and having to offer extra support may be a real burden when we already have a lot to do. Who is going to monitor people? If people do not complete or do not find it helpful will they then be offered individual CBT asap?

I can only speak for this area, I think this could potentially be a very useful time to do this as there are a number of other changes taking place

I feel sure that once the training and tools have been developed, it would not be difficult to implement, either in in-patient services or in the community.

With good time management very feasible

Unknown

Depends on the support in terms of resources provided by the trust.

Lack of access to IT systems may be a barrier Work load of self and staff here would make it very difficult to attend training information sessions and allocate time to seeing a client if that would mean extra time on top of that being allocated at the present

I think that there would have to be an arrangement by which all practitioners managers agree to backfill, or release them for an agreed number of hours in order to successfully implement this scheme

It would be feasible to some extent and self-help would probably support the efficiency drive but it would take a large amount of initial effort and I do not know of many teams who have time to do this. I would also want to know more about how this is monitored.

Yes I feel it will be feasible

Only if there were enough staff to implement it

For people with low severity of symptoms it might have some value, as any psycho-educational interventions that informs clients and enables them to gain more control over their symptoms.

may require extra resources

Very feasible. As with all things it would take some adjustment but would be a very handy resource to use once staff learned how to use it/introduce it properly.

I think it would be very feasible as long as this new tool / intervention is well put together, the use of it is thoroughly communicated across relevant teams (incl. aim, purpose, instruction for use, research findings for validity / effectiveness), opportunity for service user and staff feedback and for management/leaders to be behind the implementation to support staff - i.e. time to be introduced to the tool, training if needed and supervision support if needed.

I believe that if enough people agree that it is a good idea to try, and if research can show its effectiveness, then clinicians will make the time in order to implement this approach, even given the demands services are currently facing.

I have no idea in other areas but unlikely in SOA

I'm not sure as I don't know the trust well, but IAPT seems to be working elsewhere, so why not?

should be quite possible - staff are busy but this might well be welcomed - half of people in secondary care have psychosis yet only 10% receive psychological therapy

Feasible definitely in recovery services and perhaps in AOT

no reason this should not be feasible

Very if attitudes towards low intensity interventions start shifting.

**How should guided self-help CBT for distressing voices be evaluated?**

RCT

Interviews, as well as self-ratings from participants and maybe statistics of those who found it helpful or unhelpful

RCT and feedback qualitative research

Mixed methods – qualitative and quantitative. At this stage perhaps some single case statistics.

Symptom reduction

Multiple measures (quantitative) qualitative as well, focus groups

By regular routine outcome measures in sessions and follow up

RCT and service user and staff feedback

Qualitative and quantitative methods with clients as well as feedback from client’s network i.e. staff and family

Validated scales, including mood and anxiety scales

I like the idea of qualitative research in addition to quantitative measures i.e. interviews with clients who have had this intervention

Qualitative measures should be used alongside more quantitative measures to understand patients experience and the process of recovery

Mix of measures for anxiety and psychotic symptoms in conjunction with measure to evaluate wellbeing and distress and ability to carry out activities of daily living.

Patient preference, levels of distress, social functioning

Over a lengthy period and by linking in with teams involved with client

Distress, quality of life, functioning, relationship with voices and qualitative feedback – questionnaire, including the family and friends test or interview.

Face-to-face interviews and questionnaires

There are existing recovery measure that would be helpful

RCT

Should be a range of measures which incorporates both qualitative and quantitative analysis such as semi-structured interviews, psychosis (hearing voices) measures, patient feedback and progress

Peer support work to follow up with service users

Control study. Qualitative narratives

Using a multifaceted tool that recognises lower symptoms and quality of life as core domains

Levels of distress, relationship to voices, quality of life, symptom severity is less important if distress level is reduced

I think this should be evaluated on a 6 month basis.

Patient self-report of outcomes.

scales

Mixture of approaches, including pre and post measures to establish effectiveness, to include levels of distress around voices, occurrence of voices, quality of life, possibly anxiety and depression, ideally using control group. However would need to also use qualitative measures based on interviews, so that can drill down into actual experience of going through the process, and identify what works and what doesn't, and find out where modifications can be made.

I would recommend that evaluations not only look at the gains made by patients but also at the harms done through the intervention. This is a difficult thing to collect because of issues around demand characteristics, chasing excluded cases etc., so care would need to be taken. I would strongly encourage service users to have a voice in the development of the intervention and an opportunity for them to consider the issues surrounding the intervention. There is a growing pressure for complex interventions to be manualised and rolled out by inexperienced practitioners. It is seen as a cost-saving exercise, getting "more bang for your buck". However, complex interventions are complex because they demand flexibility and ingenuity on the behalf of the practitioner for them to be effective with the particular individual in front of them. Codifying complex interventions means that those individuals that do not fit the manual are excluded and stigmatised. Using inexperienced and under-trained practitioners increases the risk of poor outcomes and a revolving door effect on service utilisation. Although it may not sound like it from the above I genuinely value innovation in this area and I certainly agree that CBT for voice hearing can be an extremely effective intervention. Also, I would welcome more access to this kind of treatment for people who often have very little opportunity to work psychologically with their distress. However, I simply feel that this approach needs careful consideration and a common-sense approach to dealing with the above raised issues.

Randomised control trial - PANSS score reduction etc.

Patient feedback both in terms of quantitative and qualitative feedback at 1,3 and 6 and 18 month post completion.

An RCT would be ideal.

Using pre-course questionnaires and outcome measures

In many different ways including qualitative and quantitative,

At various intervals through the designated 6-8 weeks, and for set times after to receive input of how individual is coping .

Within an RCT - with measurement of the outcomes that have most meaning to patients.

I have no idea. I think all 'evaluation' is awkward (after a teaching session/after a consultation). I think it borders on being meaningless.

I think it should be evaluated using a mix of qualitative and quantitative approaches - I think it would be important to ask participants what they think about the intervention but I also think that an RCT at some point would also be really important, because without this you wouldn't know if the self-help intervention was any more helpful than nothing at all.

Limited Pilot study. A RCT should compare guided self-help with equivalent number of face to face contacts with a qualified Cognitive Behavioural Therapist

Listening to the experiences of clients

The service user self-evaluation of how it makes a difference to their lives and the carers observation and perception of their cared for quality of life.

For trials, careful analysis of time and expenditure set against feasibility of running a 6 to 8 intervention programme for service users who may not be an inpatient. Is this more likely a service for day centres (both community and inpatient) activities or community settings due to high turnover of beds and average ward stays.

Hustig & Hafner - good simple measure Psyrats Qol measure (adapt the w&sas?) Must include something individualised for personal goals, since living better with voices will require very different adaptations for different people - i.e. might be feeling able to attend a specific social event or part of town

Via both qualitative (interview/assessment) and quantitative (questionnaires) measures that can be reviewed over the course of the treatment.

Research both qualitative and quantitative

Always ask the patient. If they do not feel it is working then it is not working.

Ask the patient if it has helped reduce the distress caused by the voices

I think you asked this in the questions above.

By qualitative and quantitative measures.

Not sure because I don’t really know what the project involves yet.

USER FEEDBACK VIEW QUALITATIVE INTERVIEW AND FUNCTIONAL ASSESSMENT IS ESSENTIAL, SDQS ETC ARE V BLUNT INSTRUMENTS FRO THIS SORT OF PROBLEM

Qualitative evaluations { decrease in distress, ability to engage in meaningful activities }

In my experience voices do not go away but quality of life can be improved - if this was the aim of the intervention then evaluation of this would seem to be more relevant and likely to capture any changes.

Mixture of qualitative and quantitative measures, including perhaps telephone contact.

by both the client and CC

RCT although the 'gold standard' would probably not be feasible and probably not ethical as the benefits of guided self-help are likely and would it be right to prevent patients receiving a beneficial treatment. It would be difficult to get two 'matching' groups of patients as well. A mixed quantitative / qualitative approach might work. Solely a qualitative approach would rely on too few patients and can be very time consuming

RCT

Individually, by the person’s ability to manage their symptoms. This could be done by interview, collateral information such as reporting/observing of symptoms and reliance on PRN medication. Further it should be re-evaluated after a period of time to see how sustainable this is.

Speaking with service users and staff involved.

Follow up evaluation.

By staff and patient feedback.

I think there should be robust evaluation based on measures of outcome, rather than just client’s perception of helpfulness or their willingness to say it was helpful.

Carefully and with an awareness of the fact that those who would agree to take part will be driven my different stressors and motivators than those that decline or are unable to take part.

Post completion interview/ questionnaire?

As previously suggested.

Mixture of self report / qualitative and team / family perspectives. Questionnaires? Measurement scales done over a period of time (perhaps weekly scale for a month) to get an average or idea of direction (i.e. are skills sustained over time, do symptoms cont. to improve (be alleviated) or as time goes on and if skills are not used or maintained do symptoms (distress) return?

Through audit of research proposed but also audit of such service already being offered and used by current local services.

See answers above. Pre, during and post measures plus follow up. Ideally mixed methods design (qualitative and quantitative data)

Measuring level or distress experienced by patient qualitatively and quantitatively

Again I recognise that I am not being very encouraging but I tend to see the problem of distressing voices less in terms of a symptom which can be measured and then reduced through therapy. I see it as connected with life problems and it might not be the priority to deal with it in isolation. Sometimes it might be a good outcome if voices stop, but people can then feel lonely and more distressed. Measuring outcomes would perhaps make more sense in terms of quality of life, or perhaps about the quality of a person's relationship with their voices. I believe it is important to help people develop their own understanding of their experiences, but this does make it difficult to construct a shared measure in which all diverse experiences can be included

NO IDEA

Yes - cannot understand the effectiveness if it is not evaluated

Looking at QOL measures, patient satisfaction with the intervention and perhaps some measure of structured use of time

Informal interviews with the individuals over a period of time long enough to see how they have continued to use the techniques they have learnt (6 monthly repeat assessments)

Various client measures around distress, functioning, anxiety/depression. Care co-ordinator/other experience.

On an individual level, enabling the individual to select from one that they can best work with, and on a Trust level as an outcome measure.

Self rated measures.

From a base line assessment at the beginning of the source and then a review to see how symptoms have reduced half way and then a final on line questionnaire and follow up two months later to see if people are using the strategies they have learnt.

Patient feedback

There are a variety of tools available to evaluate how the CBT has impacted on Service Users quality of life and symptom reduction

Feedback and interview of patient

I am sure there is a 'tool' that is in place for this evaluation.

Through qualitative and quantitative follow up, over long time period.

Pre use evaluation followed by mid, post and several month later assessment by external assessor

I think that there are a number of measures; subjective measures are important as if the individual receiving any therapy even medication; without faith or hope the intervention will fail for various reasons. There could be a peer review from colleagues not attached to the CBT link additional to the client themselves.

By looking at how much it helps people manage their symptoms and reduce the distress they cause. How able people feel it is to engage with would also need to be monitored, as well as evaluating how staff felt it helped their clients.

SUDS scores of client’s pre and post course with a follow up 3 months and then 12 months

Interview before and after and six months later

I think the main indicator would be the ability of clients to cope with their symptoms and the stress level related to their experience of hearing voices and it might be helpful to measure this. Other symptoms like anxiety, depression and social inclusion or the ability to engage in meaningful activities may be pertinent but in some cases they may be misleading. For example, I worked with a client with severe symptoms of schizophrenia some time ago. His quality of life improved at some stage, but his inner world also became more distressing at the same time and as a practitioner I was not always aware of this, as the client did not feel comfortable talking about this.

All the ways above suggested, symptoms, quality of life etc.

It could be evaluated in a controlled way using an RCT- measuring symptoms before and after. It could also be measured by randomizing entire teams- so some half the teams would receive the training to implement it and compare this against teams who haven't. This could create a more robust way of measuring staff attitudes towards working with clients who hear voices in terms of how equipped they feel to support them, if they feel their support is effective etc. Then also measure the actual symptoms & opinions of the patients who receive the treatment. It might also be worth testing the effectiveness at different stages of illness- e.g. acute care (inpatient wards, crisis teams) against those under community teams such as ATS. Then gather data about patients engaged with it, how easy staff found it to implement etc.

Service user feedback and staff feedback. Self-reported measures of symptoms experience - psychotic and non-psychotic symptoms.

I think the most important thing to measure is whether the intervention reduces the level of distress and impact on the person's life caused by hearing distressing voices.

By measuring symptoms, distress, level of functioning - quantitative and qualitative

I think, as suggested by current research, that outcome measures should be based on what a client would like to achieve (for example, lessening distress, increased problem solving ability, social inclusion) as oppose to symptom reduction.

A short qualitative semi-structured interview and possibly one brief questionnaire.

Follow up interview

The evaluation should be co-produced by researchers, clinicians and people who hear voices themselves. It would be good to consider IAPT psychosis and recovery focused outcome measures (see IAPT website and ImROC website for Geoff Shepherd paper on outcomes: - personal goals - CHOICE - SWEMWBS and distress and powerfulness of voices and also qualitative interviews with both people receiving the intervention and staff and maybe with the voices too!

I think distress related to voice hearing experience is primary; also satisfaction / experience of the intervention, quality of life. Perhaps clients identify own goals at start of intervention and rate pre/post intervention.

Through carefully designed outcome measures

Measures of distress, quality of life, involvement in social activities, family, leisure activities, the community, reconnecting to one's life, choice questionnaire, BAVQ, psyrats (although I imagine in several cases the voice would not disappear, the ability to cope with this voice and distress caused by it would be likely to change), etc. ideally via RCT.
